# Inhibition of Dermatan Sulfate Epimerase 1 by Substituted Glucuronic Acids

**DOI:** 10.1021/acsomega.5c10686

**Published:** 2026-02-27

**Authors:** Roberto Mastio, Isolde Zuleta Sjögren, John Dahlquist, Anders Sundin, Gunilla Westergren-Thorsson, Sophie Manner, Emil Tykesson, Anders Malmström, Ulf Ellervik

**Affiliations:** † 5193 Lund University, Department of Chemistry, Centre for Analysis and Synthesis, P.O. Box 124, Lund SE-221 00, Sweden; ‡ 59568 Lund University, Department of Experimental Medical Science, P.O. Box 117, Lund SE-221 00, Sweden

## Abstract

Dermatan sulfate epimerase 1 (DS-epi1) is a key enzyme in the biosynthesis of the glycosaminoglycan chondroitin sulfate/dermatan sulfate, catalyzing the conversion of glucuronic acid to iduronic acid at the polymer level. Chondroitin sulfate/dermatan sulfate chains are found on at least 32 proteoglycans, many of which are implicated in human diseases and syndromes, as well as in both malignant and normal cell development. DS-epi1 therefore represents a promising target for drug development, and recent structural studies have provided insights into its active site and catalytic mechanism. Here, we report the synthesis and biological evaluation of inhibitors based on 1,4-disubstituted glucuronic acids. These compounds were synthesized from glucose through a divergent approach, yielding 19 derivatives that were tested in a functional assay. To explore the importance of the carboxylic acid moiety, we also tested the methyl ester analog and the analogous xylose derivative. The most potent compound exhibited an IC_50_ of 42 ± 4 μM. Molecular dynamics simulations showed a strong interaction with the active site of DS-epi1.

## Graphical abstract

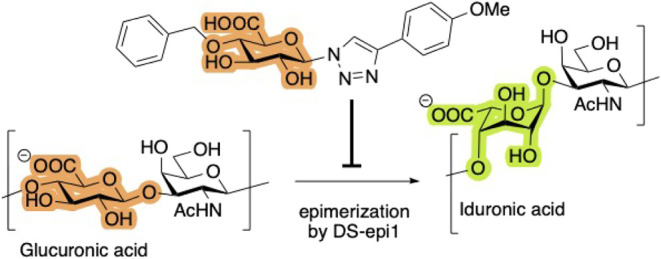

## Introduction

Information-carrying biopolymers—proteins, nucleic acids, carbohydrates, and lipids—are essential for all living organisms. Despite significant recent progress, polysaccharides remain the least understood biomolecules among these major classes. Many cell-surface polysaccharides are synthesized through nontemplate-driven processes, producing a vast and sometimes temporally variable diversity of structures. This structural variability enables an almost limitless array of potential messages—far exceeding the coding capacity of nucleic acids and proteins. Rather than forming a distinct universal code, certain cell-surface carbohydrates instead present specific information-bearing sequences that are crucial for cell–cell communication.

Proteoglycans (PGs) are large macromolecules, composed by a core protein covalently linked to glycosaminoglycans (GAGs)—long, linear, negatively charged polymers of repeating disaccharide units (Figure 1a). PGs are located both on the cell surface and within the extracellular matrix, where they play important roles in regulating growth factor signaling, inflammation, angiogenesis, and cell–cell interactions.

**Figure 1:**
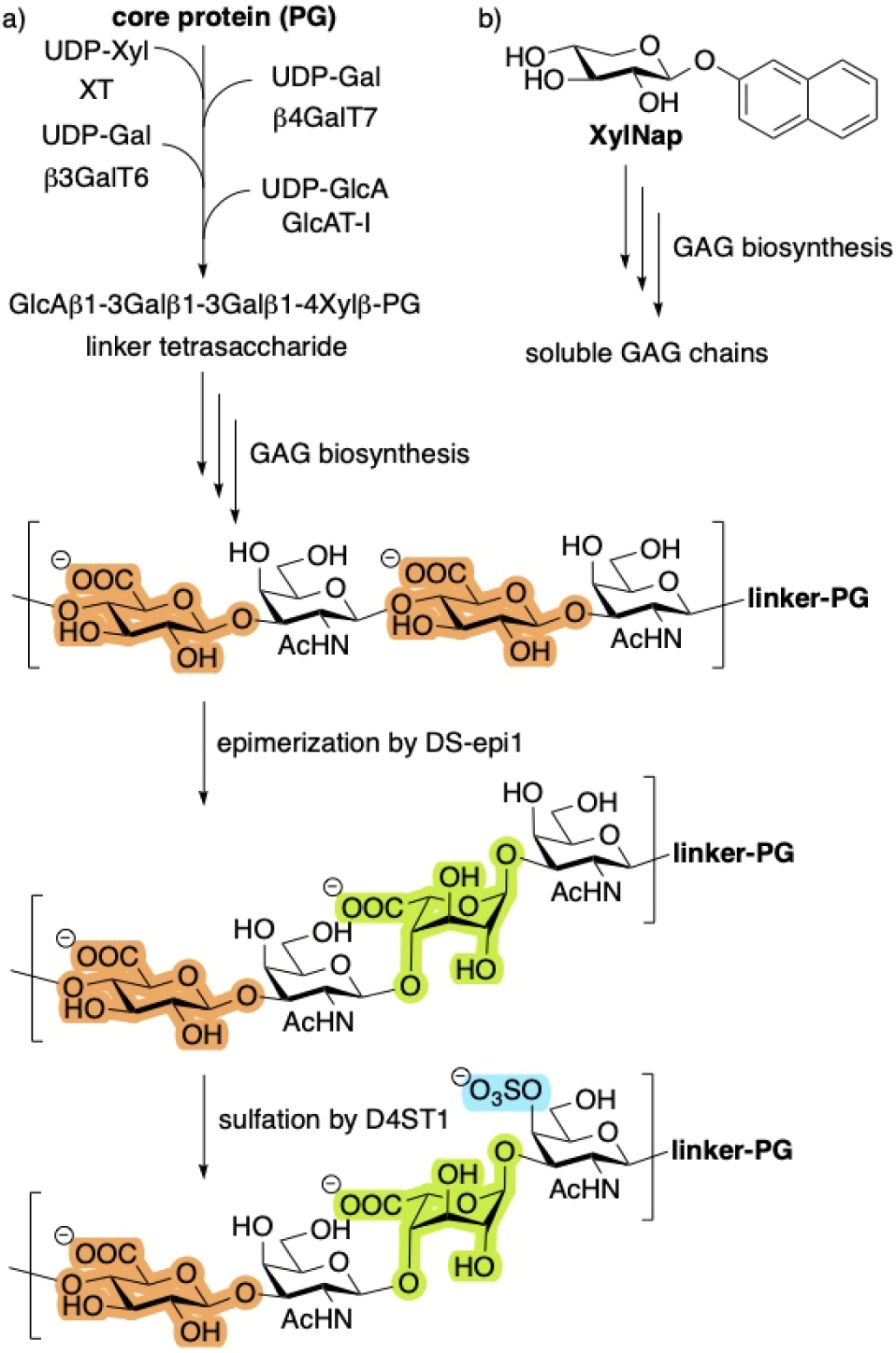
a) Biosynthesis of GAGs is initiated by xylosylation of a serine residue of the core protein, followed by addition of three more monosaccharides to form a linker tetrasaccharide. This is a branching point for several different GAGs. The resulting CS polymer is transformed into CS/DS by epimerization of GlcA (orange) to IdoA (green) by DS-epi1, followed by sulfation (blue). b) The biosynthesis can also be initiated by XylNap, forming soluble GAGs.

GAG chains are classified according to their carbohydrate composition and degree of sulfation. For example, chondroitin sulfate/dermatan sulfate (CS/DS) consists of repeating units of *N*-acetylgalactosamine (GalNAc) and uronic acids, i.e., glucuronic acid (GlcA) or iduronic acid (IdoA), in various proportions.

CS/DS chains are present on at least 32 PGs, many of which are implicated in diseases^1^ and syndromes,^2,3^ as well as in both malignant and normal cell development.^4^ A notable group of metabolic disorders, collectively termed mucopolysaccharidoses (MPS), arises from mutations in key enzymes required for the degradation of excess GAGs, leading to abnormal accumulation. The clinical outcome of MPS varies with disease severity but typically includes cardiac abnormalities, short stature, intellectual disability, and markedly reduced lifespan. Among the different MPS subtypes, Maroteaux–Lamy syndrome (MPS VI) is caused by a deficiency of the enzyme arylsulfatase B, which is essential for CS/DS catabolism.^5^ Consequently, individuals with MPS VI accumulate CS/DS and often do not survive into adulthood. Current treatments, such as enzyme replacement therapy with galsulfase (Naglazyme),^6^ can alleviate some symptoms but do not halt disease progression and are associated with extremely high costs.^7^ In musculocontractural Ehlers–Danlos syndrome the patient has a mutation either in Chst4 gene or the Dse gene disrupting the dermatan sulfate biosynthesis, resulting in congenital malformations of extracellular matrix. No treatment for this syndrome presently available. Therefore, there is a pressing need for new therapeutic approaches for these disorders.

The biosynthesis of glycosaminoglycan (GAG) chains is initiated by the xylosylation of a serine residue within the proteoglycan (PG) core protein (Figure 1a). The resulting xylosylated protein is subsequently galactosylated by two distinct galactosyltransferases and then glucuronylated, yielding a linker tetrasaccharide. This linker serves as the primer for polymerization into full-length GAG chains. Postsynthetic modifications of chondroitin sulfate (CS) include the epimerization of GlcA to IdoA, as well as sulfation, thereby converting CS into a heterogeneous mixture of CS and DS. Full length GAG chains typically comprise 50–200 carbohydrate residues. Notably, GAG biosynthesis can also be initiated by xylosides bearing hydrophobic aglycones, such as 2-naphthyl β-d-xylopyranoside (XylNap; Figure 1b). These xylosides act as acceptors for the initial galactosylation catalyzed by β4GalT7, thus providing a simplified model system for studying GAG biosynthesis (*vide infra*).

The key enzyme, responsible for CS to CS/DS interconversion, is dermatan sulfate epimerase 1 (DS-epi1), which catalyzes the epimerization of GlcA into IdoA. DS-epi1 binds to the growing GAG chain and initiates processive epimerization toward the nonreducing end, thereby converting CS into CS/DS hybrid regions.^8^ IdoA is thermodynamically less stable than GlcA. However, DS-epi1 forms a complex with the sulfotransferase D4ST1,^9^ which sulfates the newly formed IdoA residues. This sulfation stabilizes IdoA and promotes the formation of extended blocks of sulfated DS.

Given its critical role in CS/DS formation, DS-epi1 represents a promising target for future drug development. Recent structural studies have elucidated the architecture of its active site, revealing a deep groove capable of accommodating intact GAG chains.^10^ Epimerization is catalyzed by His205, Tyr473, and Tyr261, with His205 initiating the reaction by deprotonating the carboxylic acid group. The process proceeds through two transition states and an intermediate enol (Figure 2b).

**Figure 2:**
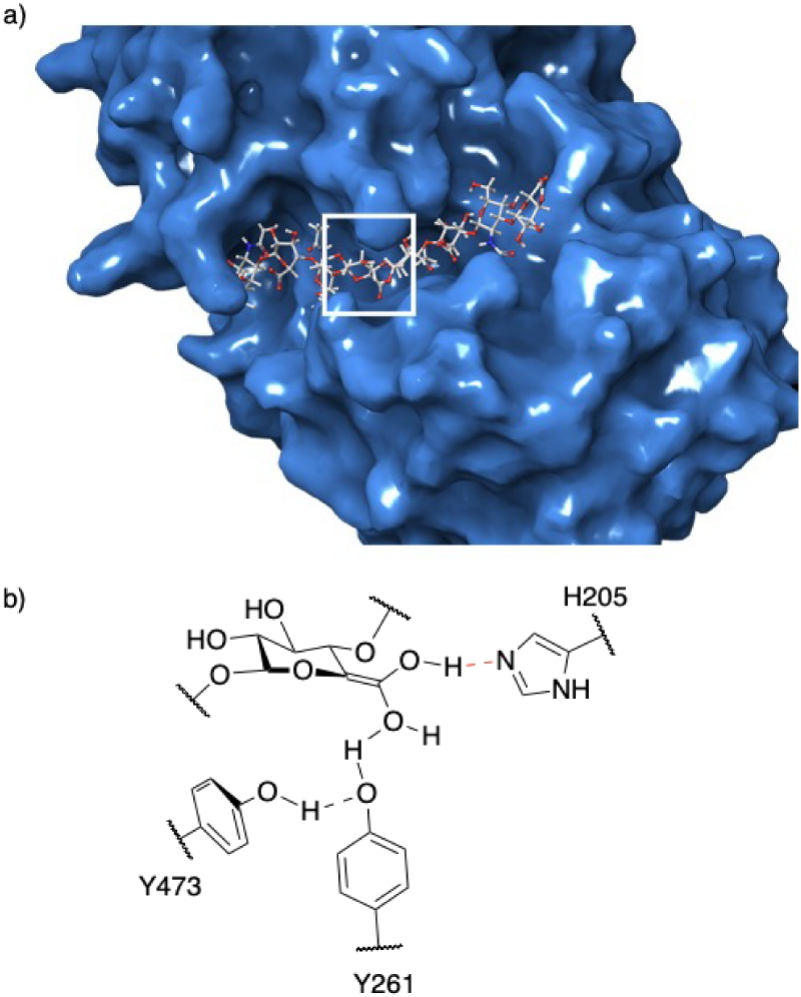
a) An octasaccharide docked in the cleft of DS-epi1. The white box indicates the active site. The reducing end of the octasaccharide is to the left. b) The intermediate enol is stabilized by tyrosine 261 and 473, and histidine 205.

In 2024, Maccarana et al. reported the first inhibitors of DS-epi1.^11^ Virtual screening, using the structurally related enzyme chondroitinase AC as a model, was employed to assemble a chemical library of 1,064 compounds, which were subsequently assayed for DS-epi1 inhibition. Seventeen compounds exhibited inhibitory activity at 10 μM, and two of these were further characterized, showing IC_50_ values of 12–16 μM. All identified inhibitors contained a carboxylic acid moiety, which we hypothesize mimic the GlcA present in the GAG chain (Figure 3a).

**Figure 3:**
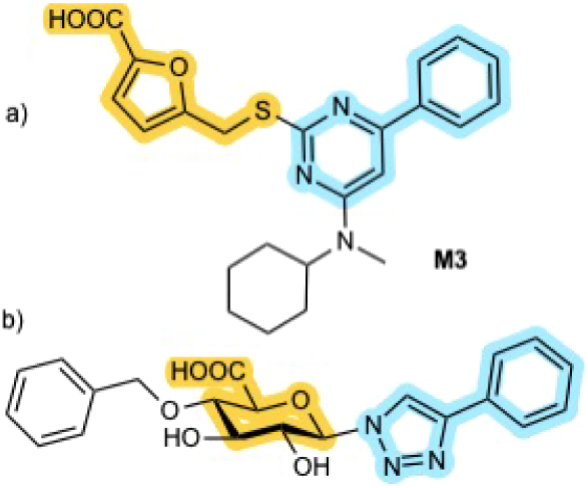
a) Inhibitor (M3) identified by Maccarana et al. b) Proposed inhibitor (**1**) designed from GlcA. Key design elements are shown in color.

Within the DS-epi1 binding cleft, the galactosamine at position 4 of the epimerizing GlcA, interacts hydrophobically with Trp98, in a manner reminiscent of galactose binding in galectins.^12^ From a design perspective, this interaction is absent in the inhibitors identified by Maccarana et al.

In the present study, we introduce DS-epi1 inhibitors based on the natural GlcA scaffold (Figure 3b, Chart [Fig chart1]). These compounds were designed with the native ligand in mind, aiming to mimic the repeating unit of CS. GlcA serves as the core building block, with derivatization at positions 1 and 4, selected to most closely reproduce the overall shape and key interaction features of CS, including the hydrophobic interaction with Trp98.

**1 chart1:**
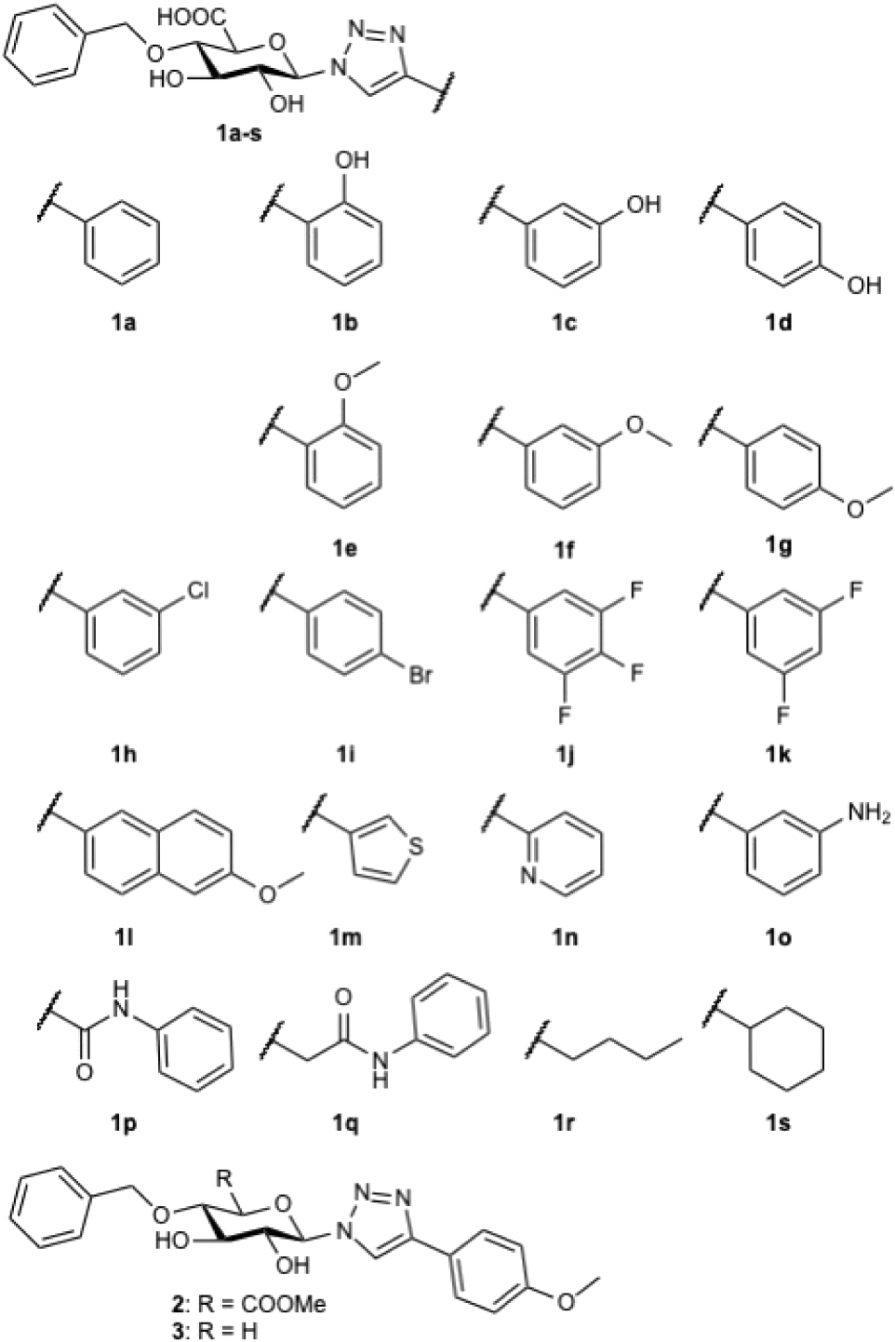
Structures of Investigated GlcA Derivatives

## Results and Discussion

### Synthesis of GlcA Derivatives

There are several synthetic strategies for GlcA analogs. While it is possible to use GlcA as starting material, it is usually more convenient to start from a glucose derivative and perform a selective oxidation of position 6 as the very last step. GlcA is more expensive than glucose and the carboxylic acid moiety can interfere with many reagents and reactions. Furthermore, selective TEMPO oxidation of primary alcohols enables easy conversion of glucose to GlcA.^13,14^

The synthesis of targets **1a**–**s** proceeded through mostly conventional carbohydrate synthetic methods. Benzylidene protection of methyl α-d-glucose, followed by reductive opening with CoCl_2_/BH_3_·THF^15^ gave selectively the 4-*O*-benzyl derivative **4** (Scheme 1). The demethylation of the anomeric position was then performed as a mild tandem acetylation/deacetylation,^16^ using acetic anhydride/acetic acid/sulfuric acid, followed by potassium carbonate in water to give compound **6** in 54% over two steps.

**Scheme 1:**
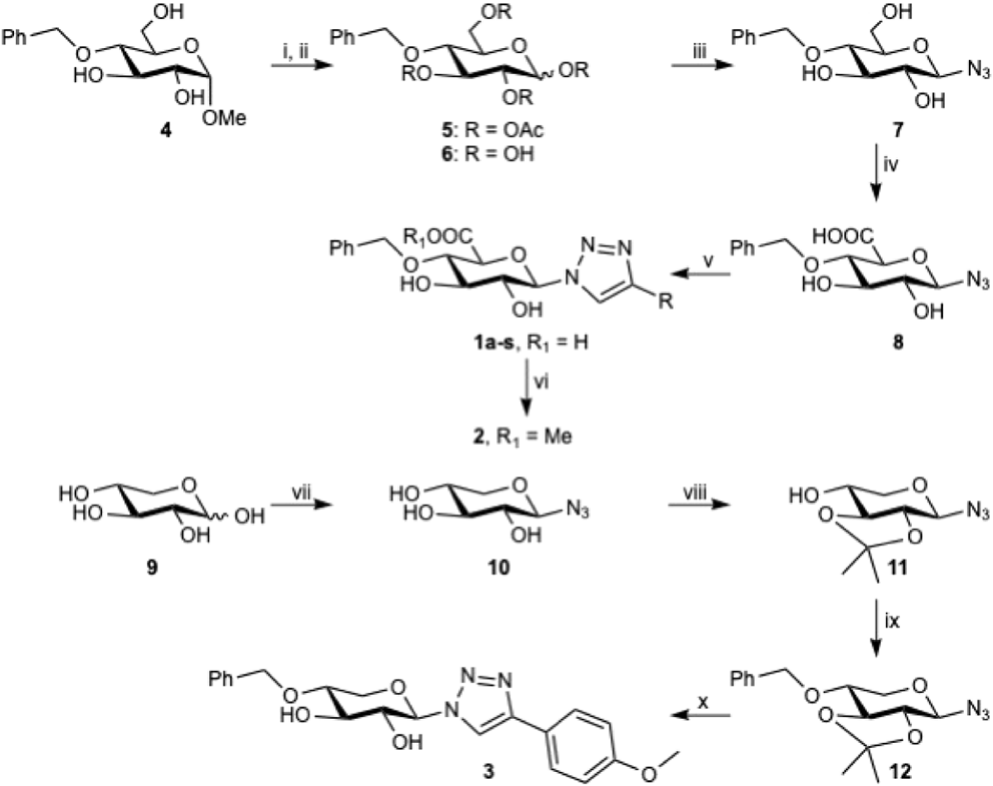
Reagents and Conditions: (i) Ac_2_O, H_2_SO_4_ (conc.), AcOH, 8 h, r.t.; (ii) K_2_CO_3_ (s), MeOH, H_2_O, 2 h, r.t., 54% over 2 steps; (iii) DMC, NaN_3_, TEA, H_2_O, 3 h, 0–20 °C, 95%; (iv) TEMPO, NaOCl, KBr, NaHCO_3_ (aq. sat), H_2_O, 0 °C, Overnight, 87%; (v) CuSO_4_, NaAsc, Alkyne, H_2_O, THF, r.t., 1–16 h, 10-82%; (vi) MeOH, MS3Å, Amberlite IR 120 H^+^, r.t., 62%; (vii) DMC, NaN_3_, TEA, H_2_O, 3 h, 0–20 °C, 99%; (viii) 2-Methoxypropene, CSA, DMF, 48 h, r.t., 62%; (ix) BnBr, NaH, THF, r.t., 48%; (x) 1. HCl (aq, 1 M) 2. CuSO_4_, NaAsc, Alkyne, H_2_O, THF, Overnight, r.t., 29%

Instead of the traditional α-halogenation, followed by displacement with azide, we used a one-pot method,^17^ i.e., 2-chloro-1,3-dimethylimidazolinium chloride (DMC), sodium azide, and triethylamine in water, which furnished compound **7** in an excellent 95% yield. TEMPO oxidation, with sodium hydroxide to adjust pH to 11.2–11.7, which is optimal for oxidation of glucose derivatives,^18^ initially proved to be difficult. While the oxidation worked well, simultaneous chlorination of the benzyl ether significantly lowered the yield of compound **8**. When the reaction instead was performed in sodium hydrogen carbonate buffer (pH 8.5), no chlorination was observed and compound **8** was isolated in 87% yield. In the divergent last step, different groups were coupled by standard click reactions. As our compounds are rather hydrophilic, we opted to use copper sulfate and sodium ascorbate dissolved in a 1:1 mix of THF and water, along with the alkyne, which yielded compounds **1a**–**s** in copper-catalyzed azide–alkyne cycloadditions, as seen in Scheme 1.

To explore the importance of the carboxylic acid moiety, we synthesized the analogous xylose derivative **3**, as well as the methyl ester-analog **2**. To reach the methyl ester-analog, compound **1g** was subjected to Fischer esterification conditions with Amberlite IR120 H^+^ resin in MeOH overnight.

Compound **10** was synthesized from xylose using the DMC procedure in almost quantitative yield and then protected as the 2,3-acetonide **11**. Nucleophilic substitution using benzyl bromide with NaH gave compound **12** in 48%. Deprotection under acidic conditions followed by click reaction gave compound **3** in 29%.

### Epimerization Inhibition of DS-epi1

We have previously developed an assay for measuring DS-epi1 inhibition,^19^ based on the ability of the enzyme to abstract and release the H4-proton of GlcA. By using ^3^H-labeled GlcA in the CS substrate, ^3^H_2_O is released during epimerization of the substrate and the radioactivity is measured, after distillation, versus a negative control without inhibitor or enzyme.^20,21^ As seen in Figure 4, most of the compounds served as inhibitors. Interestingly, the free phenols, i.e., **1b**–**d** were poor inhibitors in comparison to the analogous methoxylated derivatives **1e**–**g**. The *p*-methoxyphenol **1g** showed the highest activity, i.e., 97% inhibition at 1 mM. In contrary, compounds **1n**–**q**, with nitrogen containing groups, showed low inhibition, possible due to chelation of manganese.^22^ Neither the methyl ester **2**, nor the xylose derivative **3**, served as inhibitors.

**Figure 4:**
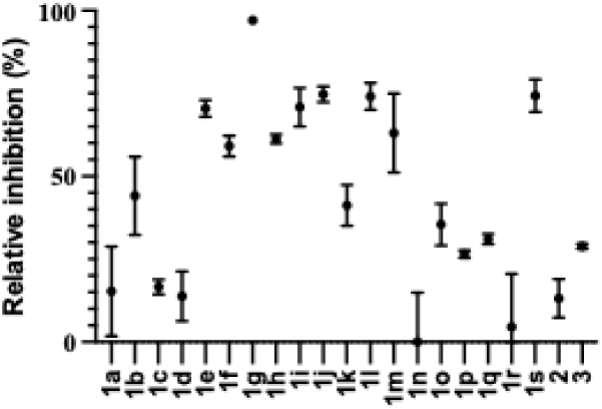
Inhibition of DS-epi1. 1.0 mM inhibitor **1a**–**s**, incubated with 5-[^3^H]­chondroitin, and DS-epi1. The decrease of formed tritiated water in the presence of inhibitor is expressed in % of the amount tritiated water formed without added inhibitor.

To investigate the inhibitory potential of compound **1g**, we subjected it to a concentration-dependent assay (Figure 5) which gave an IC_50_ of 42 ± 4 μM.

**Figure 5:**
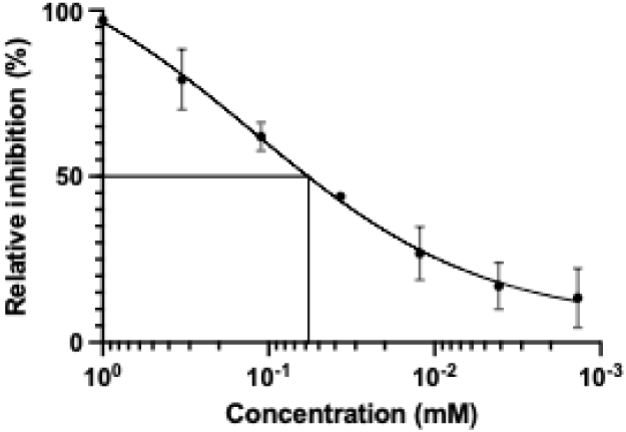
Relative inhibition plotted versus the concentration of **1g**. The IC_50_ of **1g** is 42 ± 4 μM.

### Attempted Cocrystallization of DS-Epi1 with **1g**

We have previously determined the apo structure of the catalytic domains of DS-epi1 at 2.4 Å resolution.^10^ To elucidate the structure of DS-epi1 in complex with **1g**, we conducted both cocrystallization and soaking experiments using a range of commercial crystallization screens, including BCS, JCSG+, MIDAS, and SG1 (Molecular Dimensions). Crystals formed under approximately 30 different conditions; however, the majority were either too fragile to manipulate or failed to diffract beyond 10 Å. Among these, only the BCS screen produced crystals with diffraction better than 5 Å. The most promising crystals (0.2 M lithium sulfate, 0.1 M HEPES pH 7.2, 25% v/v PEG Smear Broad), diffracted to ∼3.5 Å. Despite this, the resulting electron density map revealed only a diffuse region of increased density in the active site of DS-epi1, lacking discernible structural features.

### Docking Studies of **1g**

MD simulations were performed with compound **1g** placed in the active site of DS-epi1 to elucidate the binding mechanism of the ligand. However, **1g** did not seem to be entirely complementary to the protein, and repeatedly lost its binding within 10 ns of MD. Therefore, a new MD was performed with the intention to achieve an induced fit. For this purpose, a light harmonic constraint was introduced on the carboxylate carbon of **1g**, biasing it to be placed at the same location as the corresponding carboxylate group of the natural ligand. A 10 ns MD resulted in a new binding pose with pi stacking between His205 and the benzyloxy ring of **1g**. Starting from this pose, the complex was stable during a 100 ns MD simulation (RMSF for the carbohydrate part of the molecule was 1.5 Å, Supporting Information). As seen in Figure 6, compound **1g** bind, as hypothesized, to the active site of DS-epi1, effectively blocking the natural ligand from binding to the cleft. Starting from the binding pose of **1g**, a 100 ns MD simulation was performed for compound **1i**. The resulting trajectory closely resembled that of **1g**, but with a lower ligand RMSF. However, the π-stacking interaction between His205 and the benzyloxy ring observed for **1g** was only partially retained.

**Figure 6:**
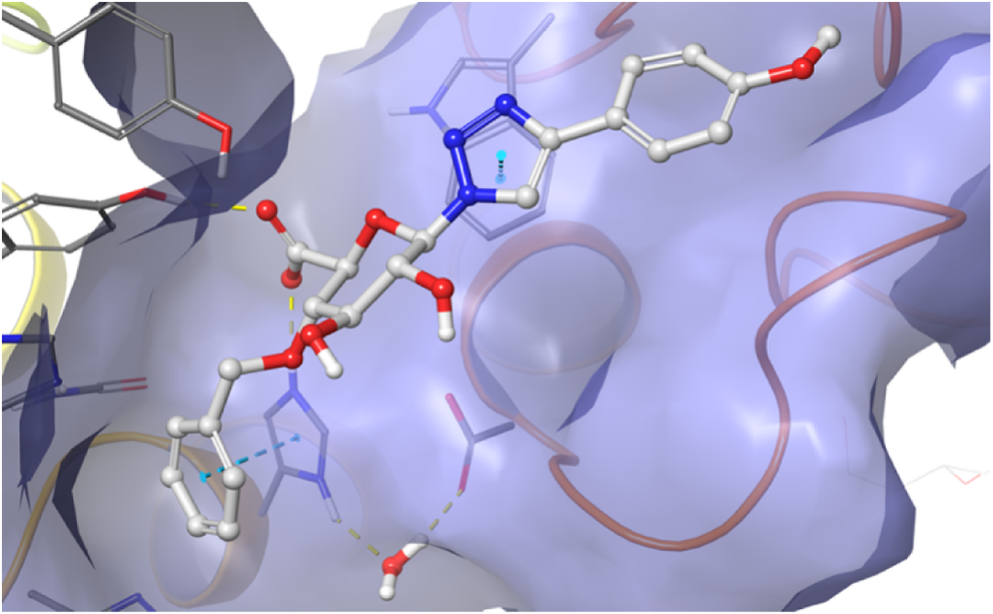
Result of a trajectory cluster of the 100 ns MD of **1g**. Hydrogen bonds are displayed in yellow and aromatic stacking between the benzyloxy substituent and His205 and between the triazole ring and Trp98 are shown with blue dashes.

MD simulations were also performed on Maccarana’s substance 3 (**M3**). No stable binding was found in the active site, probably because **M3** cannot pi stack with His205. When **M3** was placed in a similar manner to Figure 3C of Maccarana’s paper, but rotated, i.e., interchanged placement of the phenyl and the cyclohexyl rings, a stable binding was found. The chain with the carboxylate thus moved to interact with either the oxyanion hole of the backbone of Gly361 and Gln362, with Lys360 or with Arg97 (Figure 7).

**Figure 7:**
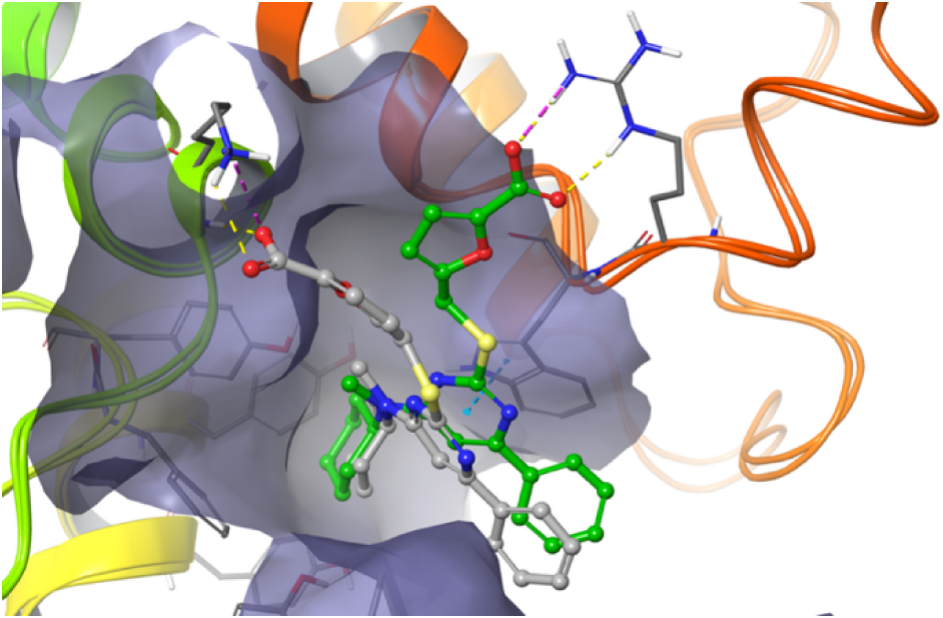
Two entries (green and gray) from the trajectory clusters of M3 indicates how the cyclohexyl ring is stationary in a pocket of six aromatic residues. There is intermittent pi stacking, shown as blue dashes, between Trp98 and the pyrimidine ring of M3, as well as hydrogen bonds in yellow to the carboxylate group.

### Disaccharide Analysis

The biological effect of compound **1g** (100 μM) was evaluated by incubation with HFL1 fibroblast cultures with the polysaccharide acceptor 2-naphthyl β-d-xylopyranoside (**XylNap**).^23^ The CS/DS produced was analyzed in a disaccharide analysis after chondroitinase ABC and chondroitinase B degradation, AMAC derivatization, and separation using reverse phase HPLC. Unfortunately, no decrease of IdoA content was observed. We believe that this is due to poor uptake of the rather hydrophilic compound.

To address this, we prepared and tested the methyl ester analog **2**, which was then assessed in the same assay. The methyl ester makes the compound less hydrophilic, and it is reasonable that it is cleaved off in cells by nonspecific esterases.^24^ No decrease of formation of IdoA was observed but treatment of compound **2** (100 μM) resulted in 40% decrease of total formation of soluble GAGs (Supporting Information, Figure S2). Furthermore, cells treated with 200 μM of compound **2**, resulted in cell toxicity (Supporting Information, Figure S1).

## Conclusions

In our ongoing project aiming at inhibition of dermatan sulfate epimerase 1 (DS-epi1), we have synthesized a collection of potential inhibitors based on 1,4-disubstituted glucuronic acids. These compounds were synthesized from glucose through a divergent approach, where different groups were introduced in the last step using conventional click chemistry, yielding 19 derivatives that were tested in a functional *in vitro* assay. The most potent compound, **1g**, carrying a *p*-methoxy-phenyl group, exhibited an IC_50_ of 42 ± 4 μM. Interestingly, neither the analogous methyl ester **2**, nor the xylose derivative **3**, showed significant inhibition, which emphasize the importance of the carboxylic acid moiety for strong binding to DS-epi1.

Despite several attempts, we were not successful in cocrystallization of **1g** with DS-epi1. Instead, computational MD simulations revealed a strong binding of **1g**, close to the active site of the enzyme, over 100 ns. Interestingly, our MD simulations indicated that compound **1g** bind in a similar manner as GlcA, while the inhibitor presented by Maccarana bind further away from the active site, and thus mimic a GalNAc residue. This opens for design of chimeras with potentially strong binding (Figure 8).

**Figure 8:**
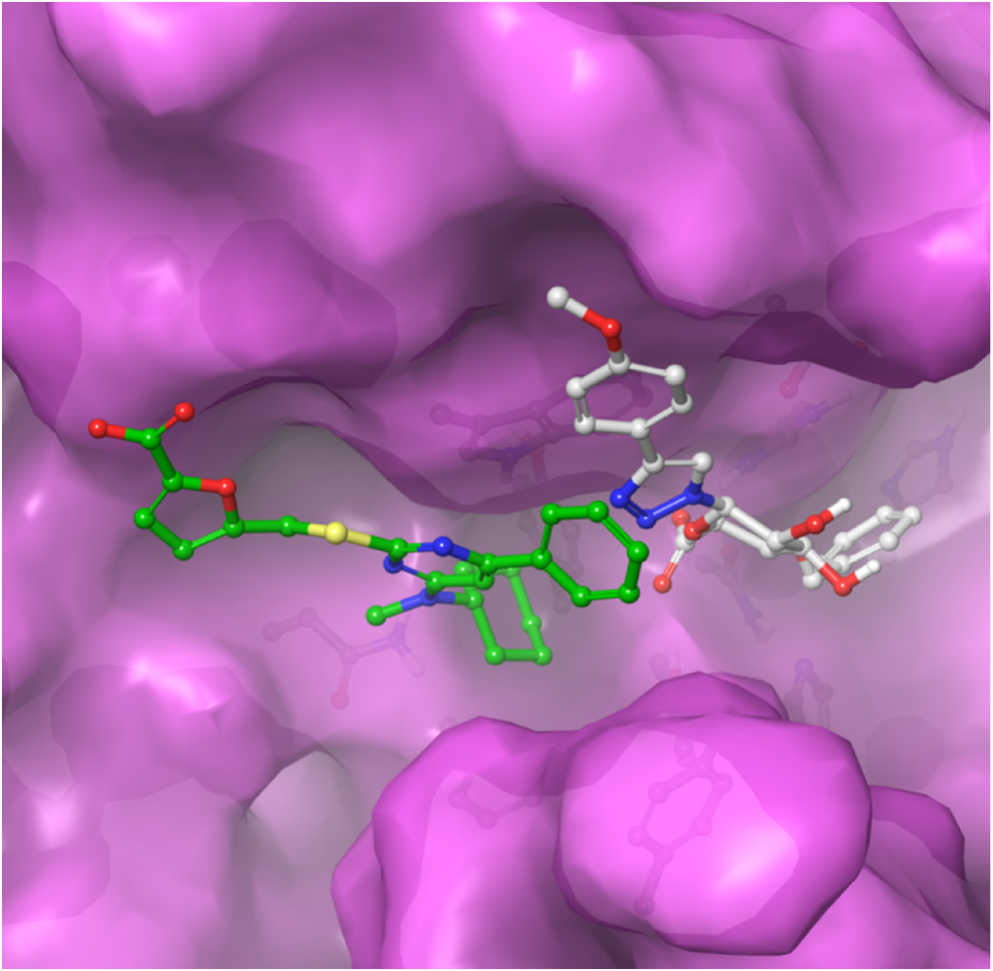
Overlapped calculated binding poses of compound 1g (gray) and compound M3 (green).

Even though neither compound **1g**, nor the methylated analog **2**, served as inhibitors in cell studies, probably due to poor cellular uptake, the IC_50_ in combination with MD simulations, indicate that these 1,4-disubstituted glucuronic acid derivatives are promising leads in the search for DS-epi1 inhibition.

## Experimental Section

### Synthesis

All moisture and air-sensitive reactions were carried out under an atmosphere of argon, using oven-dried glassware. All solvents where either purchased dry under 3 Å molecular sieves or dried, using MBRAUN SPS-800 Solvent purification system unless otherwise stated. Purchased reagents were used without further purification. Thin-layer chromatography was performed on precoated aluminum plates with silica gel 60 F254 0.25 mm (Merck). Spots where visualized with UV light or by charring with ethanolic anisaldehyde or ethanolic sulfuric acid solution. Magnesium sulfate was used to dry combined organic phases. Preparative chromatography was performed on Biotage Isolera One flash system, using Biotage Sfär Silica HC D 20 μM or manually on Matrex silica gel (25–70 μm). Final compounds where purified by reverse phase chromatography on an Agilent Technologies 1260 Infinity HPLC with Waters Xselect C18 column, 5 μm, 19 × 250 mm. Optical rotations were measured on a Bellingham and Stanley model ADP450 polarimeter where [α]*^T^*_D_ (*c* = g/100 mL), D indicate the sodium D line (589 nm) and *T* indicates the temperature. NMR-spectra were reported at ambient pressure and temperature on a Bruker Avance II at 400 MHz (^1^H) and 100 MHz (^13^C) or a Bruker Ascend at 500 MHz (^1^H) and 125 MHz (^13^C) and assigned using either only 1D (^1^H and ^13^C)-spectra or a combination of 2D (COSY, HMCQ, HMBC) and 1D-spectra. Chemical shifts are reported in ppm, with reference to residual solvent peaks (δ_H_ CHCl_3_ = 7.26 ppm, CD_3_OH = 3.31 ppm, C_6_D_5_H = 7.16 ppm) and solvent signals (δ_C_ CDCl_3_ = 77.0 ppm, CD_3_OD = 49.0 ppm, C_6_D_6_ = 128.06 ppm). Coupling constant values are given in Hz. Mass spectra were recorded on Waters XEVO G2 (Positive ESI).

### General Procedure for the Copper­(I)-Catalyzed Azide–Alkyne Cycloaddition

The azidosugar **8** or **12** (1.0 equiv) was dissolved in THF:H_2_O (1:1) along with suitable alkyne (1.2 equiv). Ascorbic acid sodium salt (1.5 equiv) was added along with CuSO_4_ (1.0 equiv). Reaction was stirred at r.t. until monitored by completion by LC–MS or TLC. Solvent was evaporated and the crude was purified by preparative HPLC (H_2_O/MeCN + 0.1% FA 90:10 → 0:100) and aqueous NH_3_ added (until basic) to yield the ammonium salt of compound **1a**–**s** and **3**. The solution was then lyophilized to give the final compound.

#### 4-Phenyl-1-(4-*O*-benzyl-β-d-glucopyranosyluronic acid)-[1,2,3]­triazole (**1a**)

**8** (11 mg, 0.036 mmol, 1 equiv) was subjected to general click-procedure to yield **1a** as a white amorphous solid (1.6 mg, 0.0039 mmol, 11%). [α]^25^_D_ +27.2 (*c* 0.42, MeOH). ^1^H NMR (400 MHz, DMSO) δ 8.89 (s, 1H, C=CH–N), 7.94–7.87 (m, 2H, Ar–H), 7.52–7.43 (m, 2H, Ar–H), 7.43–7.24 (m, 6H, Ar–H), 5.82 (d, *J* = 9.3 Hz, 1H), 5.78 (d, *J* = 5.4 Hz, 1H, −OH-2), 5.69 (d, *J* = 5.8 Hz, 1H, −OH-3), 4.93 (d, *J* = 11.1 Hz, 1H, −CH_2_−), 4.61 (d, *J* = 11.2 Hz, 1H, −CH_2_−), 4.26 (d, *J* = 9.8 Hz, 1H, H-5), 3.96 (td, *J* = 9.0, 5.6 Hz, 1H, H-2), 3.76 (td, *J* = 8.9, 5.1 Hz, 1H, H-3), 3.65 (t, *J* = 9.4 Hz, 1H, H-4). ^13^C NMR (101 MHz, DMSO) δ 170.02, 146.97, 138.97, 130.96, 129.41, 128.55, 128.51, 128.01, 127.88, 125.69, 121.02, 87.60, 79.78, 76.46, 74.24, 72.48. HRMS calcd for C_21_H_21_N_3_O_6_ + H^+^ (M + H)^+^: 412.1509, found: 412.1506.

#### 4-(2-Hydroxy-phenyl)-1-(4-*O*-benzyl-β-d-glucopyranosyluronic acid)-[1,2,3]­triazole (**1b**)

**8** (10 mg, 0.032 mmol, 1 equiv) was subjected to general click-procedure to yield **1b** as a white amorphous solid (2.0 mg, 0.0047 mmol, 15%). [α]^25^_D_ +55.0 (*c* 0.48, MeOH). ^1^H NMR (500 MHz, MeOD_4_) δ 8.67 (s, 1H, C=CH–N), 7.91 (dd, *J* = 7.7, 1.7 Hz, 1H, Ar–H), 7.47–7.42 (m, 2H, Ar–H), 7.33–7.27 (m, 2H, Ar–H), 7.26–7.21 (m, 1H, Ar–H), 7.18 (ddd, *J* = 8.6, 7.3, 1.7 Hz, 1H, Ar–H), 6.96–6.89 (m, 2H, Ar–H), 5.67 (d, *J* = 9.2 Hz, 1H, H-1), 4.82 (d, *J* = 2.0 Hz, 2H, −CH_2_−), 4.05–3.98 (m, 2H, H-5, H-2), 3.79 (t, *J* = 9.2 Hz, 1H, H-3), 3.74 (t, *J* = 9.0 Hz, 1H, H-4). ^13^C NMR (126 MHz, MeOD_4_) δ 173.91, 154.51, 144.73, 138.61, 128.77, 127.85, 127.55, 126.95, 126.35, 121.21, 119.15, 115.96, 115.67, 88.27, 80.21, 79.88, 76.67, 74.16, 72.86. HRMS calcd for C_21_H_21_N_3_O_7+_ Na^+^ (M + Na)^+^: 450.1277, found: 450.1269.

#### 4-(3-Hydroxy-phenyl)-1-(4-*O*-benzyl-β-d-glucopyranosyluronic acid)-[1,2,3]­triazole (**1c**)

**8** (10 mg, 0.032 mmol, 1 equiv) was subjected to general click-procedure to yield **1c** as a white amorphous solid (1.5 mg, 0.0035 mmol, 11%). [α]^25^_D_ +33.1 (*c* 0.18, MeOH). ^1^H NMR (500 MHz, MeOD_4_) δ 8.55 (s, 1H, C=CH–N), 7.42 (d, *J* = 7.5 Hz, 2H, Ar–H), 7.37–7.24 (m, 5H, Ar–H), 6.84–6.78 (m, 1H, Ar–H), 5.72 (d, *J* = 9.2 Hz, 1H, H-1), 4.92 (d, *J* = 11.2 Hz, 1H, −CH_2_−), 4.77 (d, *J* = 10.8 Hz, 1H, −CH_2_−), 4.21–4.14 (m, 1H, H-5), 4.05 (t, *J* = 7.6 Hz, 1H, H-2), 3.85–3.78 (m, 2H, H-3, H-4). ^13^C NMR (126 MHz, MeOD_4_) δ 157.63, 147.60, 138.25, 131.27, 129.58, 127.75, 127.66, 127.13, 119.70, 116.56, 114.97, 112.01, 88.02, 79.48, 77.62, 76.64, 74.32, 72.52. HRMS calcd for C_21_H_21_N_3_O_7+_ Na^+^ (M + Na)^+^: 450.1277, found: 450.1274.

#### 4-(4-Hydroxy-phenyl)-1-(4-*O*-benzyl-β-d-glucopyranosyluronic acid)-[1,2,3]­triazole (**1d**)

**8** (10 mg, 0.032 mmol, 1 equiv) was subjected to general click-procedure to yield **1d** as a white amorphous solid (11 mg, 0.016 mmol, 51%, tetrabutylammonium salt).[α]^25^_D_ + 58.0 (*c* 0.36, MeOH). ^1^H NMR (500 MHz, MeOD_4_) δ 8.47 (s, 1H, C=CH–N), 7.70–7.63 (m, 2H, Ar–H), 7.47–7.42 (m, 2H, Ar–H), 7.33–7.26 (m, 2H, Ar–H), 7.26–7.20 (m, 1H, Ar–H), 6.89–6.81 (m, 2H, Ar–H), 5.62 (d, *J* = 9.2 Hz, 1H, H-1), 4.81 (s, 2H, −CH_2_−), 3.99 (d, *J* = 9.2 Hz, 1H, H-5), 3.96 (t, *J* = 9.0 Hz, 1H, H-2), 3.77 (t, *J* = 9.2 Hz, 1H, H-3), 3.73 (t, *J* = 8.9 Hz, 1H, H-4), 3.28–3.20 (m, 8H, −CH_2_−), 1.66 (ddd, *J* = 12.0, 10.1, 6.2 Hz, 8H, −CH_2_−), 1.42 (h, *J* = 7.4 Hz, 8H, −CH_2_−), 1.03 (t, *J* = 7.4 Hz, 11H, −CH_3_). ^13^C NMR (126 MHz, MeOD_4_) δ 173.99, 157.53, 147.73, 138.60, 127.88, 127.54, 126.94, 126.68, 121.56, 118.35, 115.19, 88.24, 80.18, 79.88, 76.59, 74.10, 72.89, 58.03, 29.27, 23.29, 19.22, 12.43. HRMS calcd for C_21_H_21_N_3_O_7+_ Na^+^ (M + Na)^+^: 450.1277, found: 450.1270.

#### 4-(2-Methoxy-phenyl)-1-(4-*O*-benzyl-β-d-glucopyranosyluronic acid)-[1,2,3]­triazole **(1e)**

**8** (10 mg, 0.032 mmol, 1 equiv) was subjected to general click-procedure to yield **1e** as a white amorphous solid (1.4 mg, 0.0032 mmol, 10%) [α]^25^_D_ +17.1 (*c* 0.53, MeOH). ^1^H NMR (400 MHz, MeOD) δ 8.59 (s, 1H, C=CH–N), 8.11 (dd, *J* = 7.7, 1.7 Hz, 1H, Ar–H), 7.47–7.40 (m, 2H, Ar–H), 7.39–7.20 (m, 4H, Ar–H), 7.11 (d, *J* = 7.4 Hz, 1H, Ar–H), 7.05 (td, *J* = 7.5, 1.0 Hz, 1H, Ar–H), 5.68 (d, *J* = 9.2 Hz, 1H, H-1), 4.82 (d, *J* = 7.2 Hz, 2H, −CH_2_−), 4.04 (t, *J* = 8.9 Hz, 2H, H-2, H-5), 3.98 (s, 3H, −CH_3_), 3.84–3.70 (m, 2H, H-3, H-4). ^13^C NMR (101 MHz, MeOD) δ 156.11, 138.47, 129.10, 127.87, 127.72, 127.16, 126.80, 122.75, 120.36, 118.63, 110.86, 88.13, 79.85, 76.85, 74.35, 72.60, 54.50. HRMS calcd for C_22_H_23_N_3_O_7_ + H^+^ (M + H)^+^: 442.1614, found: 442.1619.

#### 4-(3-Methoxy-phenyl)-1-(4-*O*-benzyl-β-d-glucopyranosyluronic acid)-[1,2,3]­triazole (**1f**)

**8** (20 mg, 0.065 mmol, 1 equiv) was subjected to general click-procedure to yield **1f** as a white amorphous solid (17 mg, 0.0039 mmol, 82%). [α]^25^_D_ +13.4 (*c* 0.80, MeOH). ^1^H NMR (500 MHz, MeOD) δ 8.66 (s, 1H, C=CH–N), 7.49–7.41 (m, 4H, Ar–H), 7.41–7.22 (m, 4H, Ar–H), 6.94 (ddd, *J* = 8.2, 2.6, 1.0 Hz, 1H, Ar–H), 5.68 (d, *J* = 9.2 Hz, 1H, H-1), 4.84 (d, *J* = 2.5 Hz, 2H, −CH_2_−), 4.07–3.96 (m, 2H, H-5, H-2), 3.88 (s, 3H, CH_3_), 3.85–3.72 (m, 2H, H-4, H-3). ^13^C NMR (126 MHz, MeOD) δ 160.19, 147.41, 138.57, 131.43, 129.57, 127.86, 127.56, 126.97, 119.77, 117.60, 113.74, 110.42, 88.25, 80.12, 76.58, 74.12, 72.89, 54.26. HRMS calcd for C_22_H_23_N_3_O_7_ + H^+^ (M + H)^+^: 442.1614, found: 442.1618.

#### 4-(4-Methoxy-phenyl)-1-(4-*O*-benzyl-β-d-glucopyranosyluronic acid)-[1,2,3]­triazole (**1g**)

**8** (30 mg, 0.097 mmol, 1 equiv) was subjected to general click-procedure to yield **1g** as a white amorphous solid (35 mg, 0.0088 mmol, 82%). [α]^25^_D_ +10.1 (*c* 0.46, MeOH). ^1^H NMR (500 MHz, MeOD) δ 8.53 (s, 1H, C=CH–N), 7.83–7.75 (m, 2H, Ar–H), 7.50–7.44 (m, 2H, Ar–H), 7.36–7.29 (m, 2H, Ar–H), 7.28–7.23 (m, 1H, Ar–H), 7.09–6.99 (m, 2H, Ar–H), 5.66 (d, *J* = 9.2 Hz, 1H, H-1), 4.83 (s, 2H, −CH_2_−), 4.06–3.95 (m, 2H, H-5, H-2), 3.85 (s, 3H, −CH_3_), 3.83–3.72 (m, 2H, H-3, H-4). ^13^C NMR (126 MHz, MeOD) δ 161.39, 148.94, 140.08, 129.37, 129.05, 128.45, 128.10, 124.23, 120.16, 115.37, 89.75, 81.65, 78.11, 75.61, 74.38, 55.77. HRMS calcd for C_22_H_23_N_3_O_7_ + H^+^ (M + H)^+^: 442.1614, found: 442.1606.

#### 4-(3-Chloro-phenyl)-1-(4-*O*-benzyl-β-d-glucopyranosyluronic acid)-[1,2,3]­triazole (**1h**)

**8** (21 mg, 0.069 mmol, 1 equiv) was subjected to general click-procedure to yield **1h** as a white amorphous solid (23 mg, 0.052 mmol, 75%). [α]^25^_D_ +17.2 (*c* 0.24, MeOH). ^1^H NMR (500 MHz, MeOD) δ 8.65 (s, 1H, C=CH–N), 7.91 (t, *J* = 1.9 Hz, 1H, Ar–H), 7.79 (dt, *J* = 7.8, 1.3 Hz, 1H, Ar–H), 7.44 (t, *J* = 7.9 Hz, 1H, Ar–H), 7.40–7.35 (m, 3H, Ar–H), 7.34–7.30 (m, 2H, Ar–H), 7.29–7.24 (m, 1H, Ar–H), 5.75 (d, *J* = 9.3 Hz, 1H, H-1), 4.95 (d, *J* = 10.7 Hz, 1H, −CH_2_−), 4.72 (d, *J* = 10.7 Hz, 1H, −CH_2_−), 4.23 (d, *J* = 9.3 Hz, 1H, H-5), 4.06 (t, *J* = 9.1 Hz, 1H, H-2), 3.83 (t, *J* = 8.7 Hz, 1H, H-3), 3.79 (t, *J* = 9.0 Hz, 1H, H-4). ^13^C NMR (126 MHz, MeOD) δ 170.13, 146.16, 138.08, 134.50, 132.13, 130.11, 127.84, 127.72, 127.68, 127.23, 125.11, 123.57, 120.44, 87.93, 79.13, 76.64, 76.53, 74.43, 72.35. HRMS calcd for C_21_H_20_N_3_O_6_Cl + H^+^ (M + H)^+^: 446.1119, found: 446.1114.

#### 4-(4-Bromo-phenyl)-1-(4-*O*-benzyl-β-d-glucopyranosyluronic acid)-[1,2,3]­triazole (**1i**)

**8** (21 mg, 0.069 mmol, 1 equiv) was subjected to general click-procedure to yield **1i** as a white amorphous solid (20 mg, 0.051 mmol, 74%). [α]^25^_D_ −5.75 (*c* 0.24, MeOH). ^1^H NMR (500 MHz, MeOD) δ 8.64 (s, 1H, C=CH–N), 7.81 (dt, *J* = 8.3, 1.9 Hz, 2H, Ar–H), 7.63 (dt, *J* = 8.7, 2.3 Hz, 2H, Ar–H), 7.42–7.37 (m, 2H, Ar–H), 7.36–7.32 (m, 2H, Ar–H), 7.31–7.26 (m, 1H, Ar–H), 5.76 (d, *J* = 9.3 Hz, 1H, H-1), 4.96 (d, *J* = 10.7 Hz, 1H, −CH_2_−), 4.74 (d, *J* = 10.8 Hz, 1H, −CH_2_−), 4.24 (d, *J* = 9.5 Hz, 1H, H-5), 4.07 (t, *J* = 9.1 Hz, 1H, H-2), 3.83 (m, 2H, H-3, H-4). ^13^C NMR (126 MHz, MeOD) δ 171.72, 147.96, 139.59, 133.17, 130.80, 129.21, 129.18, 128.72, 128.49, 123.17, 121.57, 89.43, 80.66, 78.14, 75.92, 73.88. HRMS calcd for C_21_H_20_N_3_O_6_Br + H^+^ (M + H)^+^: 490.0614, found: 490.0605.

#### 4-(3,4,5-Trifluoro-phenyl)-1-(4-*O*-benzyl-β-d-glucopyranosyluronic acid)-[1,2,3]­triazole (**1j**)

**8** (20 mg, 0.064 mmol, 1 equiv) was subjected to general click-procedure to yield **1j** as a white amorphous solid (9.2 mg, 0.020 mmol, 31%). [α]^25^_D_ +12.0 (*c* 1.15, MeOH). ^1^H NMR (500 MHz, MeOD) δ 8.69 (s, 1H, C=CH–N), 7.72–7.62 (m, 2H, Ar–H), 7.44–7.38 (m, 2H, Ar–H), 7.36–7.31 (m, 2H, Ar–H), 7.30–7.25 (m, 1H, Ar–H), 5.74 (d, *J* = 9.3 Hz, 1H, H-1), 4.93 (d, *J* = 10.7 Hz, 1H, −CH_2_−), 4.76 (d, *J* = 10.7 Hz, 1H, −CH_2_−), 4.23–4.15 (m, 1H, H-5), 4.08–3.98 (m, 1H, H-2), 3.86–3.77 (m, 2H, H-3, H-4). ^13^C NMR (126 MHz, MeOD) δ 152.32 (ddd, *J* = 248.6, 9.7, 3.5 Hz), 144.75, 138.21, 127.73, 127.67, 127.15, 120.71, 109.59–109.34 (m), 88.03, 79.40, 76.59, 74.34, 72.53. HRMS calcd for C_21_H_18_N_3_O_6_F_3_ + H^+^ (M + H)^+^: 466.1226, found: 466.1225.

#### 4-(3,5-Difluoro-phenyl)-1-(4-*O*-benzyl-β-d-glucopyranosyluronic acid)-[1,2,3]­triazole (**1k**)

**8** (12 mg, 0.040 mmol, 1 equiv) was subjected to general click-procedure to yield **1k** as a white amorphous solid (2.8 mg, 0.0063 mmol, 16%). [α]^25^_D_ +9.8 (*c* 0.52, MeOH). ^1^H NMR (500 MHz, MeOD) δ 8.75 (s, 1H, C=CH–N), 7.56–7.48 (m, 2H, Ar–H), 7.48–7.44 (m, 2H, Ar–H), 7.35–7.28 (m, 2H, Ar–H), 7.29–7.23 (m, 1H, Ar–H), 6.96 (tt, *J* = 9.1, 2.4 Hz, 1H, Ar–H), 5.69 (d, *J* = 9.2 Hz, 1H, H-1), 4.84 (d, *J* = 3.8 Hz, 2H, −CH_2_−), 4.04 (d, *J* = 9.0 Hz, 1H, H-5), 3.99 (t, *J* = 8.9 Hz, 1H, H-2), 3.84–3.73 (m, 2H, H-3, H-4). ^13^C NMR (126 MHz, MeOD) δ 164.48 (d, *J* = 13.0 Hz), 162.51 (d, *J* = 13.1 Hz), 145.38 (d, *J* = 3.6 Hz), 138.55, 133.82 (d, *J* = 10.7 Hz), 127.83, 127.56, 126.98, 120.81, 108.09–107.80 (m), 102.65 (t, *J* = 25.9 Hz), 88.24, 80.06, 76.55, 74.11, 72.86. HRMS calcd for C_21_H_19_N_3_O_6_F_2_ + H^+^ (M + H)^+^: 448.1320, found: 448.1316.

#### 4-(6-Methoxy-2-naphthyl)-1-(4-*O*-benzyl-β-d-glucopyranosyluronic acid)-[1,2,3]­triazole (**1l**)

**8** (4.6 mg, 0.015 mmol, 1 equiv) was subjected to general click-procedure to yield **1l** as a white amorphous solid (4.3 mg, 0.0088 mmol, 59). [α]^25^_D_ +3.4 (*c* 1.1, MeOH). ^1^H NMR (400 MHz, MeOD) δ 8.67 (s, 1H, C=CH–N), 8.27 (s, 1H, Ar–H), 7.91 (dd, *J* = 8.5, 1.7 Hz, 1H, Ar–H), 7.87–7.79 (m, 2H, Ar–H), 7.44–7.39 (m, 2H, Ar–H), 7.36–7.21 (m, 4H, Ar–H), 7.16 (dd, *J* = 8.9, 2.5 Hz, 1H, Ar–H), 5.73 (d, *J* = 9.2 Hz, 1H, H-1), 4.90 (d, *J* = 10.7 Hz, 1H, −CH_2_−), 4.78 (d, *J* = 10.7 Hz, 1H, −CH_2_−), 4.17–4.12 (m, 1H, H-5), 4.10–4.02 (m, 1H, H-2), 3.93 (s, 3H, −CH_3_), 3.83–3.79 (m, 2H, H-3, H-4). ^13^C NMR (101 MHz, MeOD) δ 158.24, 147.85, 138.43, 134.68, 129.27, 129.02, 127.87, 127.73, 127.23, 127.19, 125.34, 123.95, 123.83, 119.68, 119.00, 105.42, 88.21, 79.76, 78.28, 76.74, 74.37, 72.75, 54.40. HRMS calcd for C_26_H_25_N_3_O_7_ + H^+^ (M + H)^+^: 492.1771, found: 492.1767.

#### 4-(Thiophen-3-yl)-1-(4-*O*-benzyl-β-d-glucopyranosyluronic acid)-[1,2,3]­triazole (**1m**)

**8** (4.6 mg, 0.015 mmol, 1 equiv) was subjected to general click-procedure to yield **1m** as a white amorphous solid (2.6 mg, 0.0062 mmol, 42%). [α]^25^_D_ −4.5 (*c* 0.20, MeOH). ^1^H NMR (400 MHz, MeOD) δ 8.50 (s, 1H, C=CH–N), 7.79 (t, *J* = 2.1 Hz, 1H, Ar–H), 7.51 (d, *J* = 1.8 Hz, 2H, Ar–H), 7.45–7.39 (m, 2H, Ar–H), 7.34–7.28 (m, 2H, Ar–H), 7.28–7.22 (m, 1H, Ar–H), 5.68 (d, *J* = 9.2 Hz, 1H, H-1), 4.89 (d, *J* = 5.6 Hz, 1H, −CH_2_−), 4.77 (d, *J* = 10.7 Hz, 1H, −CH_2_−), 4.13–4.07 (m, 1H, H-5), 4.03–3.96 (m, 1H, H-2), 3.82–3.74 (m, 2H, H-4, H-3). ^13^C NMR (101 MHz, MeOD) δ 143.84, 138.47, 131.33, 127.87, 127.71, 127.15, 126.23, 125.37, 120.97, 119.50, 88.18, 79.81, 78.53, 76.70, 74.33, 72.76. HRMS calcd for C_19_H_19_N_3_O_6_S + H^+^ (M + H)^+^: 418.1073, found: 418.1071.

#### 4-(2-Pyridyl)-1-(4-*O*-benzyl-β-d-glucopyranosyluronic acid)-[1,2,3]­triazole (**1n**)

**8 (**10 mg, 0.032 mmol, 1 equiv) was subjected to general click-procedure to yield **1n** as a white amorphous solid (5.6 mg, 0.014 mmol, 43). [α]^25^_D_ +6.7 (*c* 1.05, MeOH). ^1^H NMR (500 MHz, DMSO) δ 13.22 (s, 1H, −COOH), 8.82 (s, 1H, C=CH–N), 8.67–8.60 (m, 1H, Ar–H), 8.06 (dt, *J* = 7.9, 1.1 Hz, 1H, Ar–H), 7.92 (td, *J* = 7.7, 1.8 Hz, 1H, Ar–H), 7.37 (ddd, *J* = 7.5, 4.9, 1.2 Hz, 1H, Ar–H), 7.33 (d, *J* = 4.4 Hz, 4H, Ar–H), 7.31–7.25 (m, 1H, Ar–H), 5.82 (d, *J* = 9.3 Hz, 1H, H-1), 5.77–5.65 (m, 2H, −OH-2/3), 4.91 (d, *J* = 11.1 Hz, 1H, −CH_2_−), 4.60 (d, *J* = 11.1 Hz, 1H, −CH_2_−), 4.19 (d, *J* = 9.5 Hz, 1H, H-5), 4.06–3.98 (m, 1H, H-2), 3.72 (t, *J* = 8.9 Hz, 1H, H-3), 3.66 (t, *J* = 9.3 Hz, 1H, H-4). ^13^C NMR (126 MHz, DMSO) δ 169.96, 150.09, 150.06, 147.67, 138.95, 137.65, 128.44, 127.93, 127.76, 123.55, 122.99, 120.03, 87.61, 79.69, 76.46, 74.13, 72.15. HRMS calcd for C_20_H_20_N_4_O_6_ + H^+^ (M + H)^+^: 413.1461, found: 413.1462.

#### 4-(3-Amino-phenyl)-1-(4-*O*-benzyl-β-d-glucopyranosyluronic acid)-[1,2,3]­triazole (**1o**)

**8** (26 mg, 0.085 mmol, 1 equiv) was subjected to general click-procedure to yield **1o** as a white amorphous solid (16 mg, 0.038 mmol, 45%). [α]^25^_D_ +9.7 (*c* 0.93, MeOH). ^1^H NMR (500 MHz, MeOD) δ 8.54 (s, 1H), 7.42–7.25 (m, 8H), 6.90–6.85 (m, 1H), 5.76 (d, *J* = 9.3 Hz, 1H), 4.96 (d, *J* = 10.8 Hz, 1H), 4.74 (d, *J* = 10.7 Hz, 1H), 4.25 (d, *J* = 8.8 Hz, 1H), 4.08 (t, *J* = 8.8 Hz, 1H), 3.88–3.78 (m, 2H). ^13^C NMR (126 MHz, MeOD) δ 170.21, 147.54, 144.42, 138.08, 130.99, 129.48, 127.72, 127.68, 127.23, 119.85, 117.41, 116.67, 113.60, 87.91, 79.16, 76.67, 74.43, 72.35. HRMS calcd for C_21_H_21_N_4_O_6_ + H^+^ (M + H)^+^: 427.1618, found: 427.1615.

#### 4-(Phenylcarbamoyl)-1-(4-*O*-benzyl-β-d-glucopyranosyluronic acid)-[1,2,3]­triazole (**1p**)

**8** (20 mg, 0.064 mmol, 1 equiv) was subjected to general click-procedure to yield **1p** as a white amorphous solid (15 mg, 0.032 mmol, 50%). ^1^H NMR (500 MHz, MeOD_4_) δ 8.80 (s, 1H, −C=CH–N), 7.75–7.69 (m, 2H, Ar–H), 7.46–7.41 (m, 2H, Ar–H), 7.39–7.34 (m, 2H, Ar–H), 7.33–7.27 (m, 2H, Ar–H), 7.27–7.21 (m, 1H, Ar–H), 7.15 (t, *J* = 7.4 Hz, 1H), 5.72 (d, *J* = 9.2 Hz, 1H), 4.83–4.78 (m, 2H), 4.03 (d, *J* = 9.1 Hz, 1H), 4.00 (t, *J* = 9.0 Hz, 1H), 3.81–3.71 (m, 2H). ^13^C NMR (126 MHz, MeOD_4_) δ 173.48, 158.87, 142.95, 138.52, 137.68, 128.39, 127.84, 127.57, 126.99, 125.50, 124.21, 120.41, 88.21, 80.03, 79.56, 76.56, 74.19, 72.77. HRMS calcd for C_22_H_22_N_4_O_7_ + H^+^ (M + H)^+^: 455.1567, found: 455.1560.

#### 4-(2-Oxo-2-(phenylamino)­ethyl)-1-(4-*O*-benzyl-β-d-glucopyranosyluronic acid)-[1,2,3]­triazole (**1q**)

**8** (20 mg, 0.064 mmol, 1 equiv) was subjected to general click-procedure to yield **1q** as a white amorphous solid (11 mg, 0.027 mmol, 42). [α]^25^_D_ +7.5 (*c* 0.83, MeOH). ^1^H NMR (400 MHz, MeOD) δ 8.21 (s, 1H, C=CH–N), 7.60–7.53 (m, 2H, Ar–H), 7.45–7.38 (m, 2H, Ar–H), 7.34–7.27 (m, 4H, Ar–H), 7.27–7.21 (m, 1H, Ar–H), 7.14–7.05 (m, 1H, Ar–H), 5.63 (d, *J* = 9.2 Hz, 1H, H-1), 4.82 (d, *J* = 9.1 Hz, 1H, −CH_2_−), 4.77 (d, *J* = 10.7 Hz, 1H, −CH_2_−), 4.06–4.02 (m, 1H, H-5), 3.99–3.90 (m, 1H, H-2), 3.88 (s, 2H, −CH_2_−), 3.79–3.70 (m, 2H, H-3, H-4). ^13^C NMR (101 MHz, MeOD) δ 137.00, 126.88, 126.37, 126.16, 125.60, 122.43, 120.98, 118.37, 86.73, 78.40, 75.03, 72.78, 71.33, 31.65. HRMS calcd for C_23_H_24_N_4_O_7+_ H^+^ (M + H)^+^: 442.1614, found: 442.1619.

#### 4-(1-Butyl)-1-(4-*O*-benzyl-β-d-glucopyranosyluronic acid)-[1,2,3]­triazole (**1r**)

**8** (10 mg, 0.032 mmol, 1 equiv) was subjected to general click-procedure to yield **1r** as a white amorphous solid (2.0 mg, 0.0051 mmol, 16%). [α]^25^_D_ −6.3 (*c* 0.96, MeOH). ^1^H NMR (400 MHz, MeOD) δ 8.59 (s, 1H, C=CH–N), 8.11 (dd, *J* = 7.7, 1.7 Hz, 1H, Ar–H), 7.48–7.40 (m, 2H, Ar–H), 7.39–7.20 (m, 4H, Ar–H), 7.11 (d, *J* = 1.1 Hz, 1H, Ar–H), 7.05 (td, *J* = 7.5, 1.0 Hz, 1H, Ar–H), 5.68 (d, *J* = 9.2 Hz, 1H, H-1), 4.82 (d, *J* = 7.1 Hz, 2H, −CH_2_−), 4.04 (t, *J* = 8.9 Hz, 2H, H-5, H-2), 3.98 (s, 3H, CH_3_), 3.84–3.70 (m, 2H, H-4, H-3). ^13^C NMR (101 MHz, MeOD) δ 156.11, 138.47, 129.10, 127.87, 127.72, 127.16, 126.80, 122.75, 120.36, 118.63, 110.86, 88.13, 79.85, 76.85, 74.35, 72.60, 54.50. HRMS calcd for C_19_H_25_N_3_O_6_ + H^+^ (M + H)^+^: 392.1822, found: 392.1823.

#### 4-Cyclohexyl-1-(4-*O*-benzyl-β-d-glucopyranosyluronic acid)-[1,2,3]­triazole (**1s**)

**8** (10 mg, 0.032 mmol, 1 equiv) was subjected to general click-procedure to yield **1s** as a white amorphous solid (2.0 mg, 0.0048 mmol, 15). [α]^25^_D_ +4.9 (*c* 0.41, MeOH). ^1^H NMR (400 MHz, MeOD) δ 8.00 (s, 1H, C=CH–N), 7.46–7.39 (m, 2H, Ar–H), 7.36–7.22 (m, 3H, Ar–H), 5.61 (d, *J* = 9.3 Hz, 1H, H-1), 4.91 (d, *J* = 10.9 Hz, 1H, −CH_2_−), 4.77 (d, *J* = 10.7 Hz, 1H, −CH_2_−), 4.08 (d, *J* = 8.5 Hz, 1H, H-5), 4.02–3.93 (m, 1H, H-2), 3.81–3.70 (m, 2H, H-3, H-4), 2.82–2.72 (m, 1H, Cyclo-H), 2.13–2.00 (m, 2H, Cyclo-H), 1.91–1.81 (m, 2H, Cyclo-H), 1.81–1.73 (m, 1H, Cyclo-H), 1.56–1.39 (m, 4H, Cyclo-H), 1.40–1.28 (m, 1H, Cyclo-H). ^13^C NMR (101 MHz, MeOD) δ 138.46, 127.86, 127.70, 127.14, 119.42, 88.02, 79.79, 76.78, 74.32, 72.58, 35.15, 32.60, 25.83, 25.70. HRMS calcd for C_21_H_27_N_3_O_6_ + Na^+^ (M + Na)^+^: 440.1790, found: 440.1798.

#### 4-(4-Methoxy-phenyl)-1-(methyl 4-*O*-benzyl-β-d-glucopyranosyluronate)-[1,2,3]­triazole **(2)**

**1g** (4.0 mg, 0.095 mmol, 1 equiv) was dissolved in MeOH (5 mL, dry) along with 3 Å molecular sieves and Amberlite IR 120 H^+^ resin. Solution was stirred overnight before resin and sieves were filtered off. Solvent was evaporated *in vacuo* and the crude was purified by preparative HPLC (H_2_O/MeCN + 0.1% FA 90:10 → 0:100). The solvent was freeze-dried to yield **2** as a white amorphous solid (2.7 mg, 0.0059 mmol, 62%). [α]^25^_D_ −6.25 (*c* 0.96, DCM).

^1^H NMR (500 MHz, MeOD) δ 8.45 (s, 1H, C=CH–N), 7.80–7.73 (m, 2H, Ar–H), 7.33 (d, *J* = 4.1 Hz, 4H, Ar–H), 7.31–7.26 (m, 1H, Ar–H), 7.03–6.98 (m, 2H, Ar–H), 5.72 (d, *J* = 9.3 Hz, 1H, H-1), 4.95 (d, *J* = 11.1 Hz, 1H, −CH_2_−), 4.66 (d, *J* = 11.1 Hz, 1H, −CH_2_−), 4.32–4.24 (m, 1H, H-5), 4.10–4.02 (m, 1H, H-2), 3.83 (d, *J* = 7.3 Hz, 5H, H-3, H-4, −O–CH_3_), 3.69 (s, 3H, −CH_3_). ^13^C NMR (126 MHz, MeOD) δ 168.79, 159.98, 147.52, 138.08, 127.80, 127.62, 127.29, 126.64, 122.52, 118.90, 113.89, 87.85, 78.86, 76.72, 76.38, 74.34, 72.21, 54.28, 51.58. HRMS calcd for C_23_H_25_N_3_O_7_ + H^+^ (M + H)^+^: 454.1614, found: 454.1630.

#### 4-(4-Methoxy-phenyl)-1-(4-*O*-benzyl-β-d-xylopyranosyluronic acid)-[1,2,3]­triazole (**3**)

**12** (24 mg, 0.092 mmol, 1 equiv) was subjected to general click-procedure, monitored via LC–MS for completion. Then treated with 1 M HCl until monitored for completion to yield **2** as a white amorphous solid (11 mg, 0.026 mmol, 29%). [α]^25^_D_ −22.3 (*c* 0.25, acetone). ^1^H NMR (500 MHz, DMSO) δ 8.69 (s, 1H, C=CH–N), 7.83–7.76 (m, 2H, Ar–H), 7.43–7.33 (m, 4H, Ar–H), 7.33–7.26 (m, 1H, Ar–H), 7.07–6.99 (m, 2H, Ar–H), 5.61 (d, *J* = 5.0 Hz, 1H, −OH-2), 5.58 (d, *J* = 6.0 Hz, 1H, −OH-3), 5.54 (d, *J* = 9.2 Hz, 1H, H-1), 4.76 (d, *J* = 12.0 Hz, 1H, −CH_2_−), 4.69 (d, *J* = 12.0 Hz, 1H, −CH_2_−), 4.04 (d, *J* = 6.1 Hz, 1H, H-4), 3.85–3.80 (m, 1H, H-2), 3.79 (s, 3H, −CH_3_), 3.62–3.55 (m, 1H, H-3), 3.52–3.44 (m, 2H, H-5_ax_, H-5_eq_). ^13^C NMR (126 MHz, DMSO) δ 159.44, 146.68, 139.15, 128.57, 128.00, 127.82, 126.90, 123.56, 119.63, 114.72, 88.42, 77.18, 76.47, 72.68, 72.39, 66.32, 55.53. HRMS calcd for C_21_H_23_N_3_O_6_ + H^+^ (M + H)^+^: 398.1716, found: 398.1711.

#### 4-*O*-Benzyl-d-glucopyranose (**6**)

**4** (3.5 g, 12 mmol, 1 equiv) was dissolved in Ac_2_O (25 mL, 0.25 mol, 20 equiv) and acetic acid (20 mL, 0.32 mol, 25 equiv) after which H_2_SO_4_ (conc. five drops) was added. Reaction was stirred at r.t. for 7 h after which the reaction was diluted with DCM (100 mL) and water (100 mL). The reaction was then neutralized with NaHCO_3_ (solid) and the aqueous layer extracted with DCM (2 × 50 mL). The combined organic phases where then washed with water (2 × 50 mL), dried with MgSO_4_ and the solvent evaporated *in vacuo*. The resulting crude was then dissolved in a mixture of MeOH and water (5:1, 100 mL) and stirred at r.t. solid K_2_CO_3_ was added in portions of 200 mg every 15 min for a total addition of 1.20 g (8.7 mmol, 1 equiv). The reaction mixture turned yellow upon addition of K_2_CO_3_ and deemed complete 15 min after the last addition, quenched with Amberlite IR-H^+^-resin and the solvent was evaporated *in vacuo*. The crude was purified by column chromatography (MeOH:DCM 0–30% MeOH) to give compound **6** as a white amorphous solid (1.8 g, 6.7 mmol, 54%). Analysis was in accordance with previously published data.^15^

#### 1-Azido-4-*O*-benzyl-1-deoxy-β-d-glucopyranoside (**7**)

**6** (200 mg, 0.74 mmol, 1 equiv) was dissolved in water (5 mL), along with NaN_3_ (430 mg, 6.7 mmol, 9 equiv) and TEA (930 μL, 6.7 mmol, 9 equiv). The reaction vessel was cooled down to 0 °C and then 2-chloro-1,3-dimethylimidazolinium chloride (380 mg, 2.20 mmol, 3 equiv) was added and the reaction was stirred as it warmed up to r.t. Reaction was complete after 3 h and the solvent was evaporated *in vacuo*, EtOH (8 mL) was added and the solid were filtered off. The solvent was then evaporated *in vacuo* and the crude was purified by column chromatography (DCM:MeOH, 0–7% MeOH + 1% TEA). Compound **7** was furnished as a white amorphous solid (210 mg, 0.700 mmol, 95%). ^1^H NMR (400 MHz, MeOD) δ 7.40–7.23 (m, 5H, Ar–H), 4.95 (d, *J* = 11.0 Hz, 1H, −CH_2_−), 4.65 (d, *J* = 11.0 Hz, 1H, −CH_2_−), 4.50 (d, *J* = 8.7 Hz, 1H, H-1), 3.84 (dd, *J* = 12.0, 1.7 Hz, 1H, −CH_2_−), 3.71–3.65 (m, 1H, H-5), 3.61–3.54 (m, 1H, H-3, −CH_2_−), 3.43–3.39 (m, 2H, H-4), 3.18 (dd, *J* = 9.2, 8.7 Hz, 1H, H-2). ^13^C NMR (101 MHz, MeOD) δ 139.95, 129.29, 129.08, 128.68, 92.03, 79.27, 78.70, 78.53, 75.78, 75.06, 62.18.

#### 1-Azido-4-*O*-benzyl-1-deoxy-β-d-glucopyranuronic Acid (**8**)

**7** (100 mg, 0.34 mmol, 1 equiv) was dissolved in NaHCO_3_ (5 mL, sat. aqueous solution, large excess) along with KBr (12 mg, 0.068 mmol, 0.2 equiv), TEMPO (11 mg, 0.068 mmol, 0.2 equiv) and reaction mixture was cooled to 0 °C. NaOCl (0.68 mL, 11–15% available chlorine, 3 equiv) and let stir at 0 °C for 1.5 h. After monitoring, an additional amount of TEMPO (1.1 mg, 0.0068, 0.1 equiv) and NaOCl (0.23 mL, 1 equiv) were added and the reaction mixture was let to stir overnight. The reaction mixture was neutralized with 1 M HCl and purified by HPLC (H_2_O/MeCN + 0.1% FA 90:10 → 0:100) to yield compound **8** as a yellow tinted sticky oil (92 mg, 0.30 mmol, 87%). ^1^H NMR (400 MHz, MeOD) δ 7.38–7.23 (m, 6H, Ar–H), 4.91 (s, 1H, H-5), 4.66 (d, *J* = 10.7 Hz, 1H, H-5), 4.62 (d, *J* = 8.7 Hz, 1H, H-1), 4.02–3.98 (m, 1H, H-4), 3.64–3.57 (m, 2H, H-3, −CH_2_−), 3.23 (ddd, *J* = 9.0, 6.6, 2.5 Hz, 1H, H-2). ^13^C NMR (101 MHz, MeOD) δ 138.21, 127.77, 127.70, 127.26, 90.76, 79.41, 76.34, 75.94, 74.46, 73.26.

#### 1-Azido-1-deoxy-β-d-xylopyranoside (**10**)

**9 (**500 mg, 3.30 mmol, 1 equiv) was dissolved in water (10 mL) along with TEA (4.6 mL, 33 mmol, 10 equiv) and NaN_3_ (1300 mg, 20 mmol, 6 equiv) at r.t. When the solids were dissolved, the reaction mixture was cooled down to 0 °C with an ice bath, and then DMC (1700 mg, 10 mmol, 3 equiv) was added. The reaction was let to reach r.t. and stirred over the weekend. The solvent was evaporated *in vacuo* after which the solids were washed with ethanol (2 × 10 mL). The resulting crude was then purified by column chromatography (MeOH:DCM, 0–15% gradient). After evaporation of solvents *in vacuo*, compound **10** (560 mg, 3.2 mmol, 96%) was isolated as a yellow syrup. Analysis was in accordance with previously published data.^25^

#### 1-Azido-1-deoxy-2,3-*O*-isopropylidene-β-d-xylopyranoside (**11**)

**10** (660 mg, 3.8 mmol, 1 equiv) was dissolved in DMF (5 mL) along with 3 Å molecular sieves. CSA (140 mg, 0.6 mmol, 0.16 equiv) was added and then 2-methoxypropene was added dropwise (0.98 mL, 10 mmol, 2.7 equiv). The reaction was stirred at r.t. overnight. TEA (0.5 mL) was added and the solvent evaporated *in vacuo* after which the crude was purified by column chromatography (EtOAc:Heptane, 10–60% gradient). After evaporation of solvents, compound **11** was isolated as an amorphous solid (500 mg, 2.4 mmol, 62). [α]^25^_D_ −56.3 (*c* 0.56, MeOH). ^1^H NMR (400 MHz, MeOD) δ 4.82 (d, *J* = 8.5 Hz, 1H, H-1), 4.02 (dd, *J* = 11.6, 5.3 Hz, 1H, H-5_eq_), 3.89 (td, *J* = 9.1, 5.3 Hz, 1H, H-4), 3.53 (t, *J* = 9.3 Hz, 1H, H-3), 3.30–3.17 (m, 2H, H-2, H-5_ax_), 1.43 (s, 3H, −CH_3_), 1.43 (s, 3H, −CH_3_). ^13^C NMR (101 MHz, MeOD) δ 112.47, 89.93, 82.40, 77.48, 70.21, 69.59, 49.64, 49.43, 49.21, 49.00, 48.79, 48.57, 48.36, 27.00, 26.61. HRMS calcd for C_8_H_13_N_3_O_4_+ H^+^ (M + H)^+^: 216.0978, found: 216.0978.

#### 1-Azido-4-*O*-benzyl-1-deoxy-2,3-*O*-isopropylidene-β-d-xylopyranoside (**12**)

**11** (150 mg, 0.68 mmol, 1 equiv) was dissolved in dry THF (10 mL) and then 60% NaH in oil (50 mg, 2.1 mmol, 3 equiv) was added. Benzyl bromide (91 μL, 0.77 mmol, 1.1 equiv) was added and the mixture was stirred for 1 h. Temperature was increased to 40 °C and 1 more equivalent of NaH was added and the reaction was let stir overnight. 1 M HCl (3 mL, aq) was added and the reaction mixture was stirred for 30 min whereafter the solvent was evaporated *in vacuo*. HPLC (H_2_O/MeCN + 0.1% FA 90:10 → 0:100) purification of crude yielded **12** as a clear oil (48%, 88 mg, 0.33 mmol). [α]^25^_D_ −55.0 (*c* 1.43, MeOH). ^1^H NMR (400 MHz, MeOD) δ 7.38–7.26 (m, 5H), 4.90 (d, *J* = 8.4 Hz, 1H), 4.78 (d, *J* = 11.8 Hz, 1H), 4.63 (d, *J* = 11.8 Hz, 1H), 4.13 (dd, *J* = 11.9, 5.2 Hz, 1H), 3.85 (ddd, *J* = 9.0, 8.1, 5.2 Hz, 1H), 3.72 (t, *J* = 9.2 Hz, 1H), 3.43 (dd, *J* = 11.9, 8.1 Hz, 1H), 3.28 (dd, *J* = 9.3, 8.4 Hz, 1H), 1.47 (s, 3H), 1.47 (s, 3H). ^13^C NMR (101 MHz, MeOD) δ 136.55, 126.45, 125.96, 125.86, 109.93, 87.05, 79.29, 74.62, 73.90, 70.09, 65.18, 46.72, 46.50, 46.29, 46.08, 45.87, 45.65, 45.44, 24.10, 23.81. HRMS calcd for C_12_H_15_N_3_O_4_+ H^+^ (M + H)^+^: 264.09788, found: 264.1236.

### Enzymatic Incubations of DS-epi1

Inhibitor (1.0 mM) was preincubated with DS-epi1 (1 μg, final concentration 110 nM) for 5 min in a total volume of 100 μL MES buffer (20 mM, pH 5.6) supplemented with MnCl_2_ (10 mM) and 10 μg BSA. [5-^3^H]­chondroitin (∼1 μg, 25,000 dpm) was added, and after incubation for 1 h at 37 °C, the samples were boiled and subsequently centrifuged at 20,000 × *g* for 5 min. The supernatant contained both the unmodified tritiated polysaccharide substrate and tritiated water, formed as the coproduct of epimerase activity, which was isolated by distillation and measured with liquid scintillation counting. Inhibition (%) was plotted against inhibitor concentration (mM), and a logarithmic trendline was fitted to the data. The resulting equation was used to solve for the concentration corresponding to 50% inhibition, providing an approximate IC_50_ value.

### Molecular Dynamics Simulations

Molecular dynamics simulations were performed with the Desmond MD simulation software package in the Schrödinger release 2025-3, using the OPLS-4 force field and the TIP4P2005 water model. A light harmonic constraint (1 kcal mol^–1^ Å^–2^) was applied to all stranded and helix backbone atoms. To find typical binding poses from the MD, clustering of the trajectories was performed with an advanced method that picks exemplar frames from the trajectory.^26^

### Cell Assays

To study the effect of the inhibitors, human fetal lung fibroblasts from ATCC (HFL1) were incubated with 50 μM **XylNap** together with compound **1g** or the methylated analog **2**. The cells were preincubated with 50 to 200 μM inhibitor. After 1 h **XylNap**. The incubation was terminated after 48 h. The GAG was isolated from the medium using ion-exchange chromatography. The GAGs were depolymerized using chondroitinase ABC or chondroitinase B, derivatized using AMAC and finally separated on HPLC on a reversed phase column.^23^

## Supplementary Material



## Notes

Cell microscopy pictures of HFL cells, HPLC chromatogram of AMAC-conjugated disaccharides, ^1^H and ^13^C NMR spectra for all compounds (PDF)

## #

Umeå University, Chemistry. Umeå, SE 901 87, Sweden
