# Supplementary material for: Inhibition of Dermatan Sulfate Epimerase 1 by Substituted Glucuronic Acids
Source: ACS Omega. 2026 Feb 27;11(9):14719–28. doi: 10.1021/acsomega.5c10686 (PMC12980235; doi:10.1021/acsomega.5c10686)
Supplement: Supplementary file 1 [file ao5c10686_si_001.pdf]

Supporting Information

Inhibition of dermatan sulfate epimerase 1 by substituted glucuronic acids

Roberto Mastio,[a] Isolde Zuleta Sjögren,[a] John Dahlquist,[a] Anders Sundin,[a] Gunilla Westergren-Thorsson,[b] Sophie Manner,[a] Emil Tykesson,[b] Anders Malmström,[b] Ulf Ellervik[a,b†]

[a] *Lund University, Department of Chemistry, Centre for Analysis and Synthesis, P.O. Box 124, SE-221 00 Lund, Sweden.*
[b] *Lund University, Department of Experimental Medical Science, P.O. Box 117, SE-221 00 Lund, Sweden.*

† Corresponding author. *E-mail address:* ulf.ellervik@chem.lu.se.

Cell microscopy pictures **...4**

HPLC-chromatograms **...3**

^{1}H- and ^{13}C-NMR **...4-28**

Docking studies **...29-30**

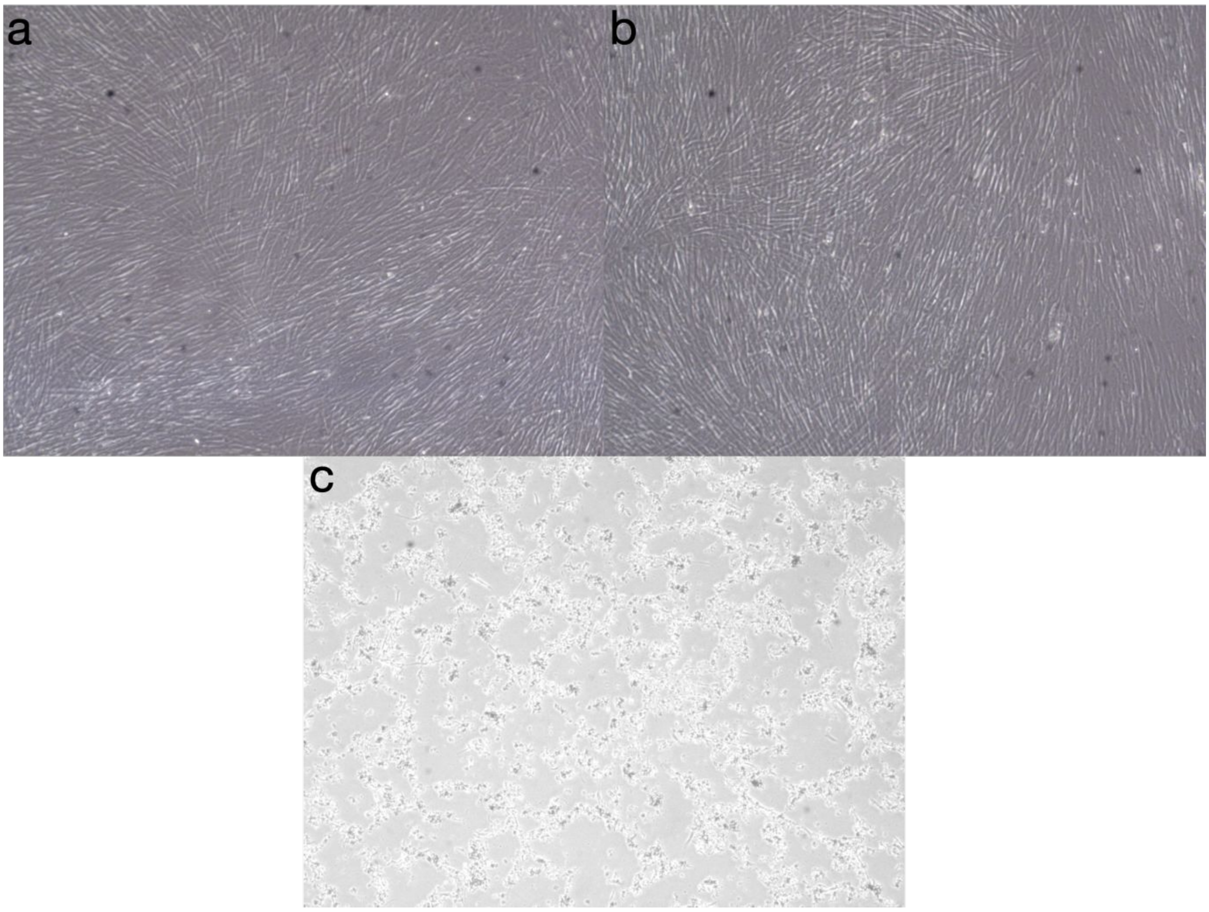

Figure S1: a) HFL-1 cells treated only with 50 μM XylNap. b) HFL-1 cells treated with 50 μM XylNap and 50 μM **2**. c) HFL-1 cells treated with 50 μM XylNap and 200 μM **2**.

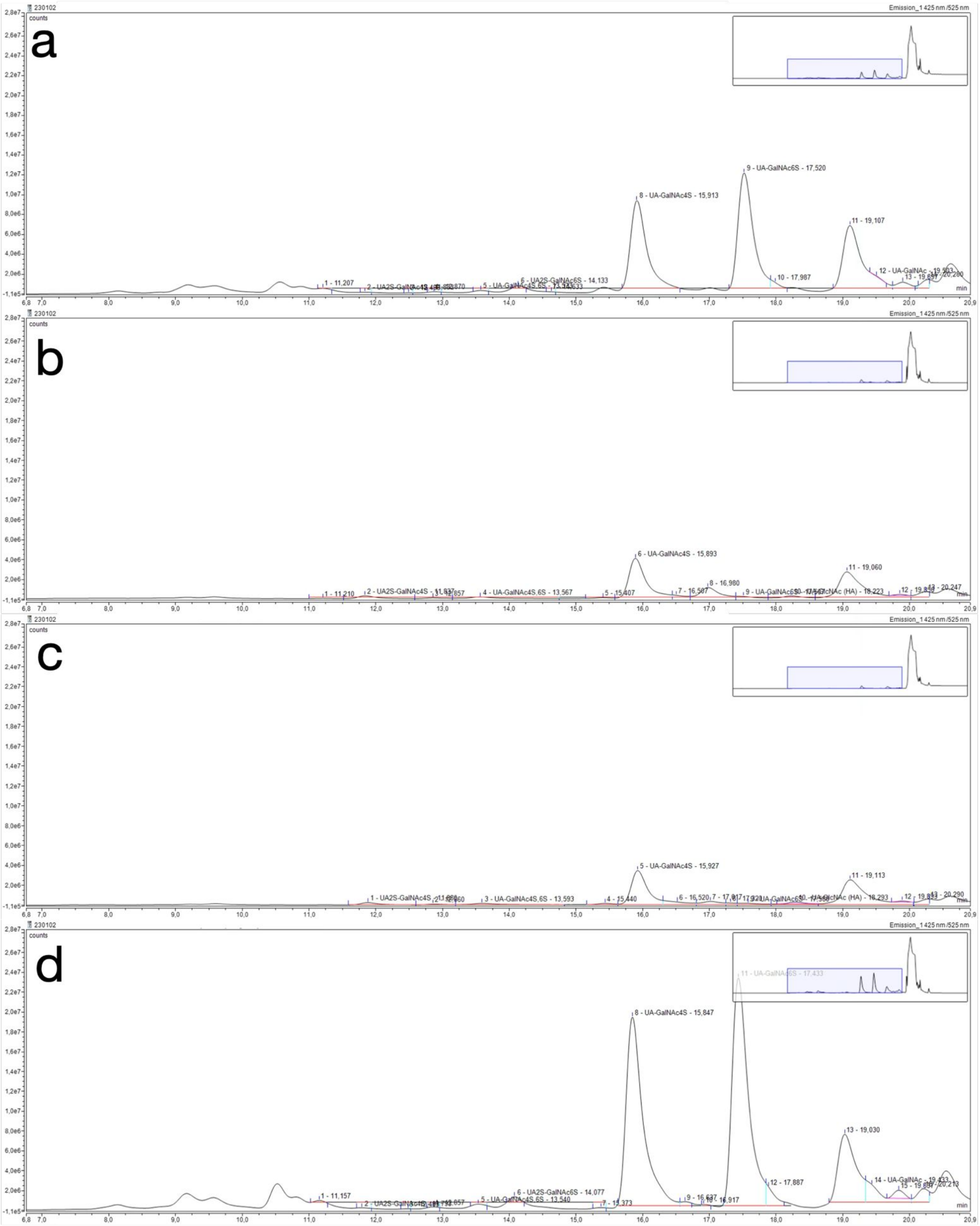

Figure S2: HPLC chromatogram of AMAC-conjugated disaccharides. a) HFL-1 cells treated with 50 μM XylNap, 50 μM **2**, and degraded with Chondroitinase ABC. b) HFL-1 cells treated with 50 μM XylNap and degraded with Chondroitinase B. c) HFL-1 cells treated with 50 μM XylNap, 50 μM **2**, and degraded with Chondroitinase B. d) HFL-1 cells treated with 50 μM XylNap and degraded with Chondroitinase ABC.

¹H and ¹³C spectra of compound 1a

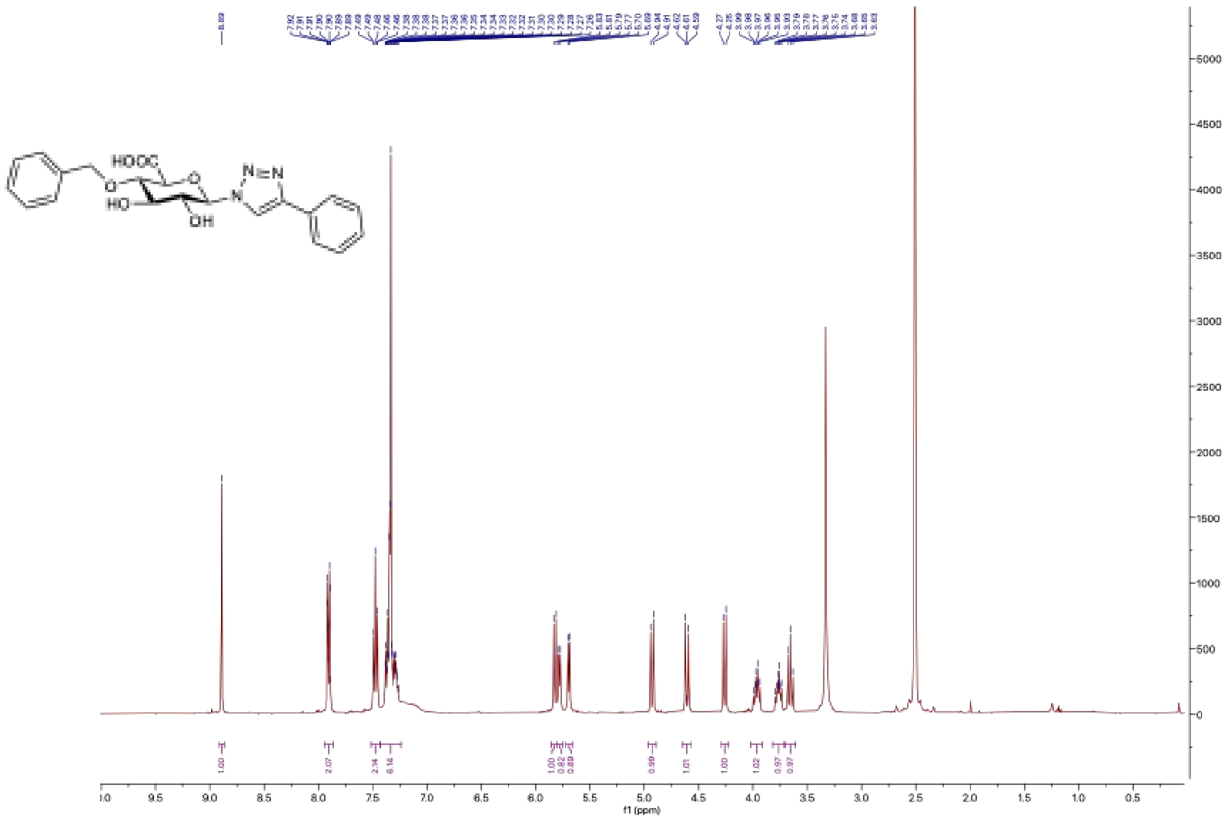

Figure S3: ^{1}H-NMR of compound **1a**

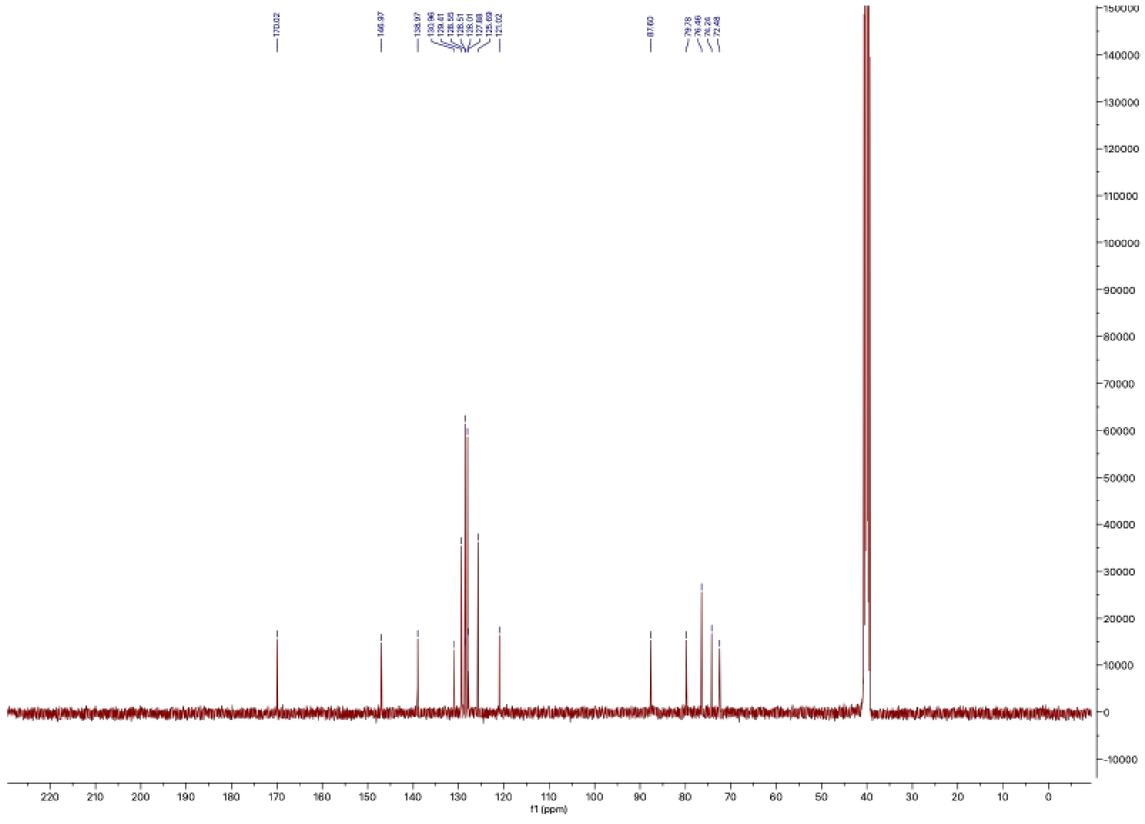

Figure S4: ^{13}C-NMR of compound **1a**

^{1}H and ^{13}C spectra of compound 1b

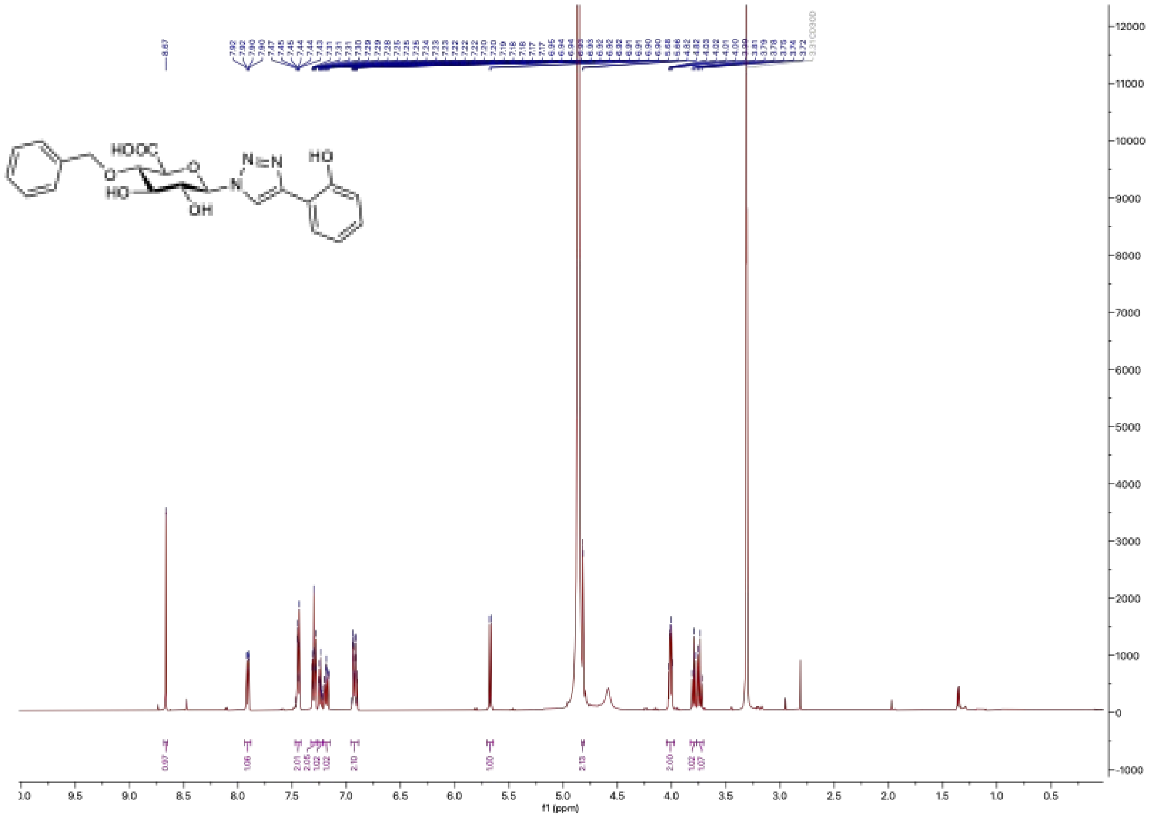

Figure S5: ^{1}H-NMR of compound **1b**

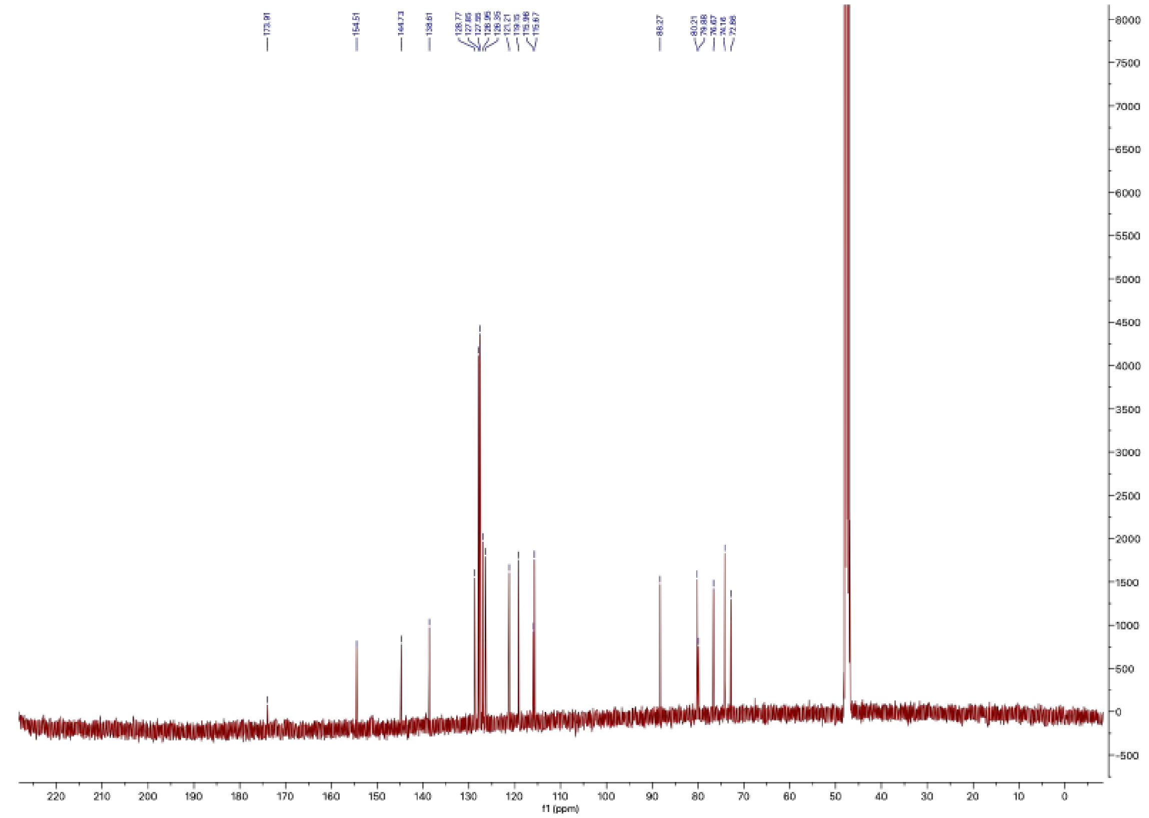

Figure S6: ^{13}C-NMR of compound **1b**

^{1}H and ^{13}C spectra of compound 1c

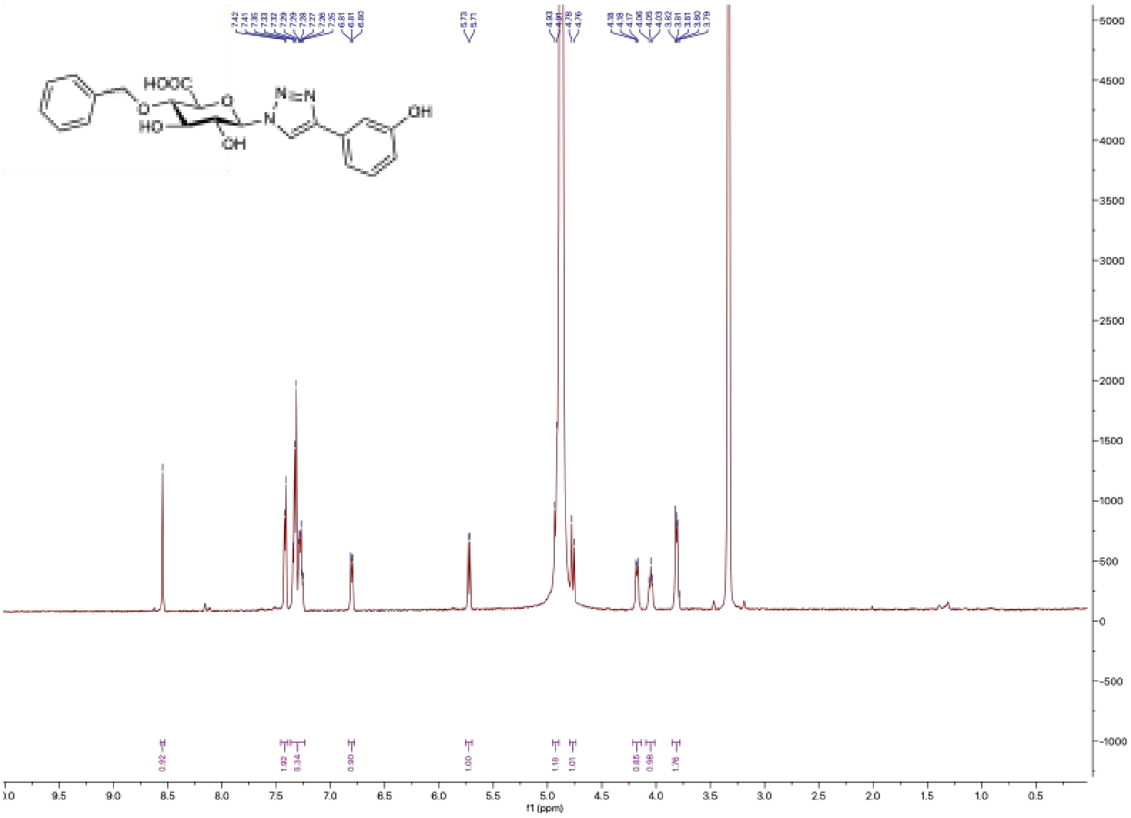

Figure S7: ^{1}H-NMR of compound **1c**

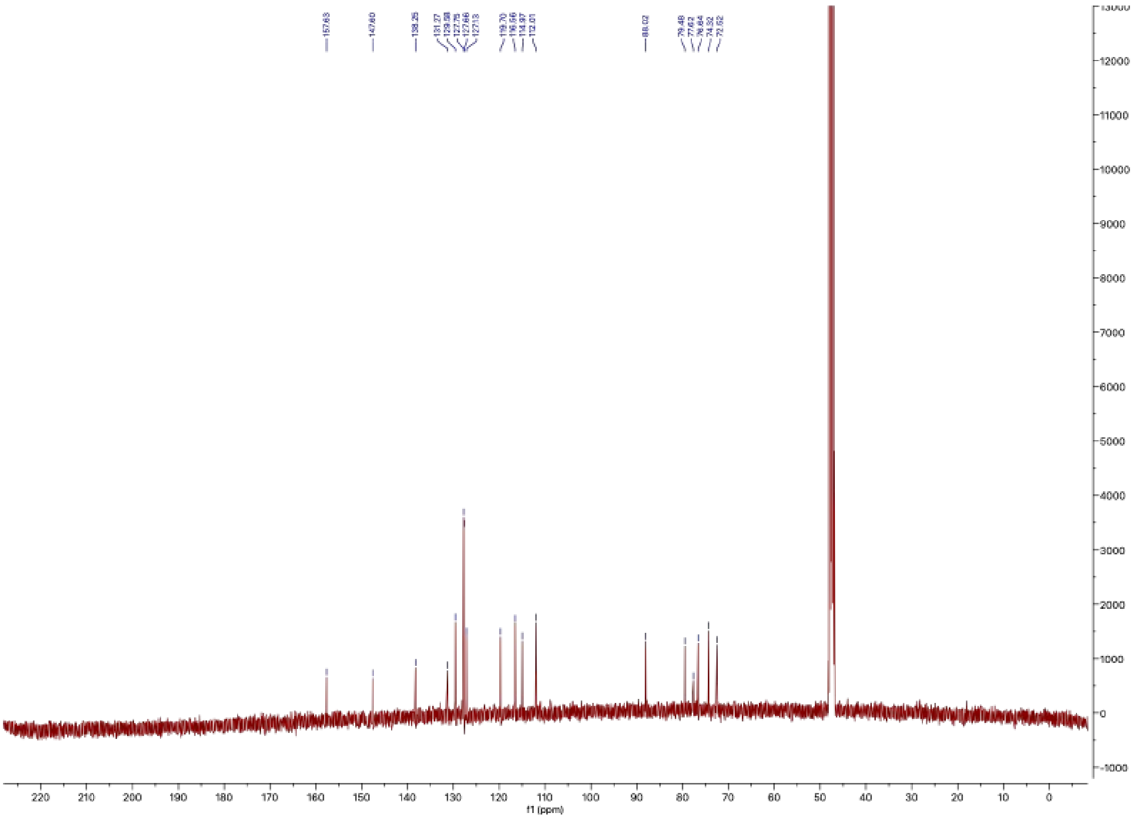

Figure S8: ^{13}C-NMR of compound **1c**

^{1}H and ^{13}C spectra of compound 1d

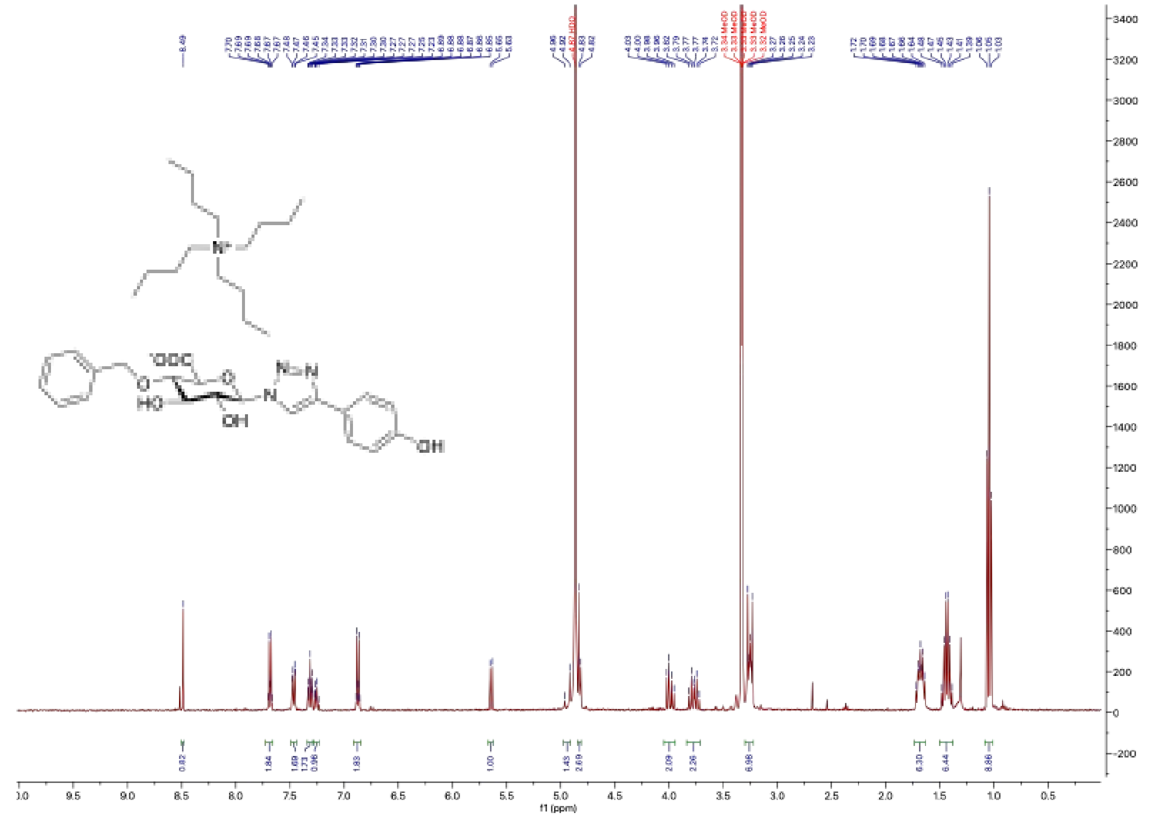

Figure S9: ^{1}H-NMR of compound **1d**

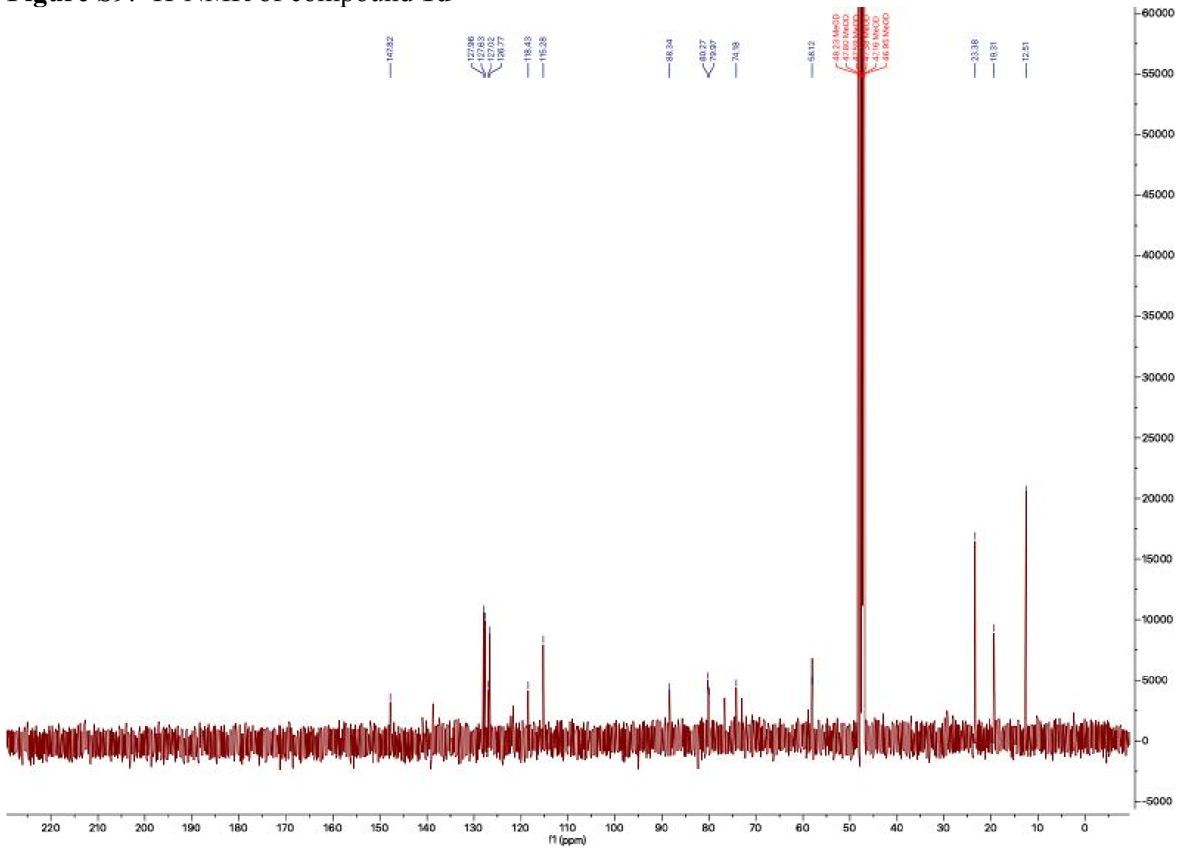

Figure S10: ^{13}C-NMR of compound **1d**

^{1}H and ^{13}C spectra of compound 1e

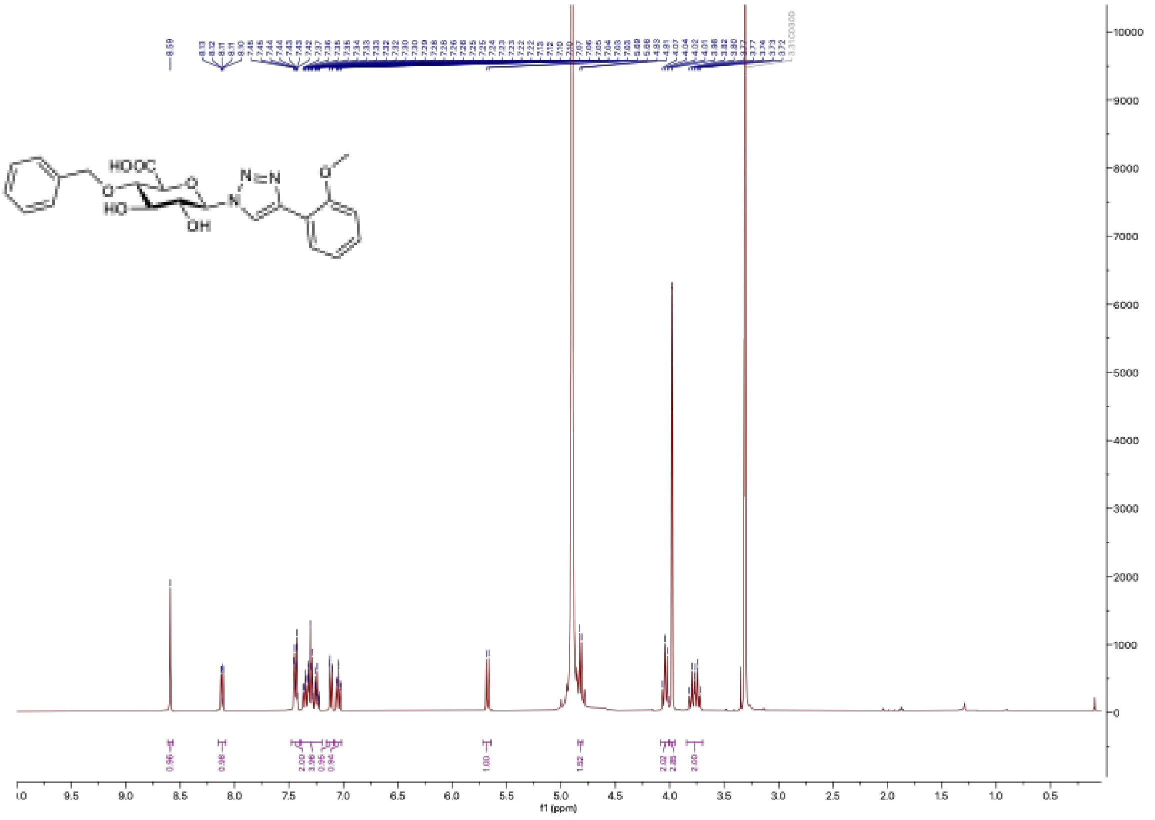

Figure S11: ^{1}H-NMR of compound **1e**

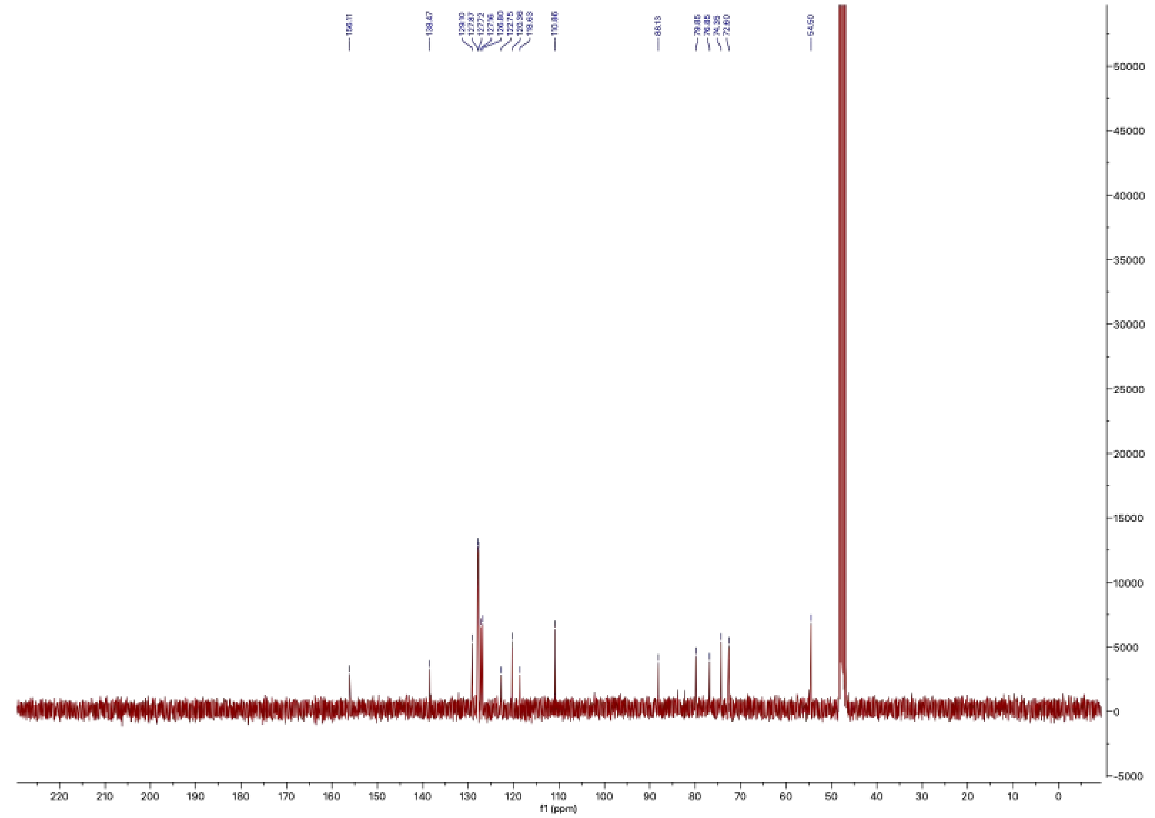

Figure S12: ^{13}C-NMR of compound **1e**

^{1}H and ^{13}C spectra of compound 1f

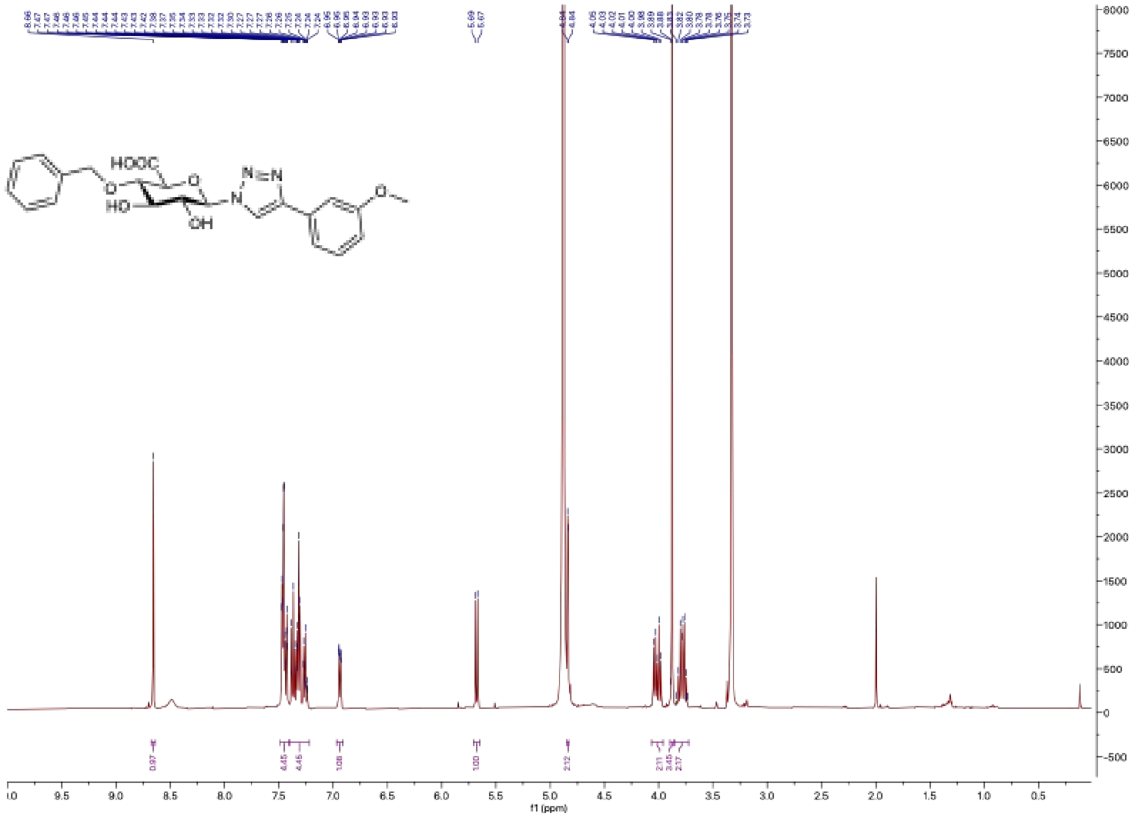

Figure S13: ^{1}H-NMR of compound **1f**

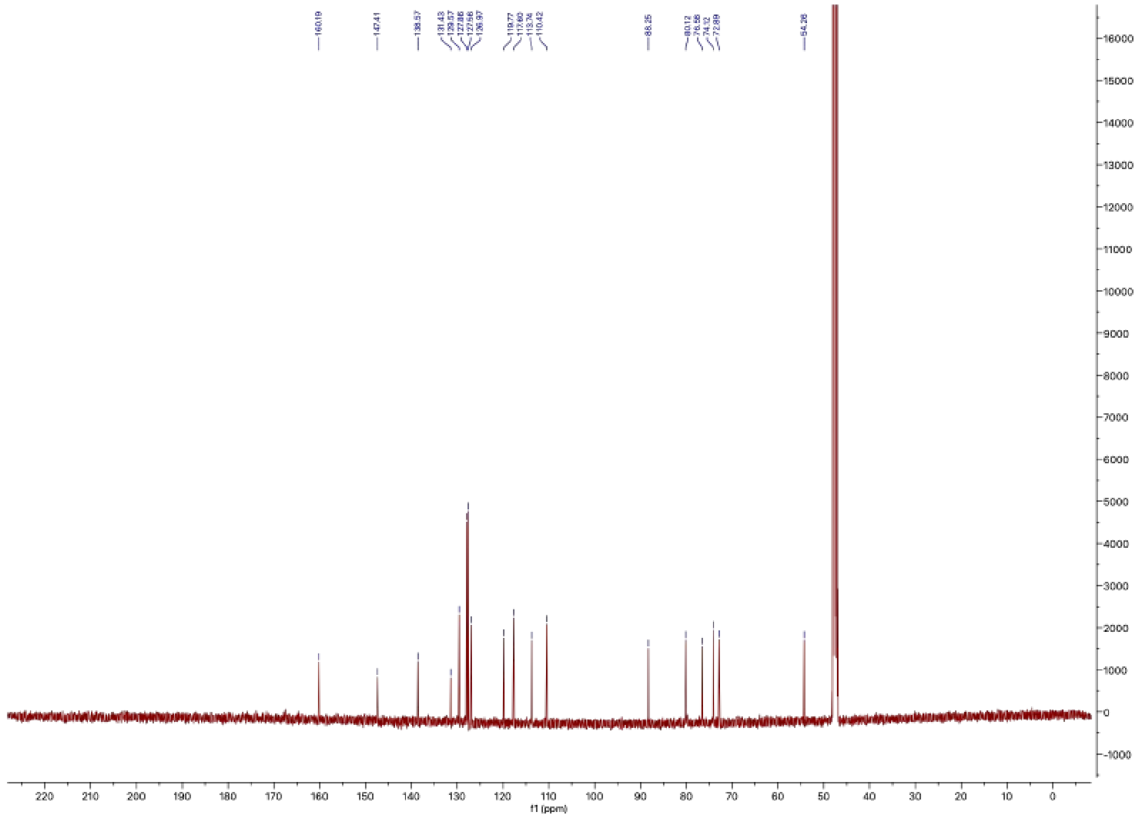

Figure S14: ^{13}C-NMR of compound **1f**

^{1}H and ^{13}C spectra of compound 1g

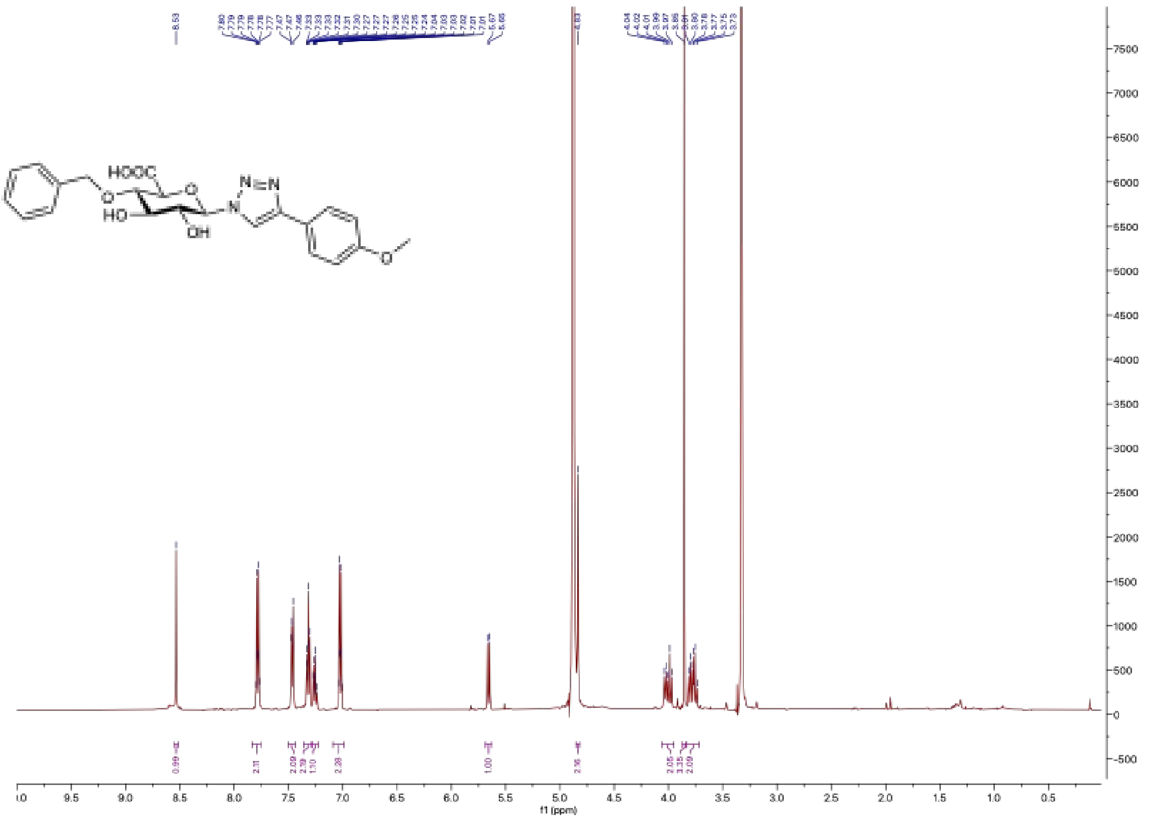

Figure S15: ^{1}H-NMR of compound **1g**

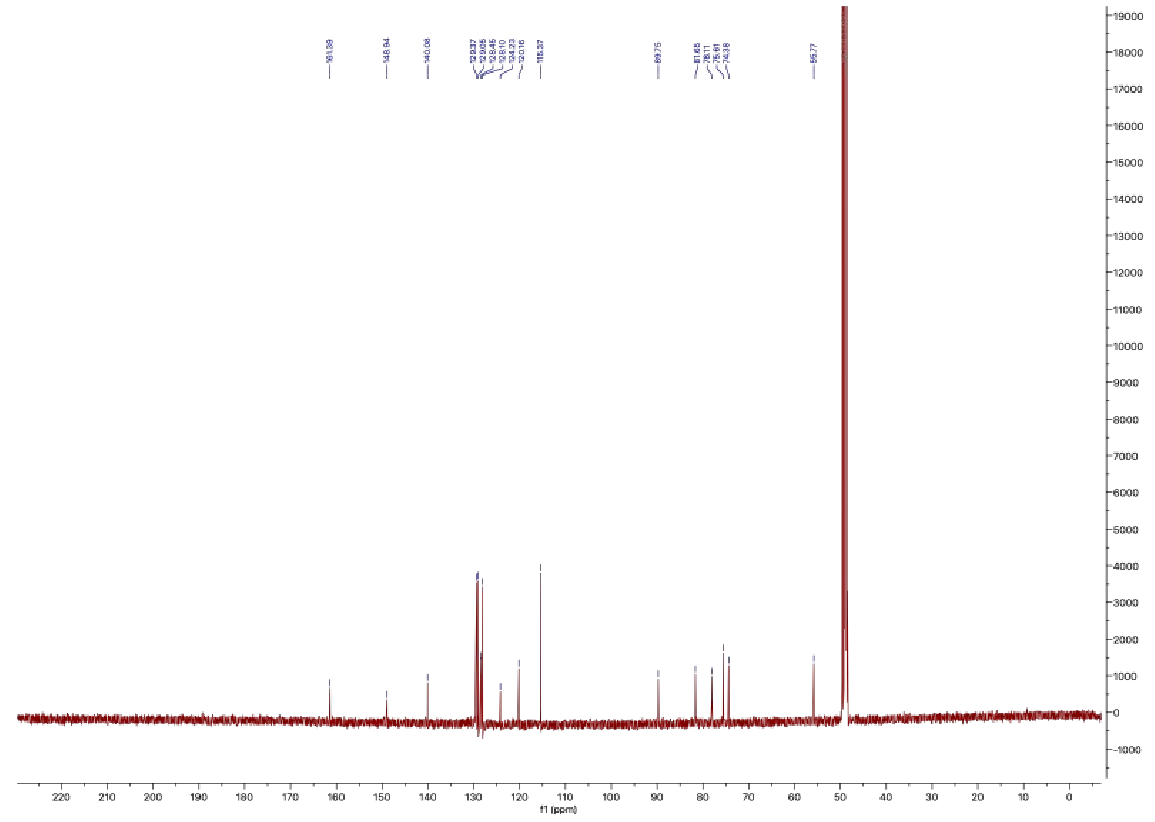

Figure S16: ^{13}C-NMR of compound **1g**

^{1}H and ^{13}C spectra of compound 1h

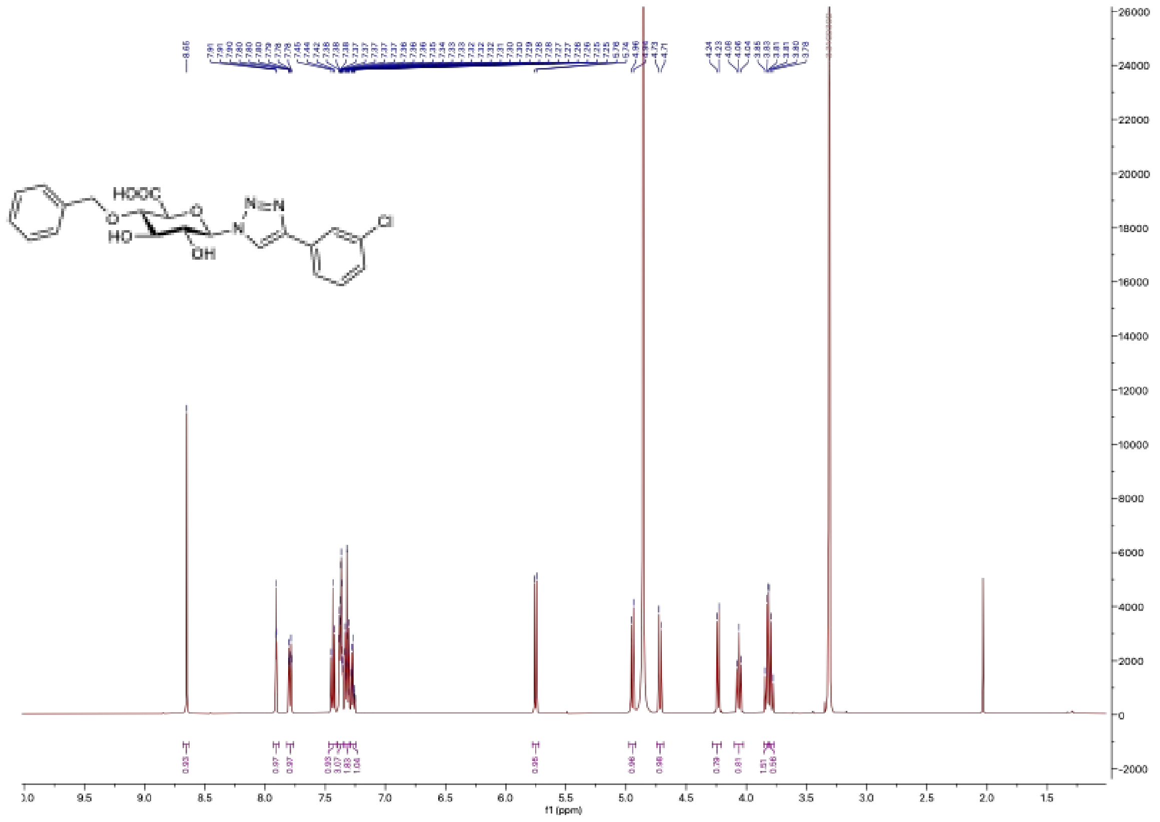

Figure S17: ^{1}H-NMR of compound **1h**

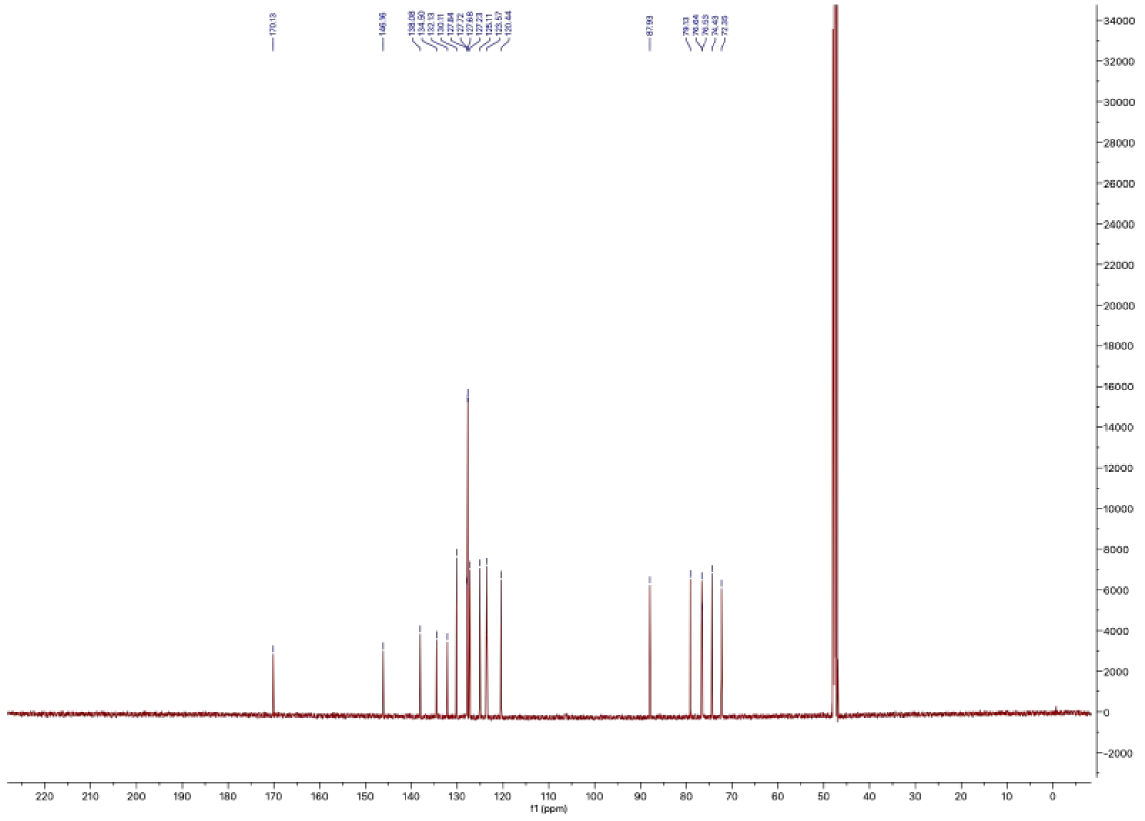

Figure S18: ^{13}C-NMR of compound **1h**

^{1}H and ^{13}C spectra of compound 1i

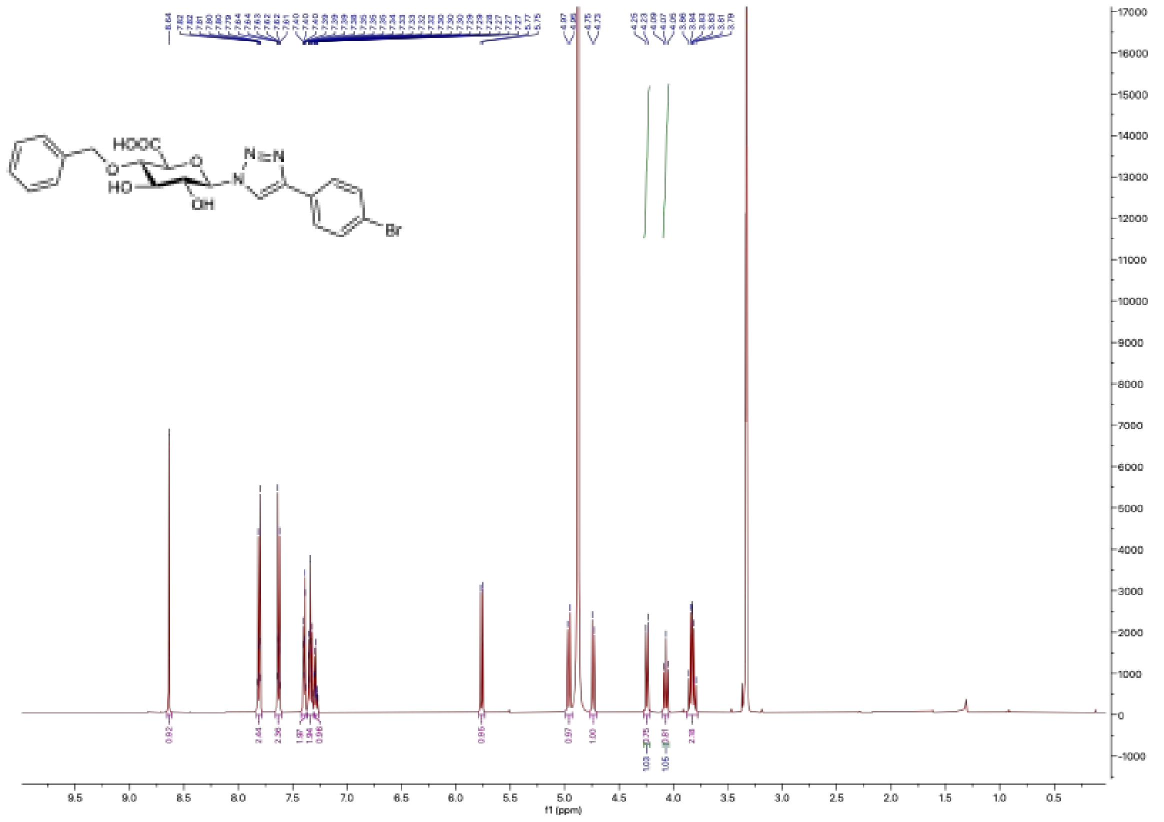

Figure S19: ^{1}H-NMR of compound **1i**

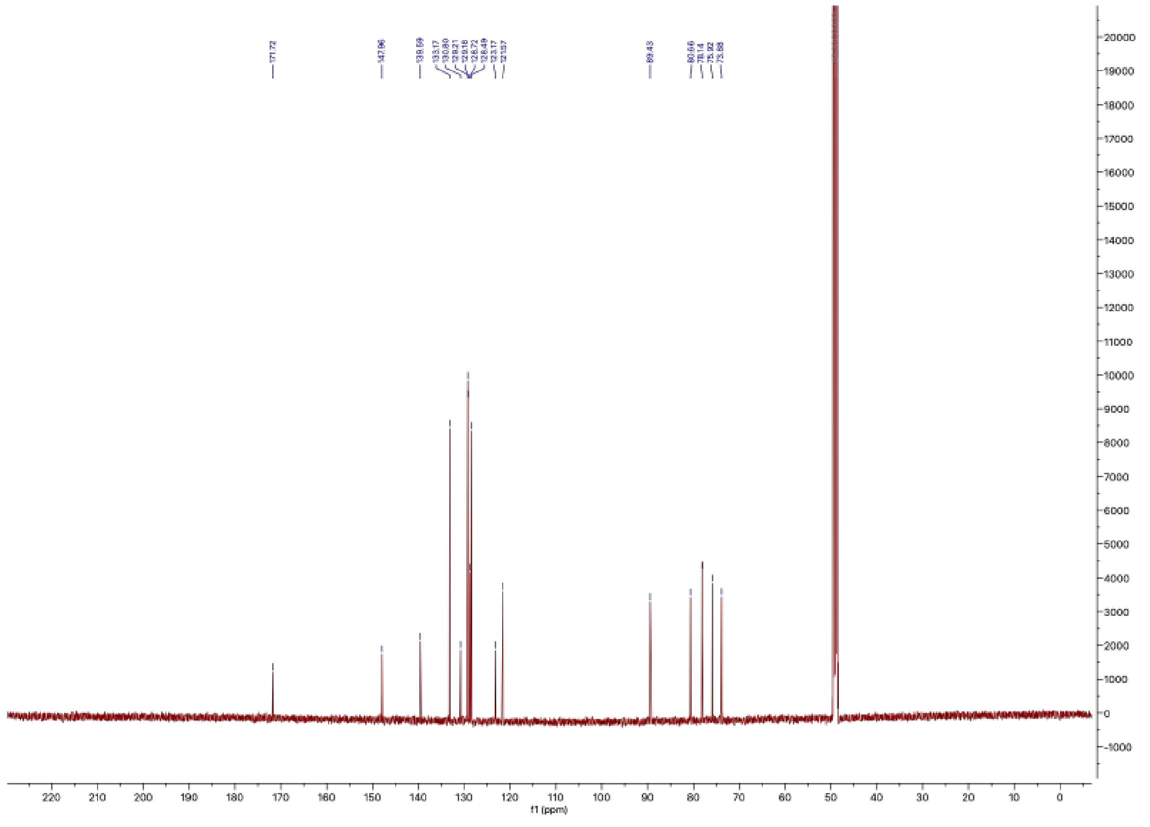

Figure S20: ^{13}C-NMR of compound **1i**

^{1}H and ^{13}C spectra of compound 1j

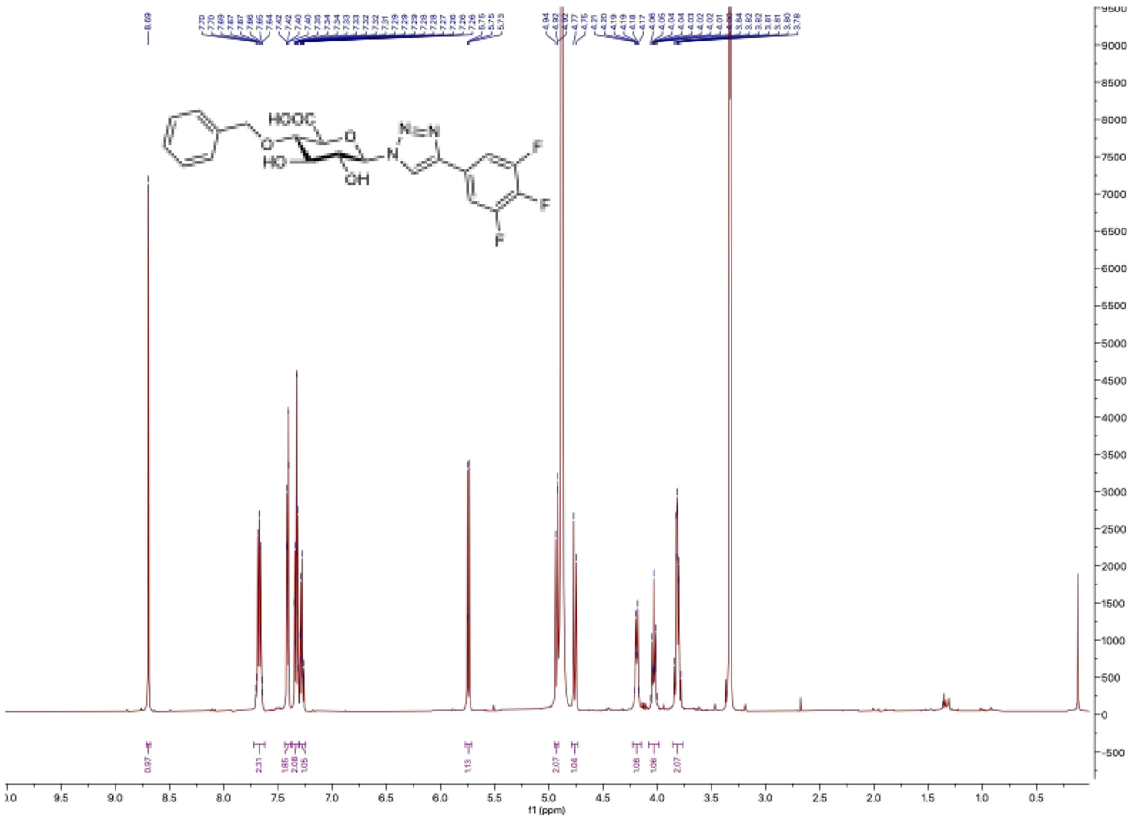

Figure S21: ^{1}H-NMR of compound **1j**

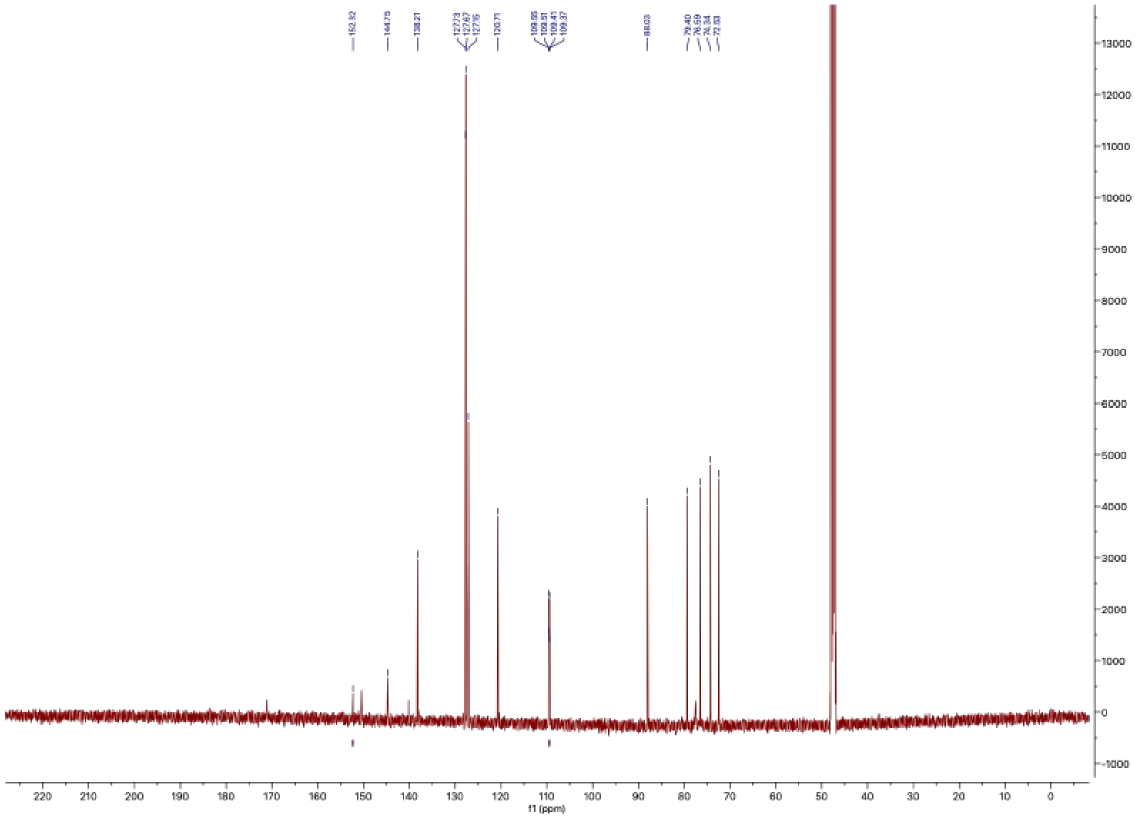

Figure S22: ^{13}C-NMR of compound **1j**

^{1}H and ^{13}C spectra of compound 1k

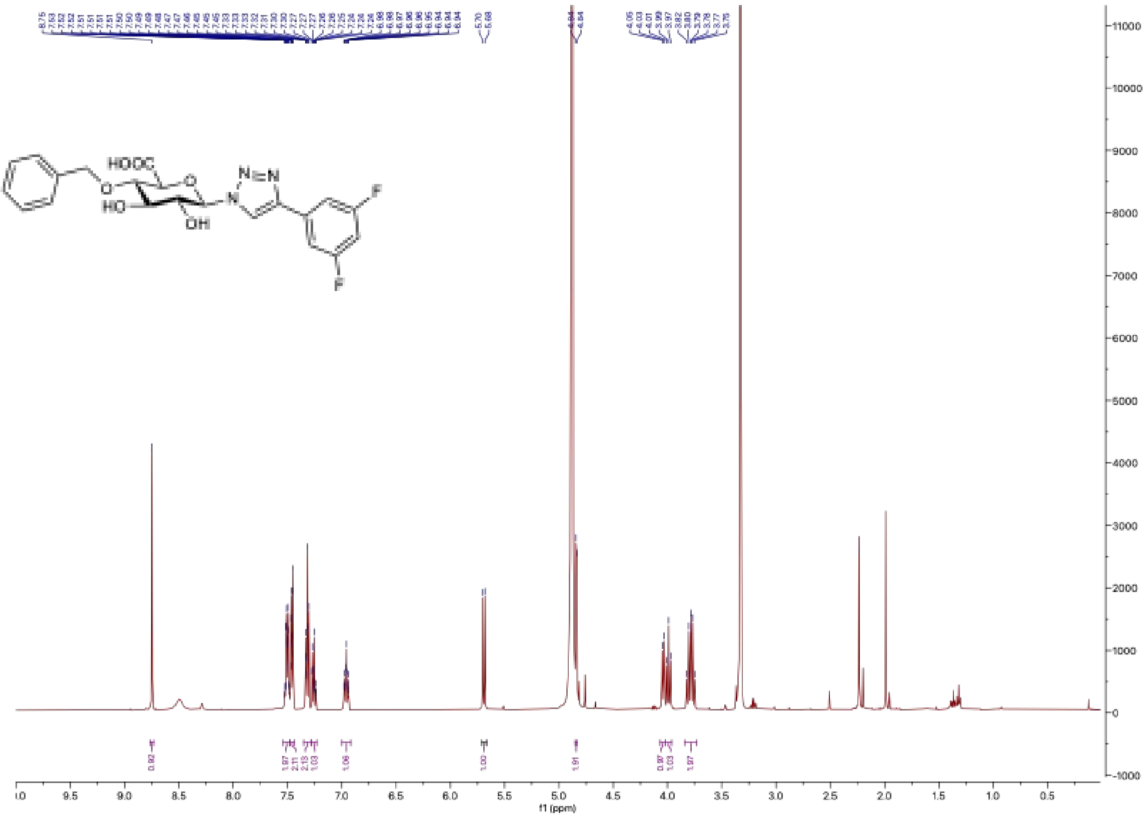

Figure S23: ^{1}H-NMR of compound **1k**

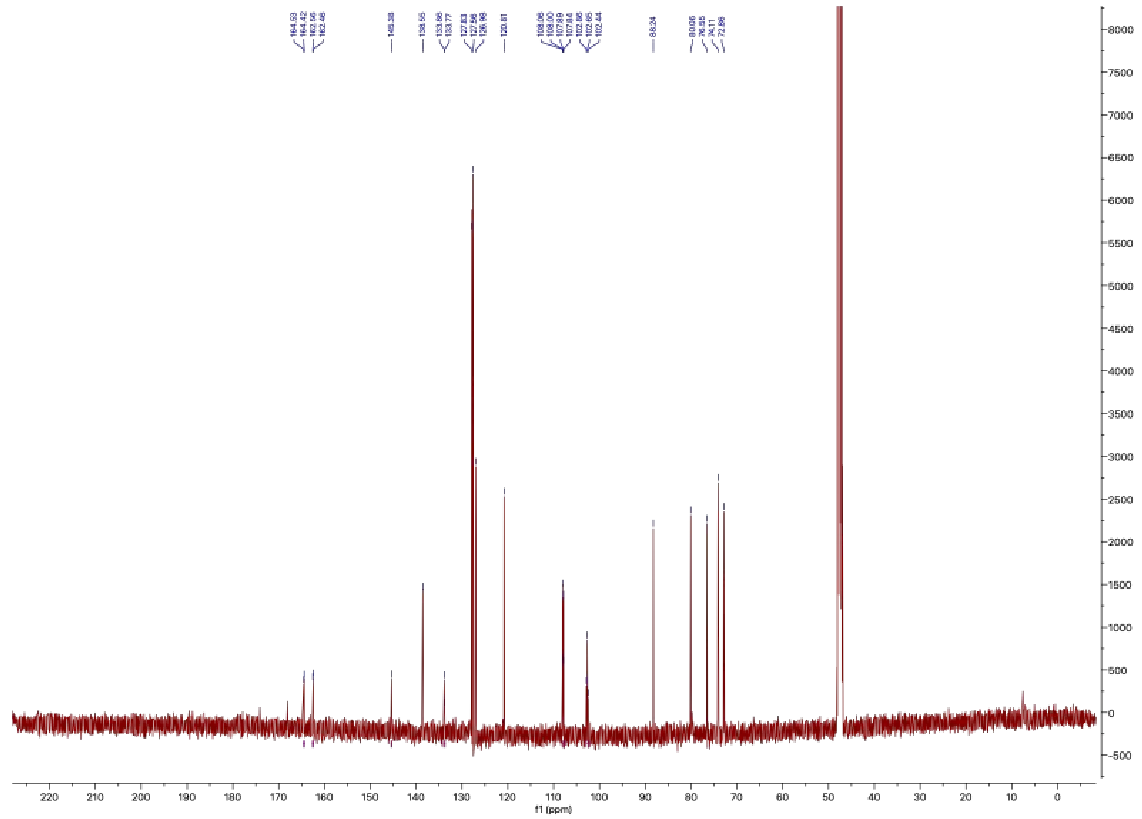

Figure S24: ^{13}C-NMR of compound **1k**

^{1}H and ^{13}C spectra of compound 1l

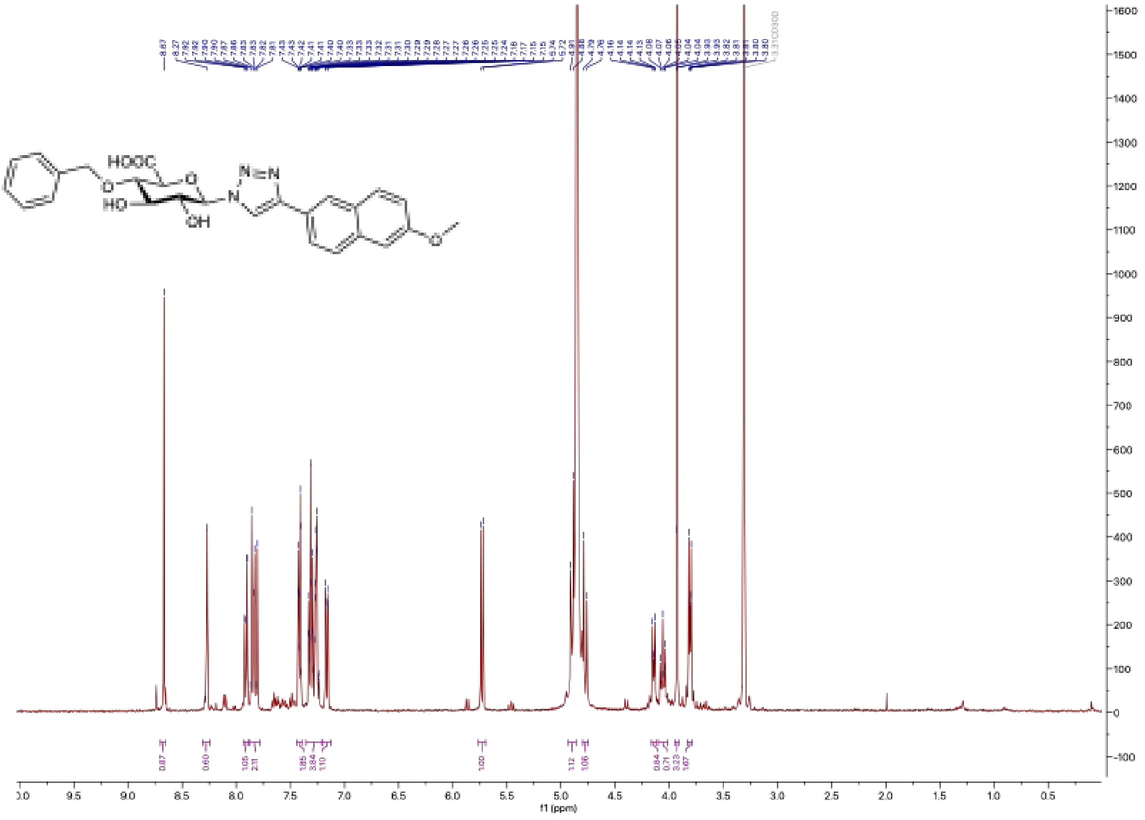

Figure S25: ^{1}H-NMR of compound **1l**

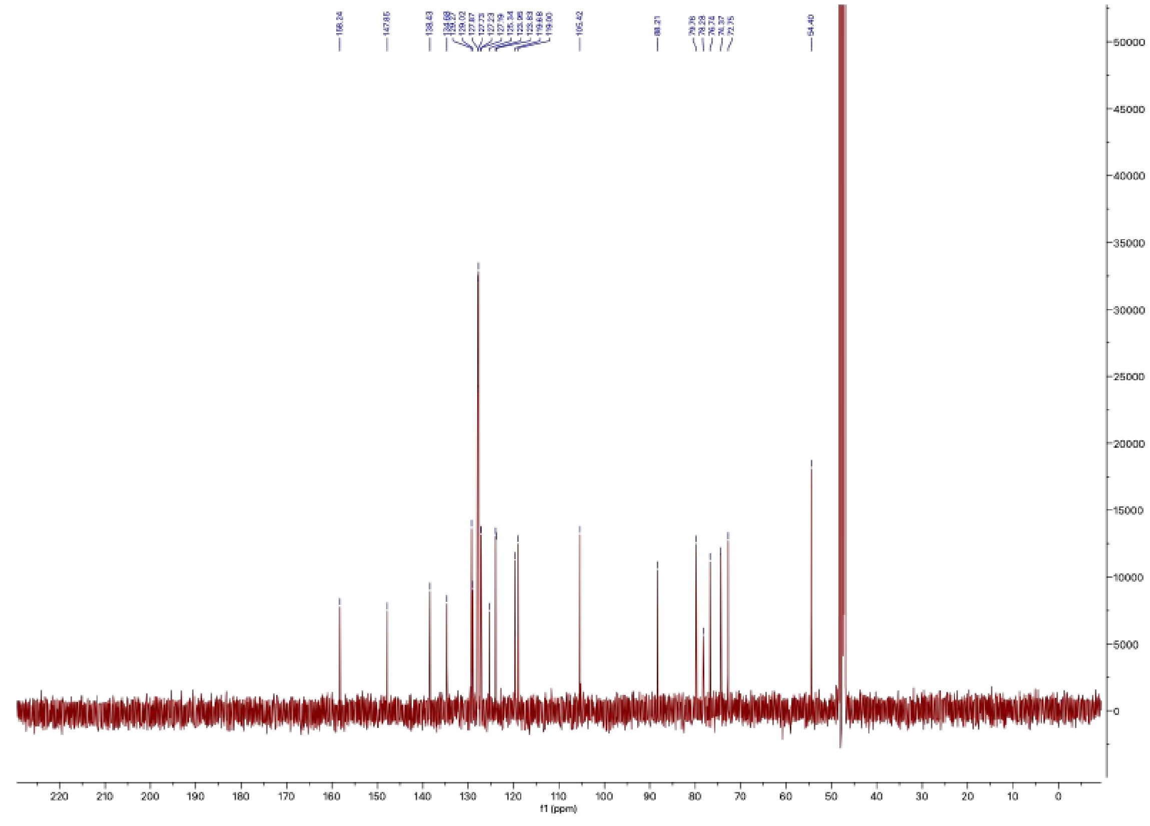

Figure S26: ^{13}C-NMR of compound **1l**

^{1}H and ^{13}C spectra of compound 1m

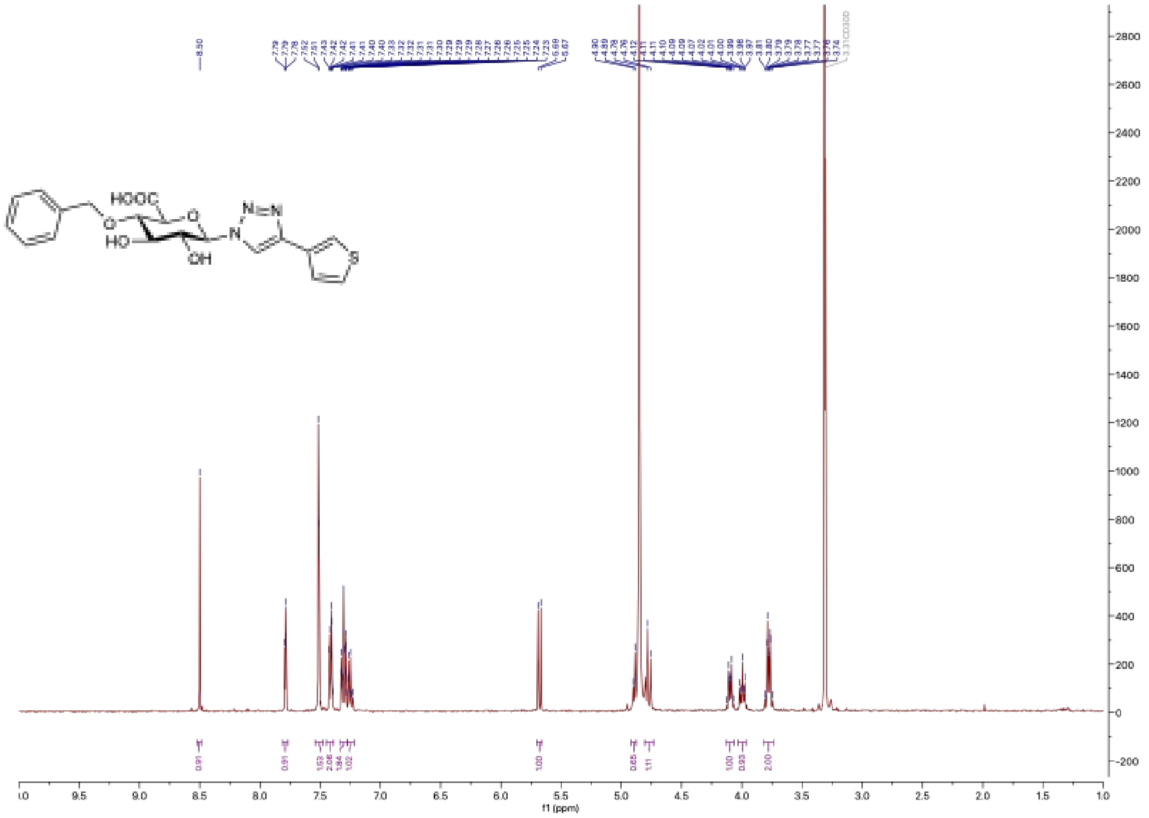

Figure S27: ^{1}H-NMR of compound **1m**

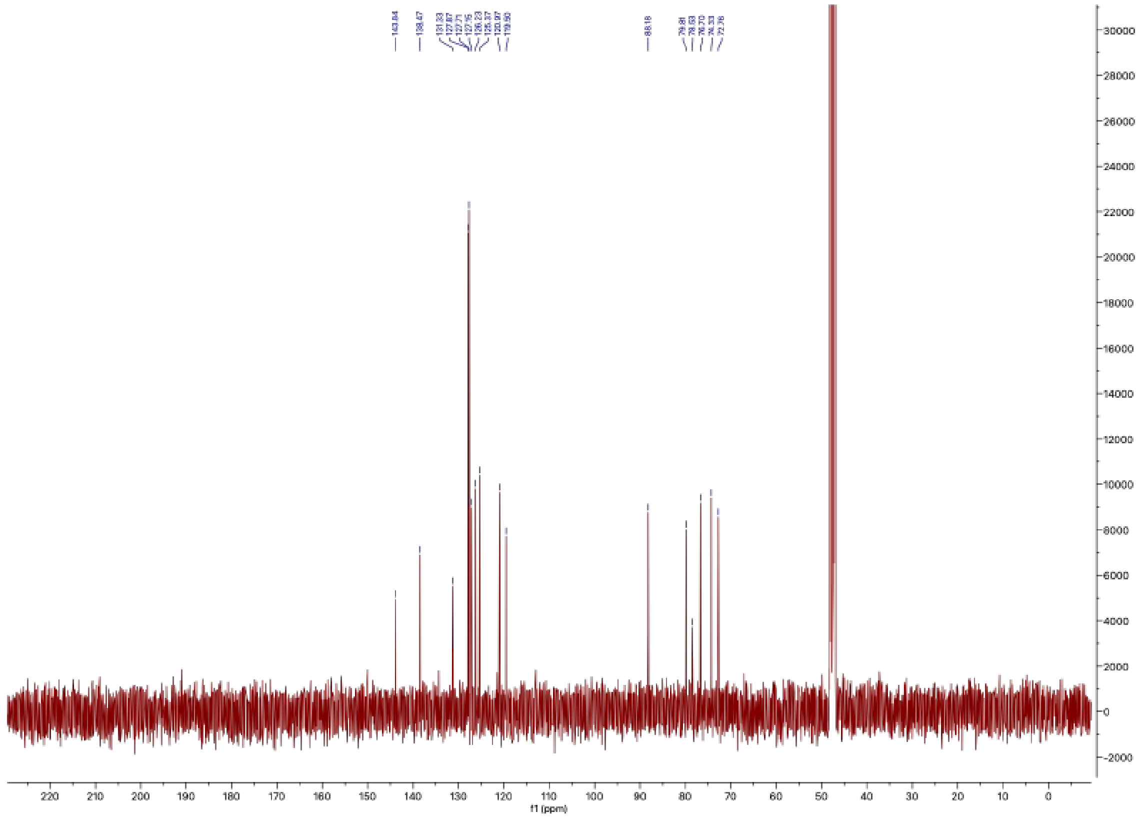

Figure S28: ^{13}C-NMR of compound **1m**

^{1}H and ^{13}C spectra of compound 1n

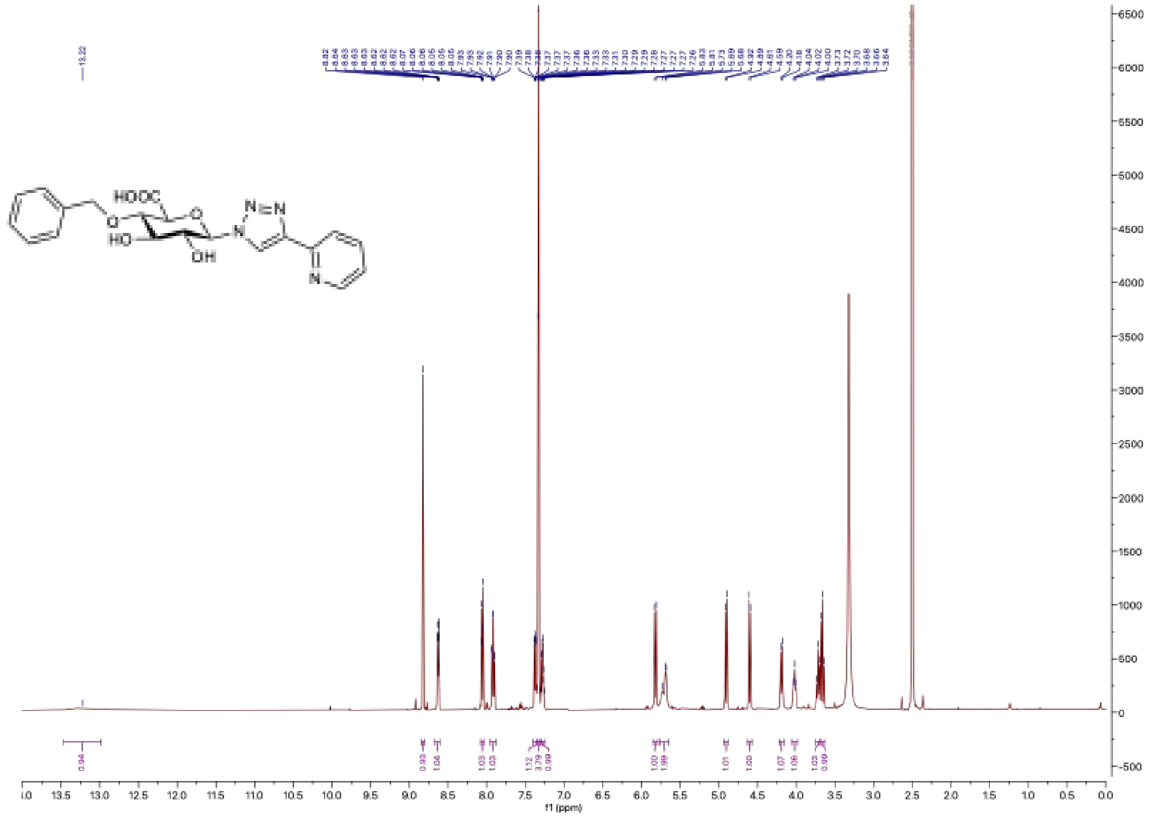

Figure S29: ^{1}H-NMR of compound **1n**

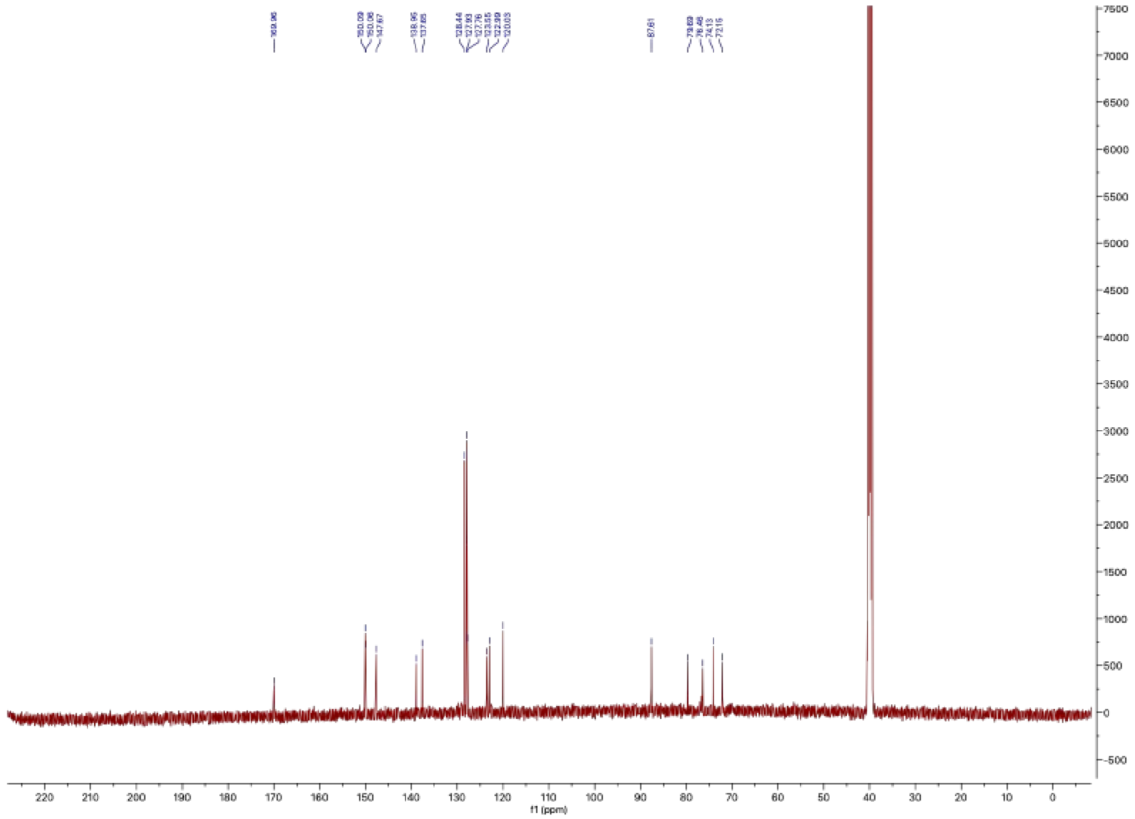

Figure S30: ^{13}C-NMR of compound **1n**

^{1}H and ^{13}C spectra of compound 1o

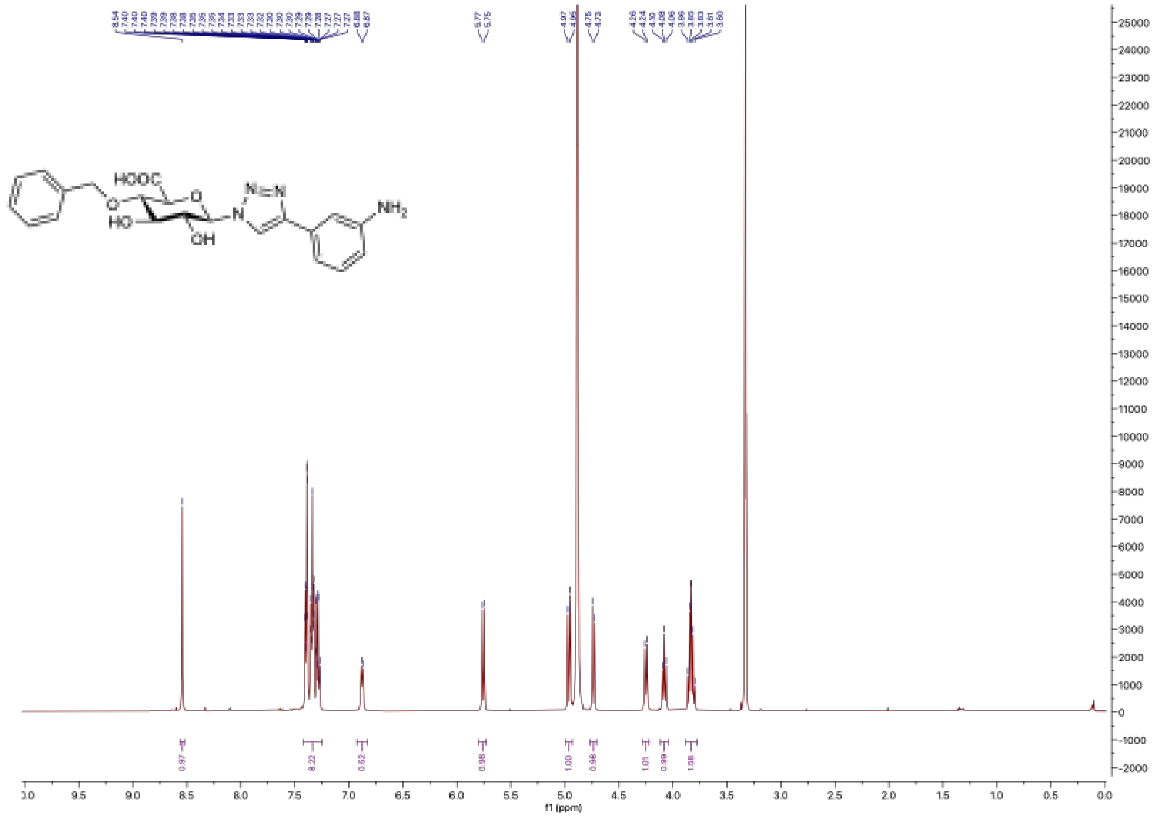

Figure S31: ^{1}H-NMR of compound **1o**

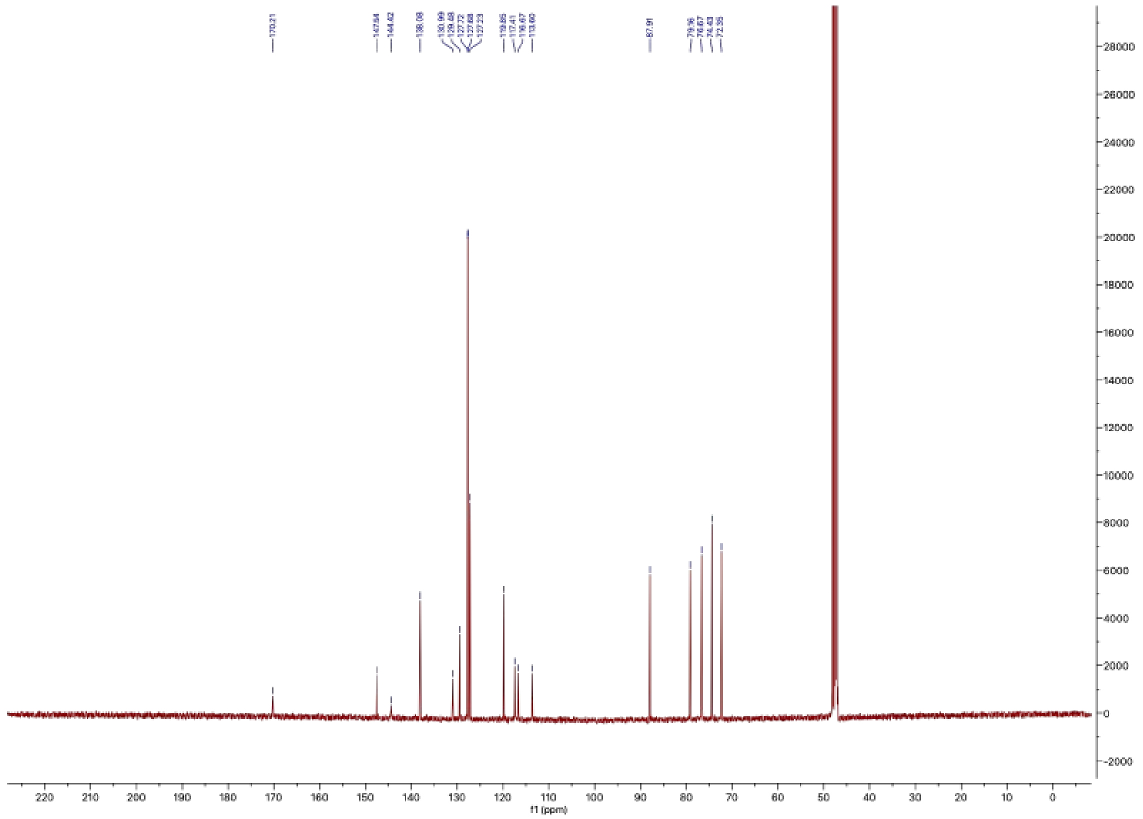

Figure S32: ^{13}C-NMR of compound **1o**

^{1}H and ^{13}C spectra of compound 1p

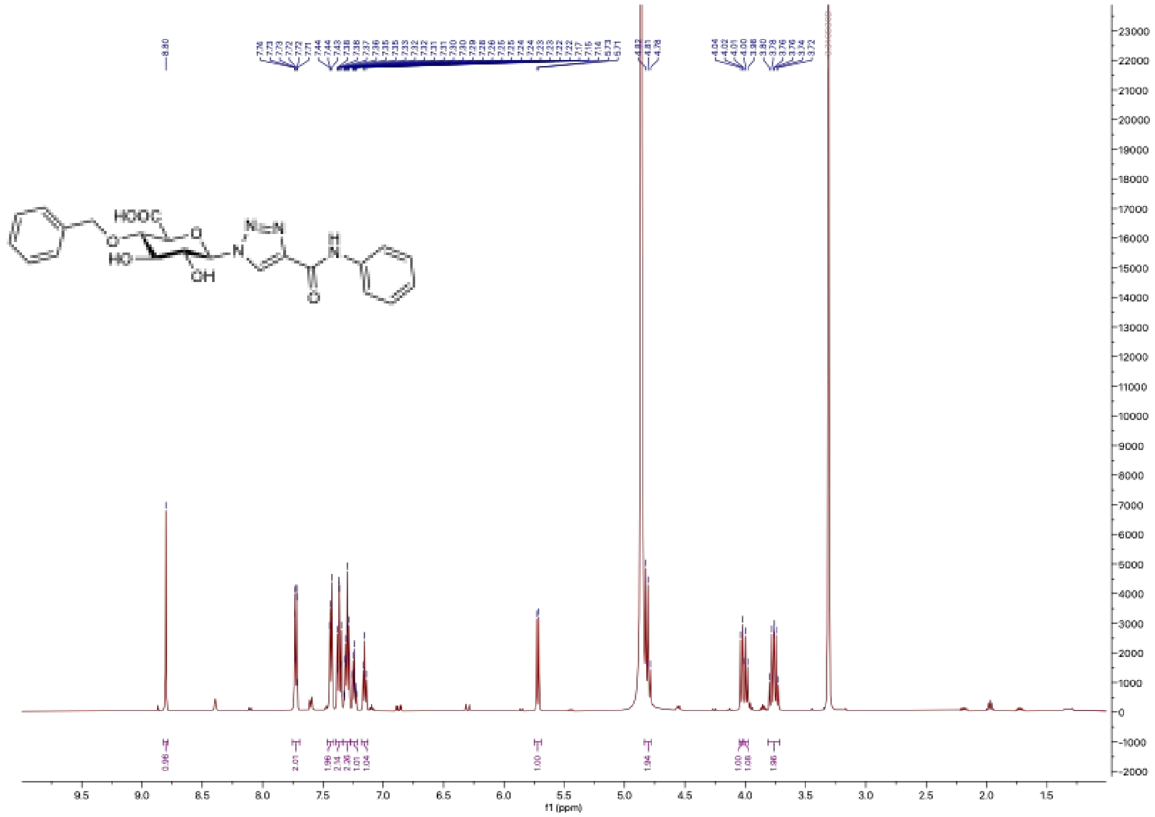

Figure S33: ^{1}H-NMR of compound **1p**

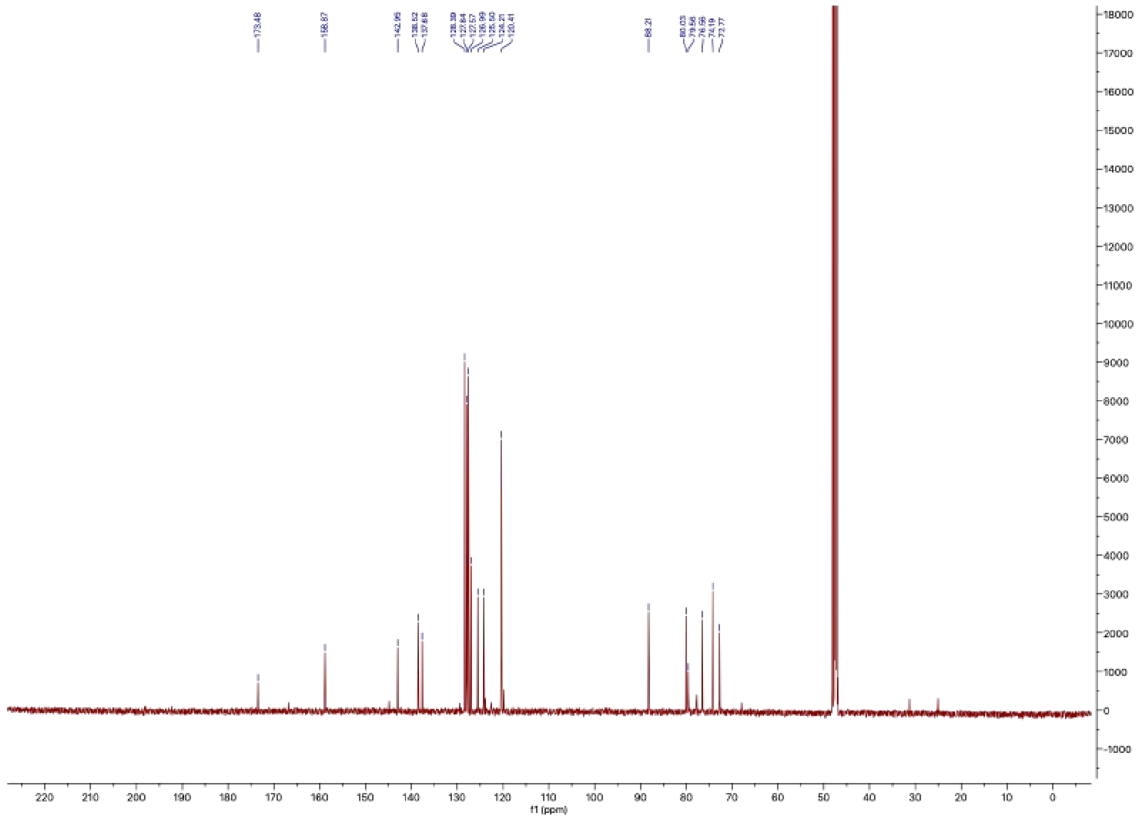

Figure S34: ^{13}C-NMR of compound **1p**

^{1}H and ^{13}C spectra of compound 1q

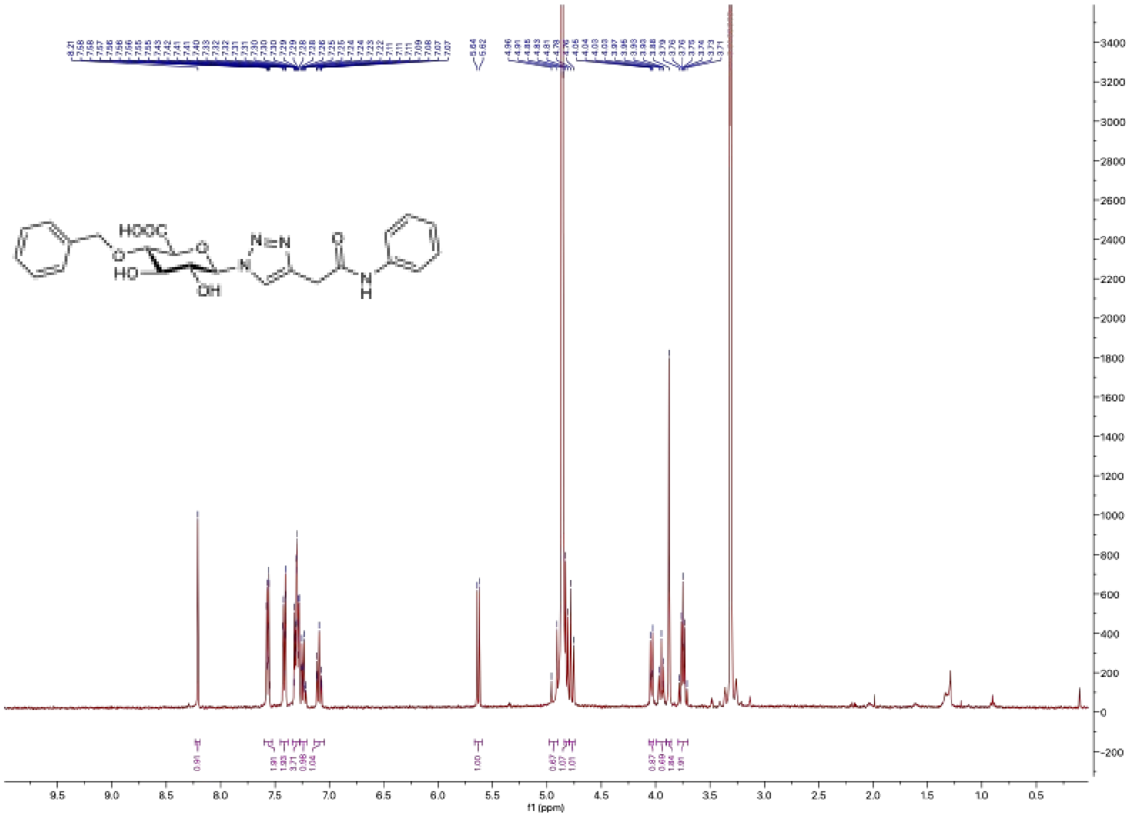

Figure S35: ^{1}H-NMR of compound **1q**

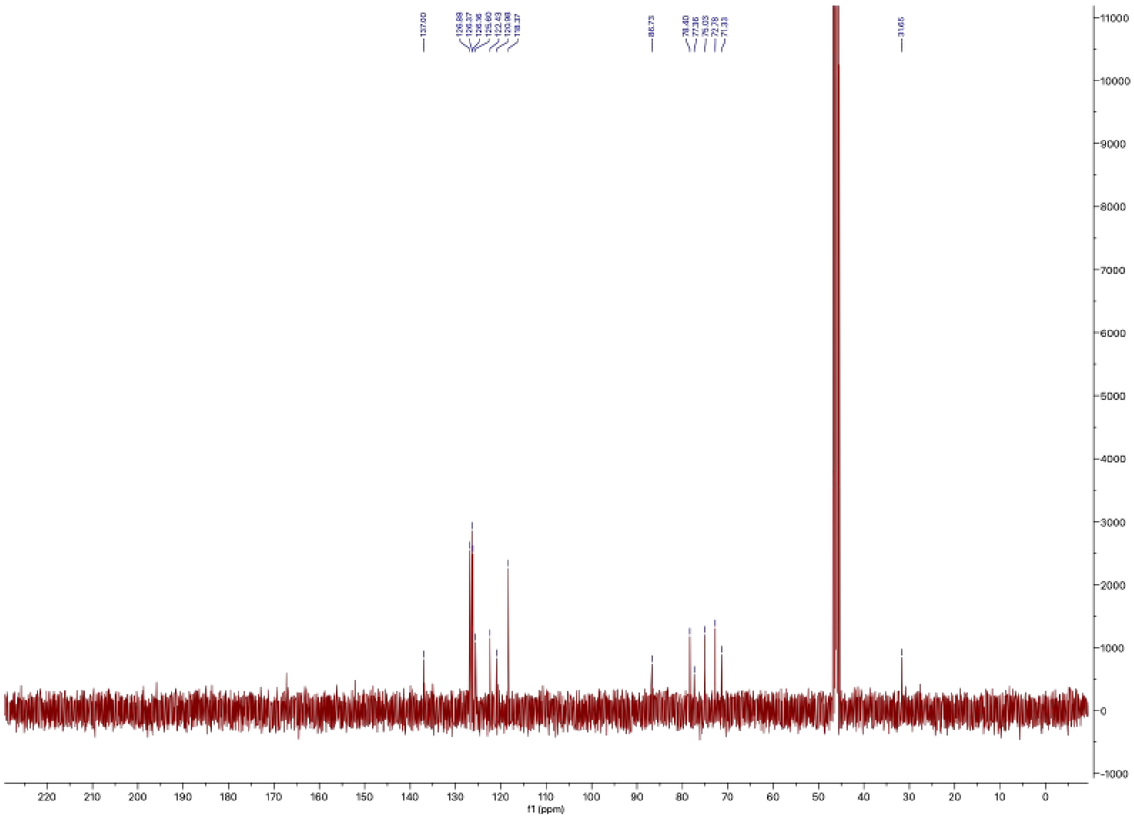

Figure S36: ^{13}C-NMR of compound **1q**

^{1}H and ^{13}C spectra of compound 1r

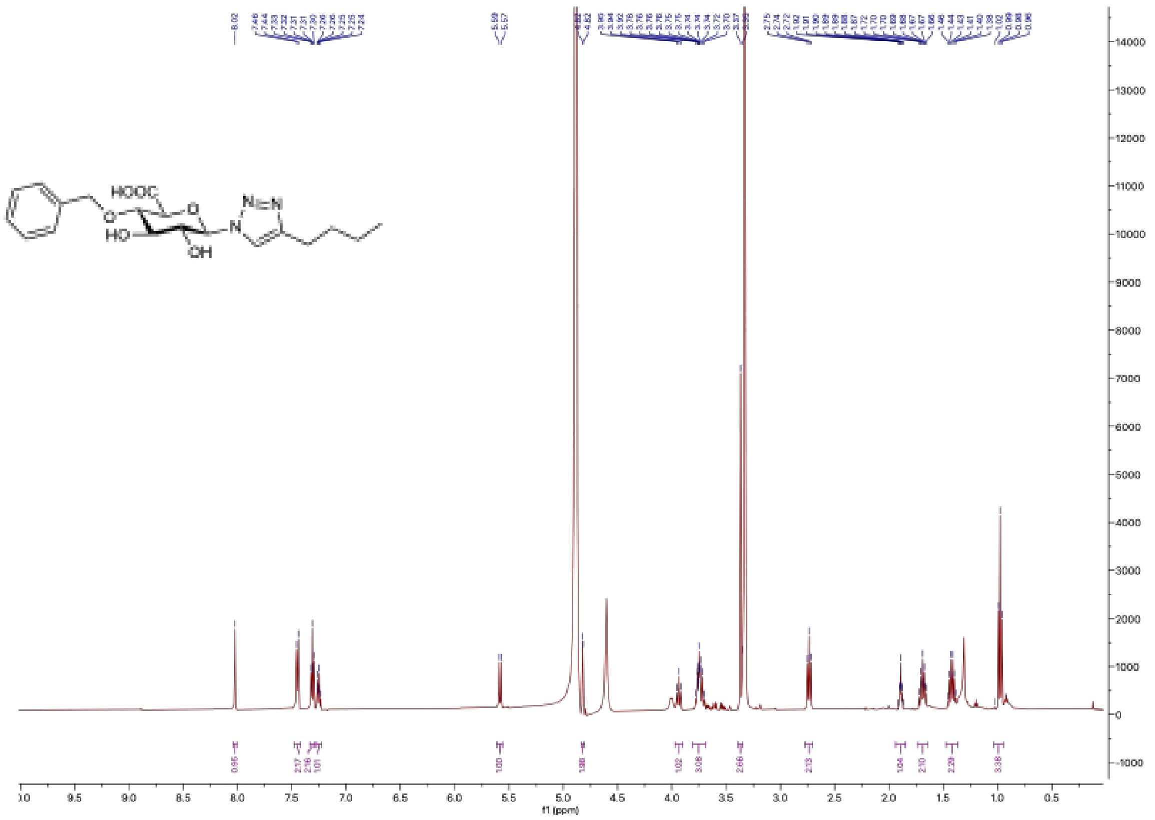

Figure S37: ^{1}H-NMR of compound **1r**

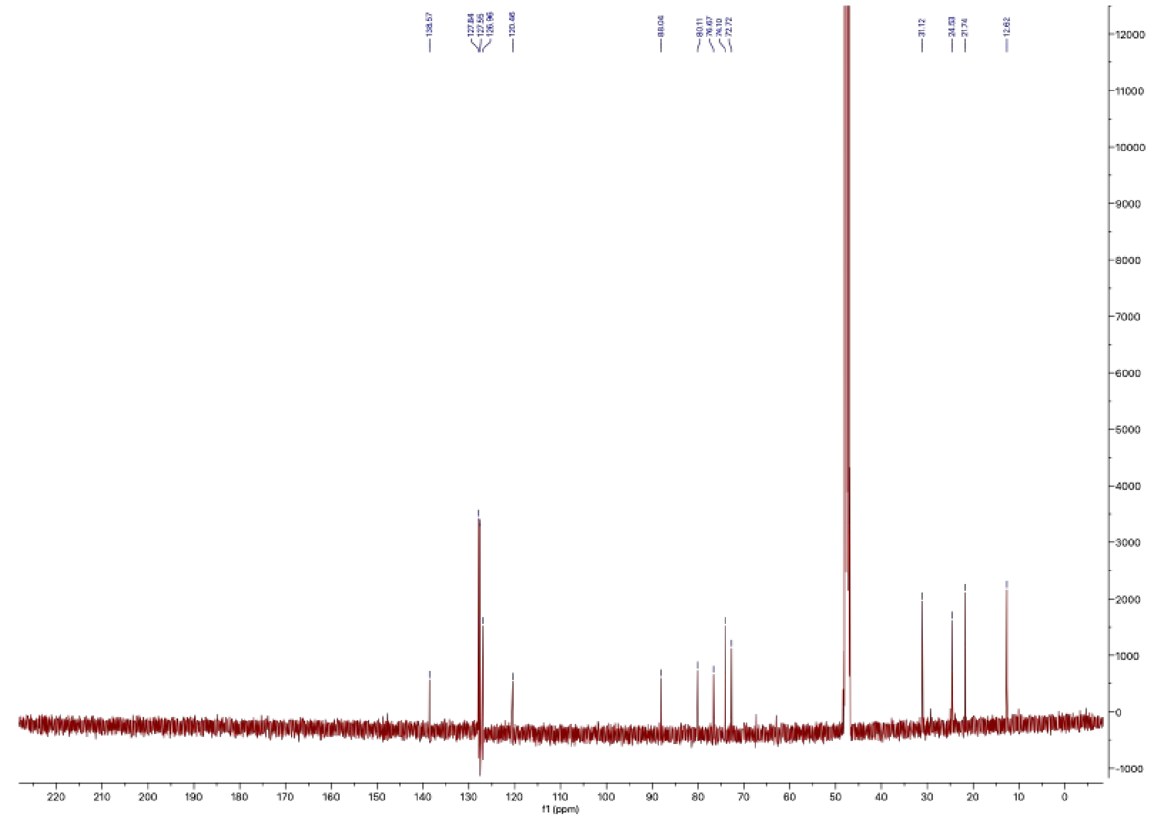

Figure S38: ^{13}C-NMR of compound **1r**

^{1}H and ^{13}C spectra of compound 1s

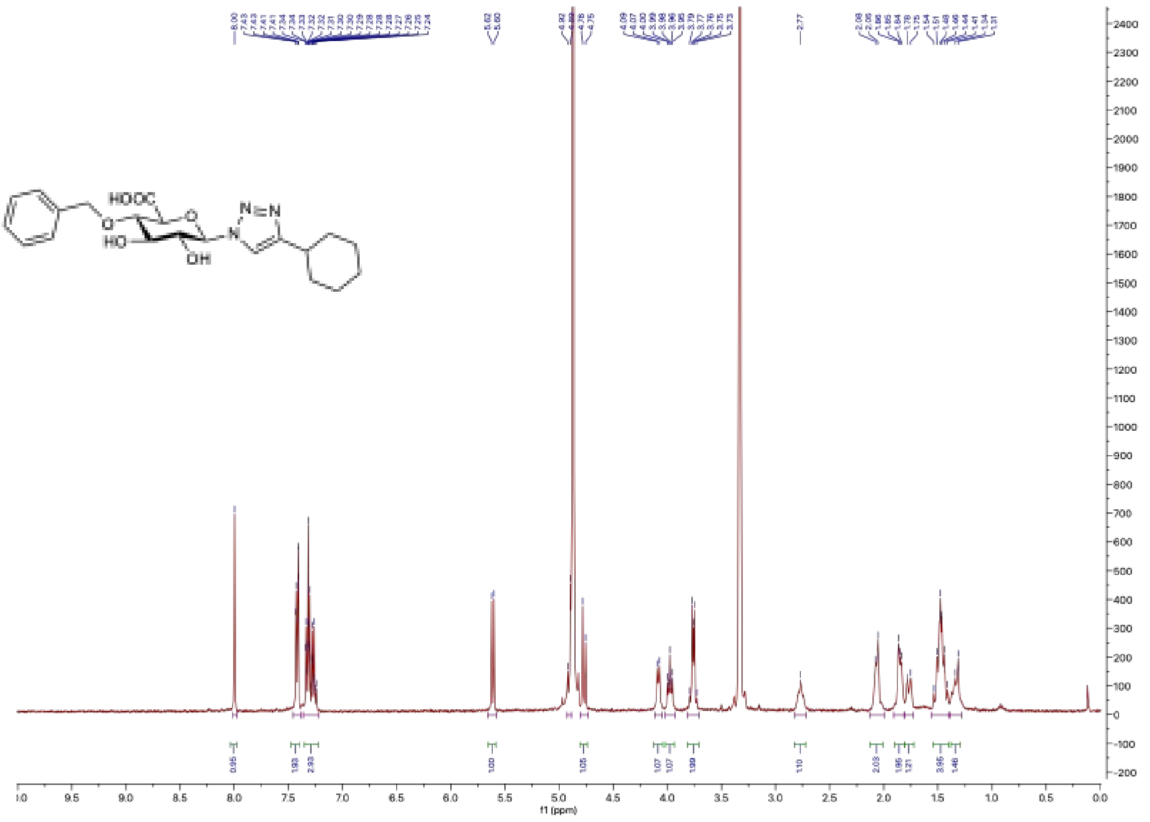

Figure S39: ^{1}H-NMR of compound **1s**

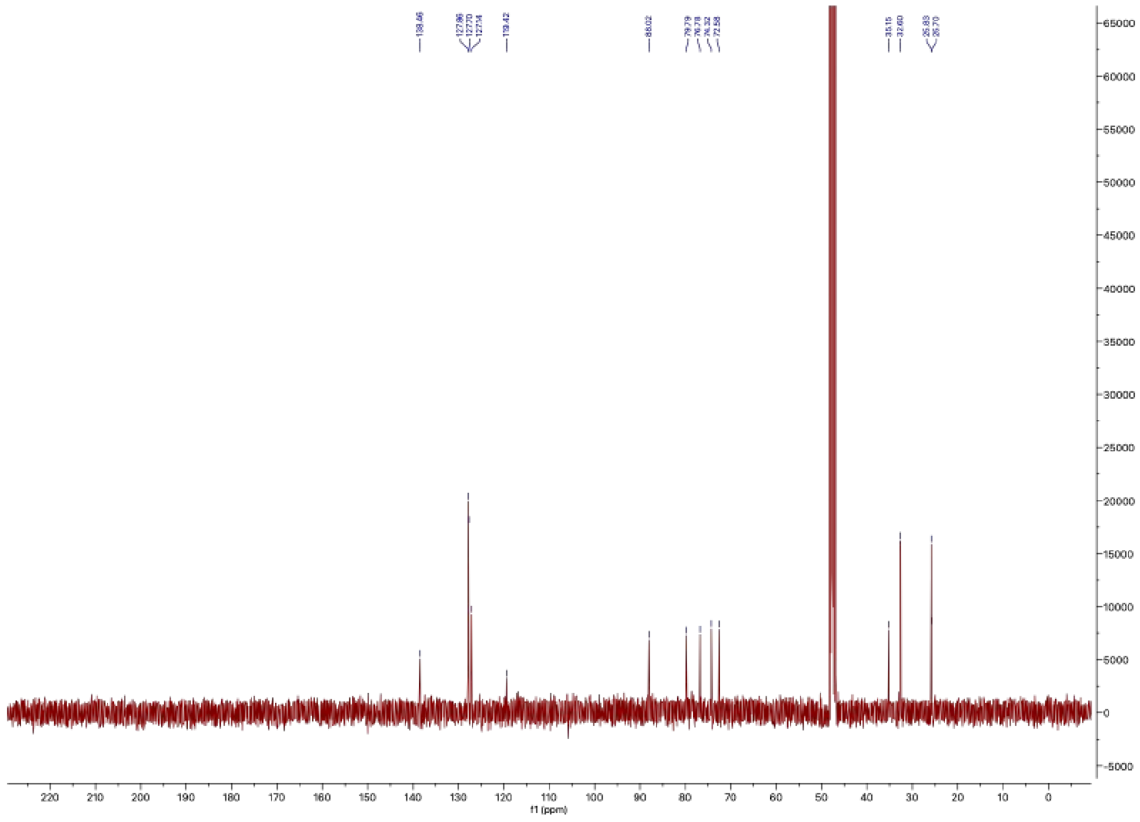

Figure S40: ^{13}C-NMR of compound **1s**

^{1}H and ^{13}C spectra of compound 2

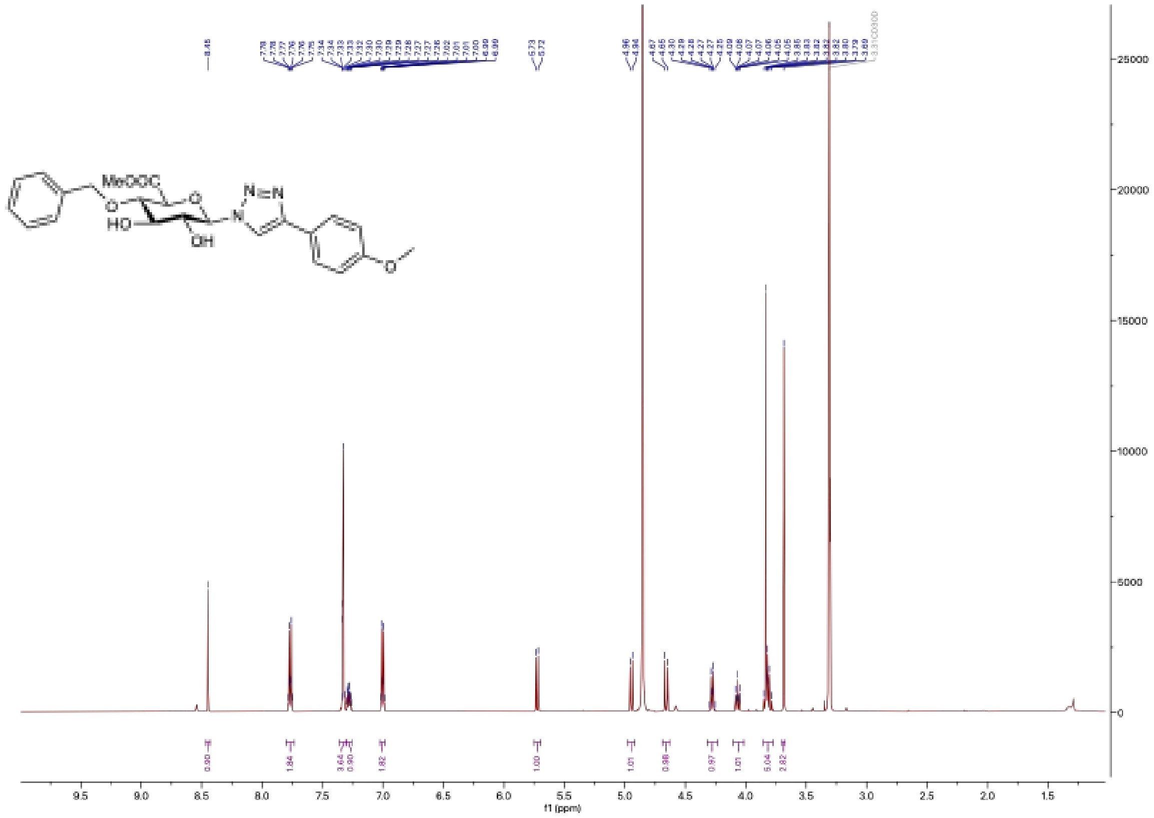

Figure S41: ^{1}H-NMR of compound **2**

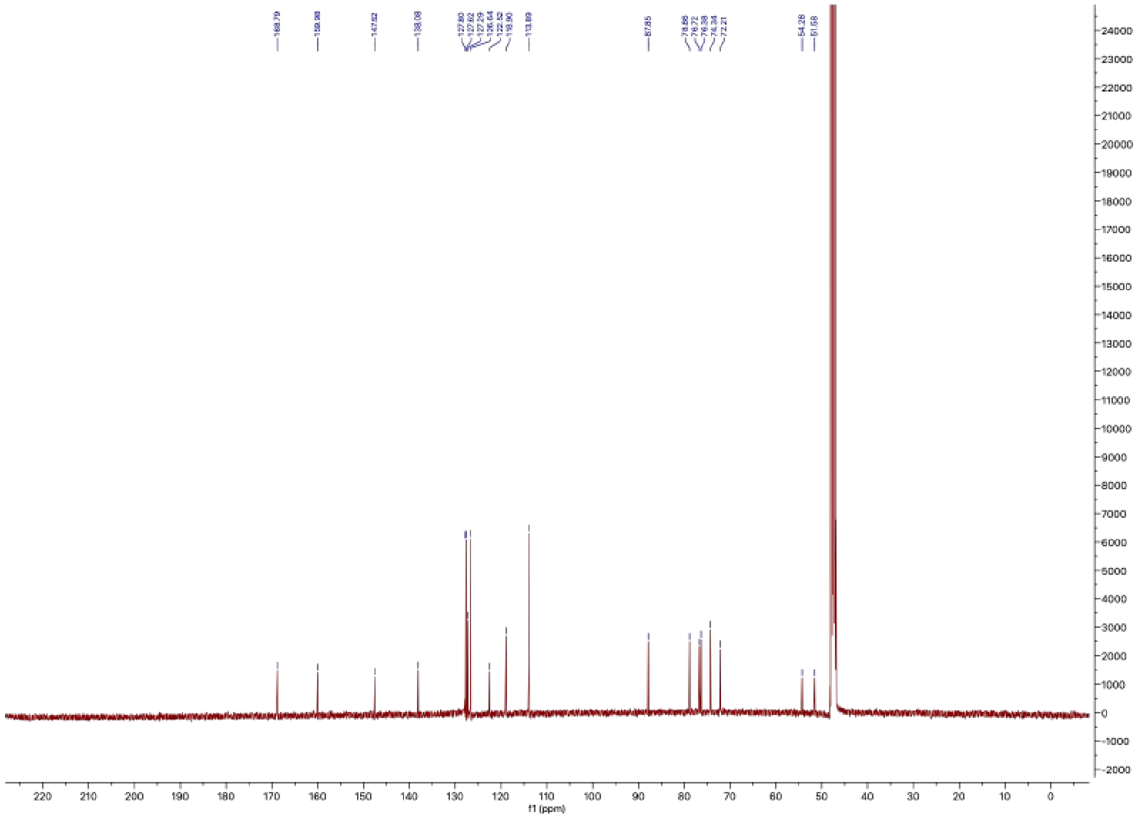

Figure S42: ^{13}C-NMR of compound **2**

^{1}H and ^{13}C spectra of compound 3

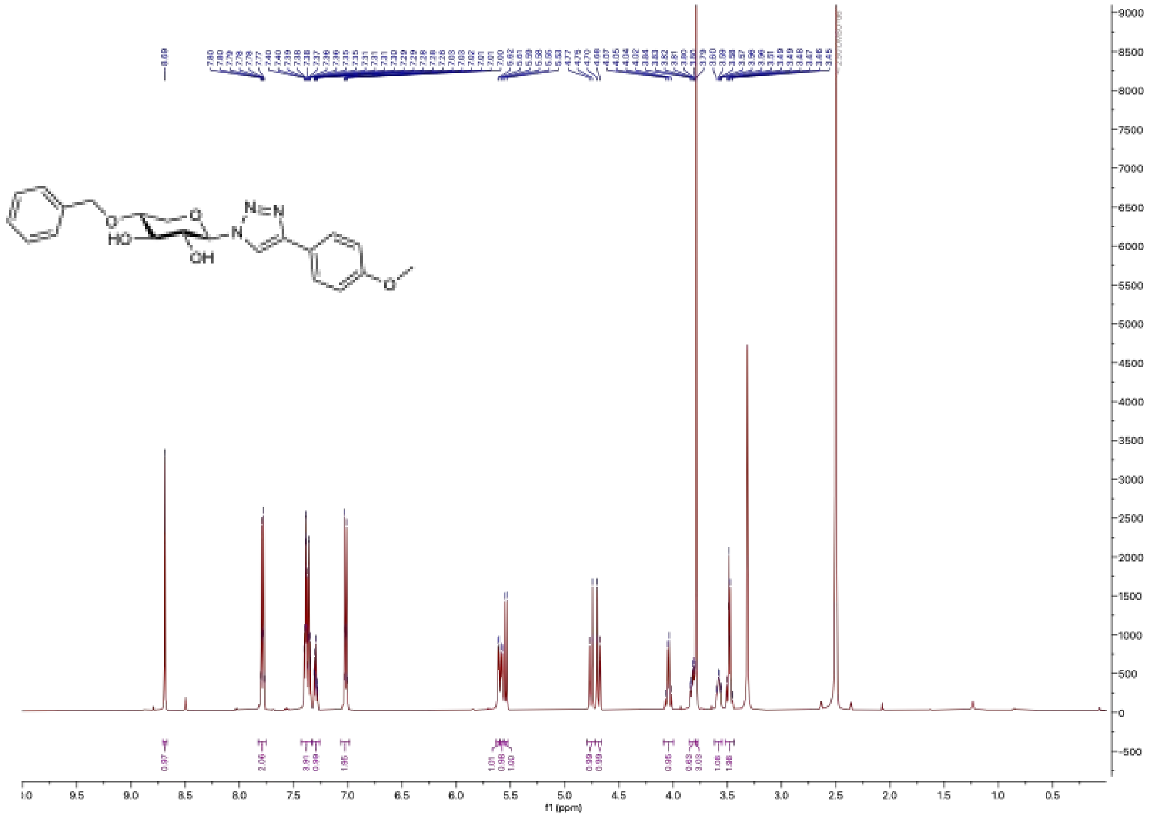

Figure S43: ^{1}H-NMR of compound **3**

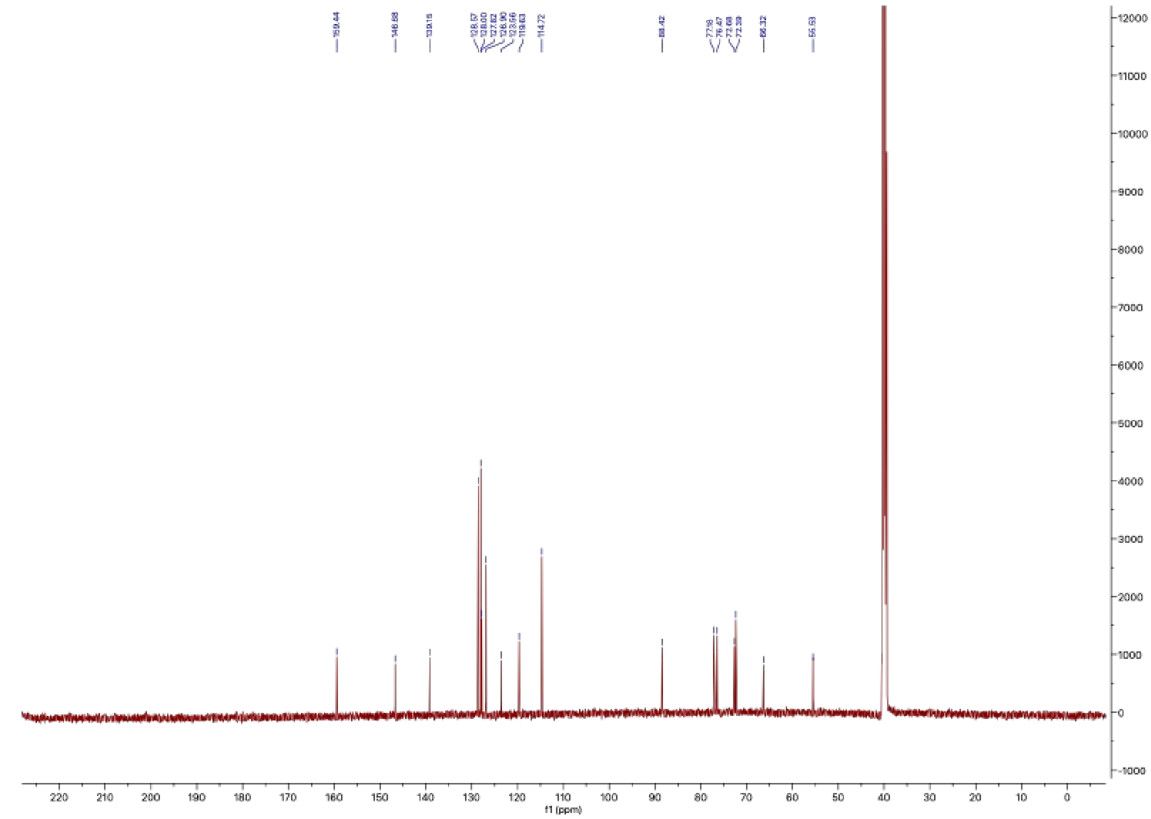

Figure S44: ^{13}C-NMR of compound **3**

^{1}H and ^{13}C spectra of compound 7

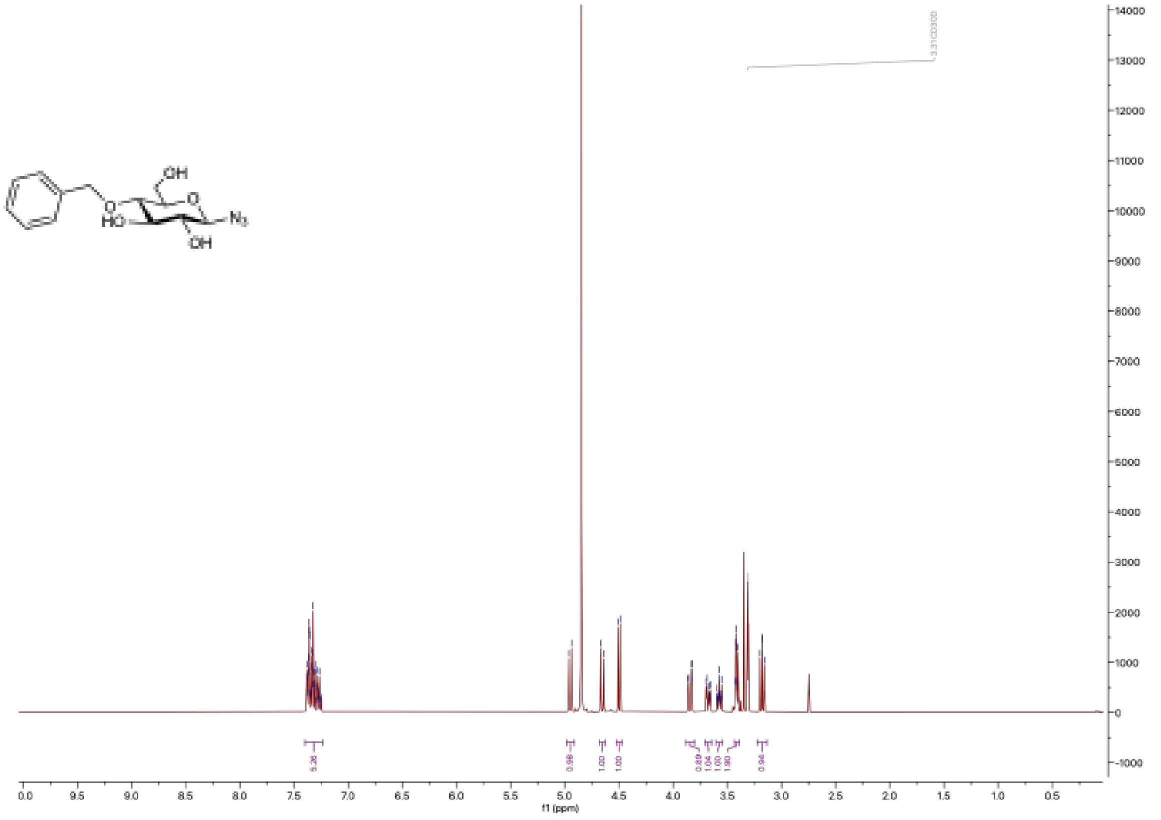

Figure S45: ^{1}H-NMR of compound **7**

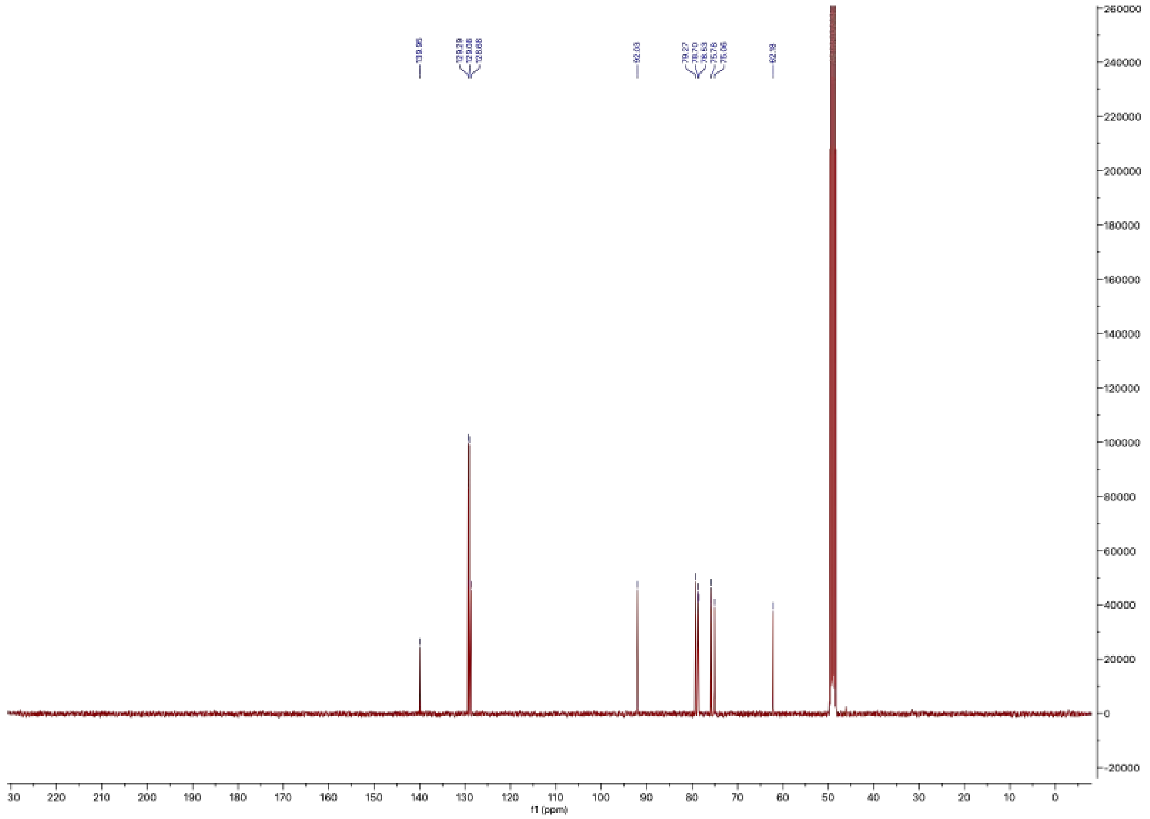

Figure S46: ^{13}C-NMR of compound **7**

^{1}H and ^{13}C spectra of compound 8

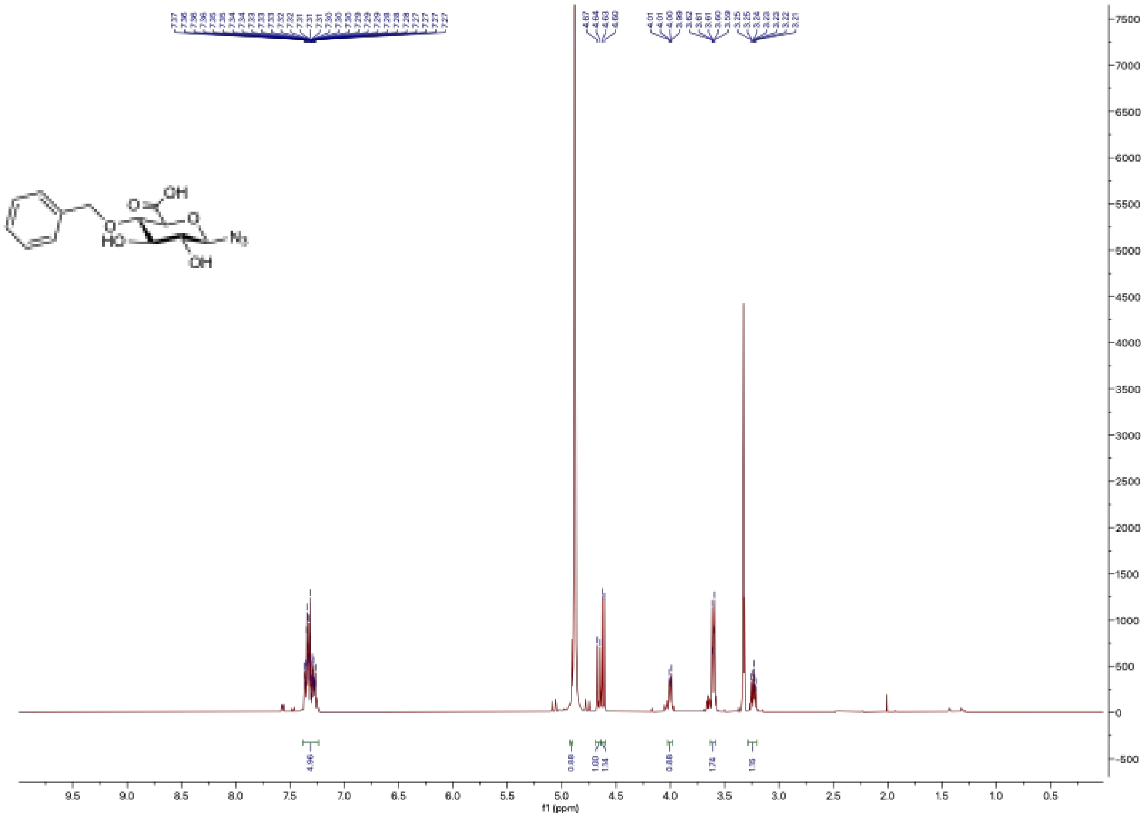

Figure S47: ^{1}H-NMR of compound **8**

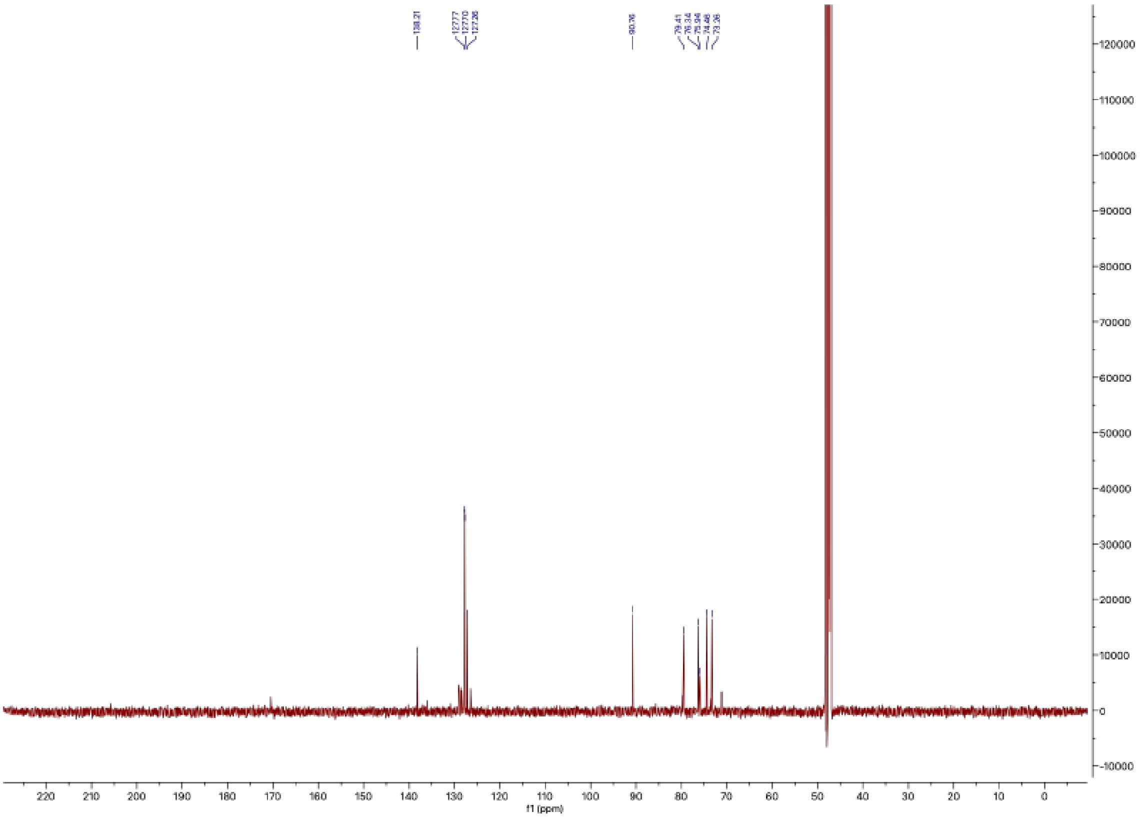

Figure S48: ^{13}C-NMR of compound **8**

^{1}H and ^{13}C spectra of compound 11

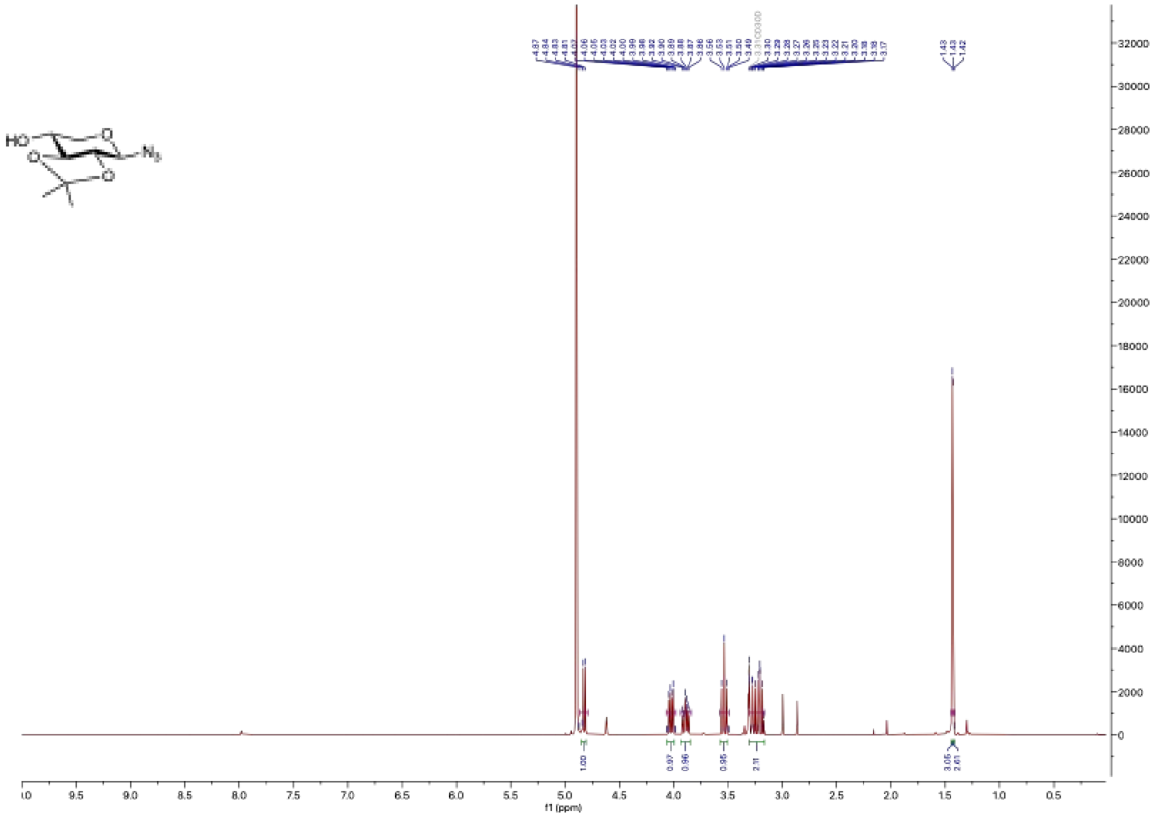

Figure S49: ^{1}H-NMR of compound **11**

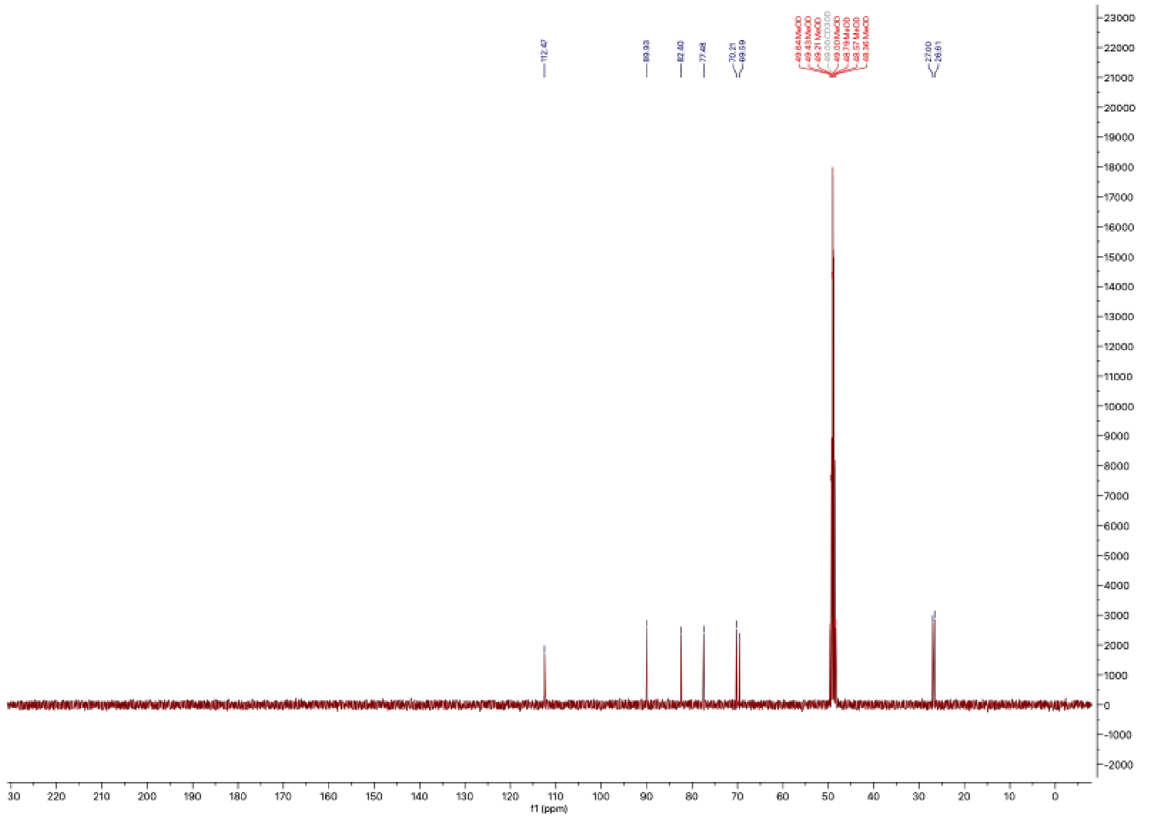

Figure S50: ^{13}C-NMR of compound **11**

^{1}H and ^{13}C spectra of compound 12

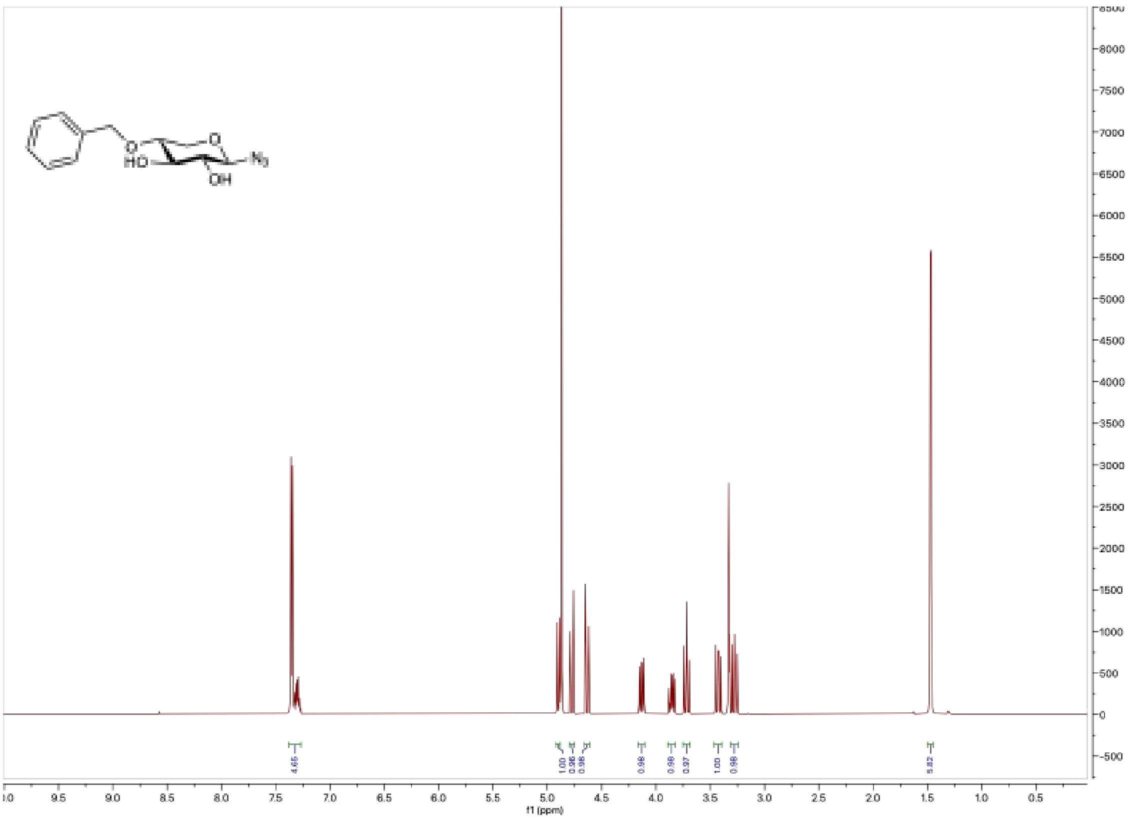

Figure S51: ^{1}H-NMR of compound **12**

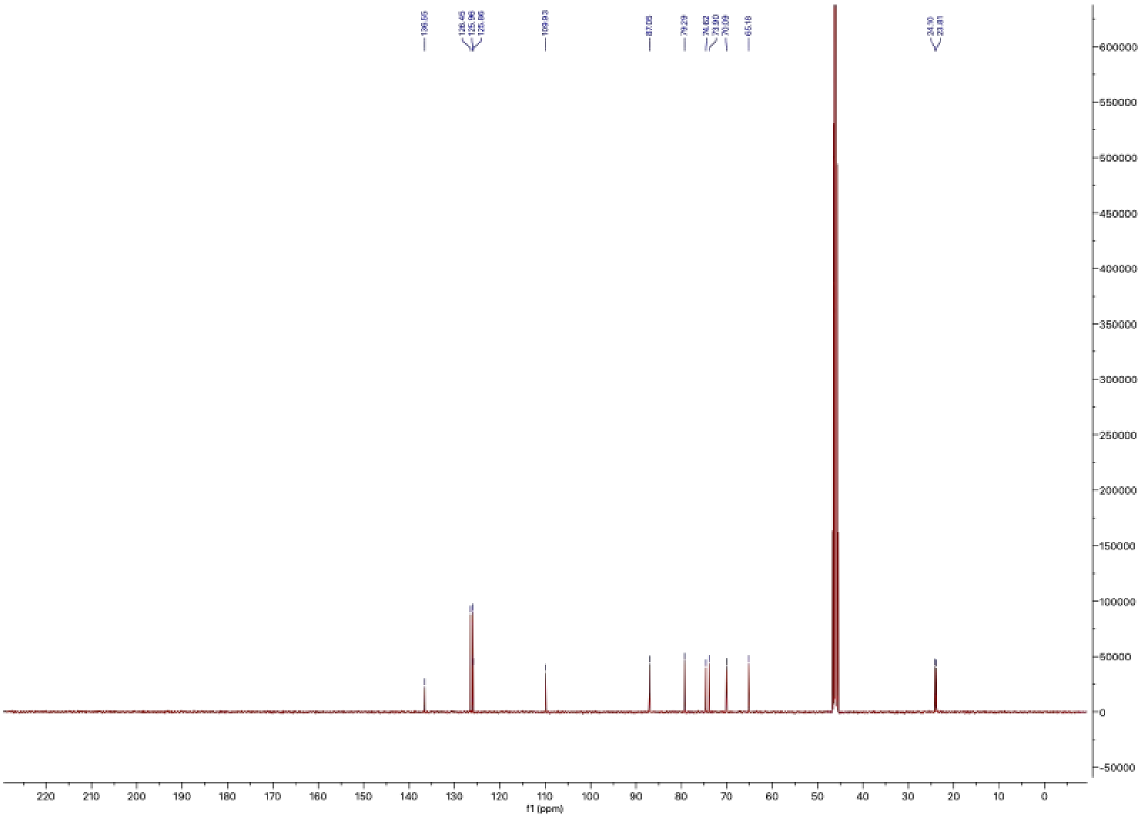

Figure S52: ^{13}C-NMR of compound **12**

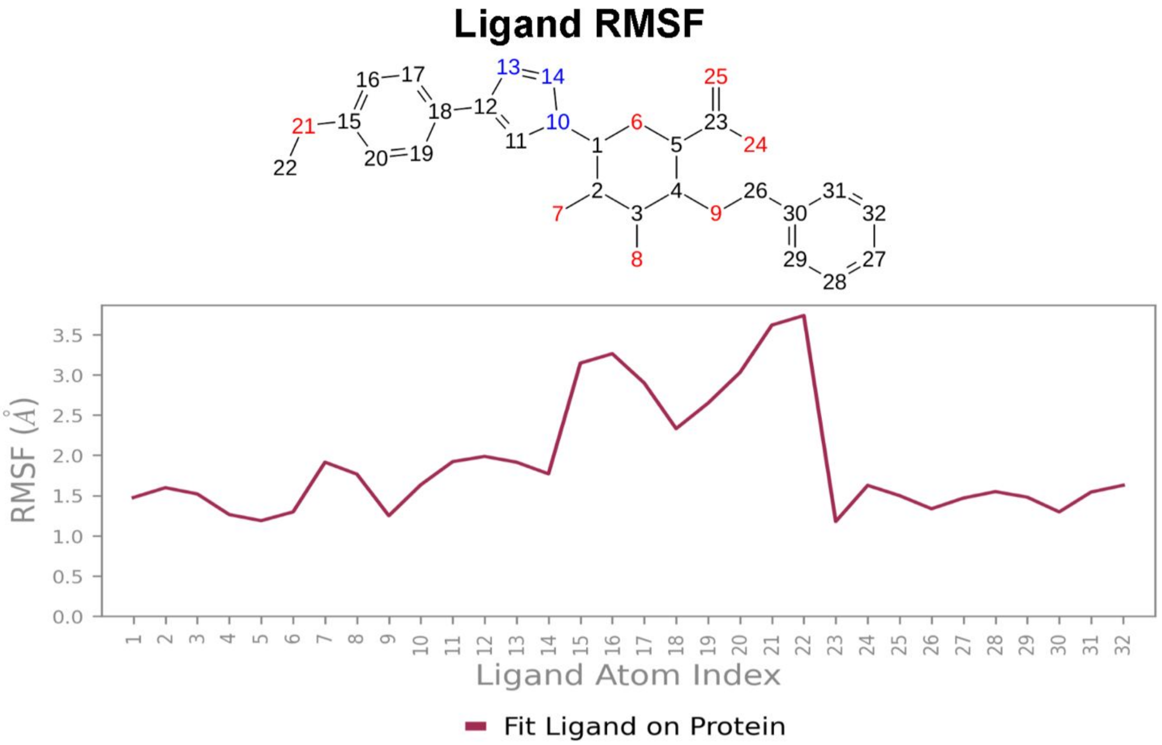

Figure S53: Docking studies of compound **1g**. RMSF of binding to protein.

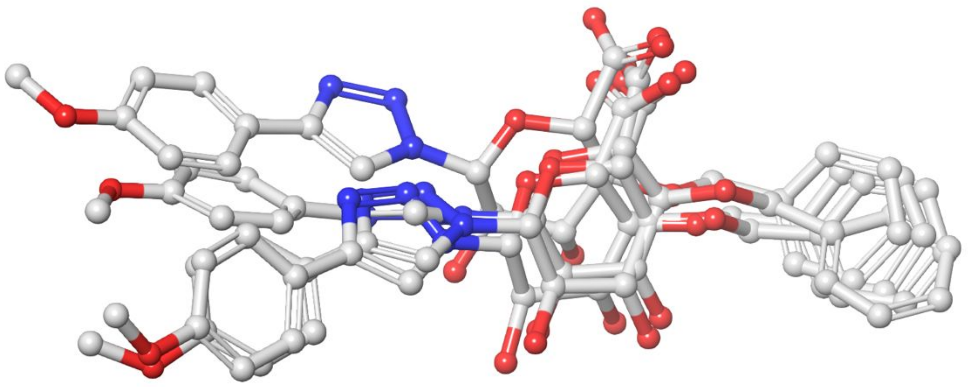

Figure S54: Docking studies of compound **1g**. Typical binding poses.

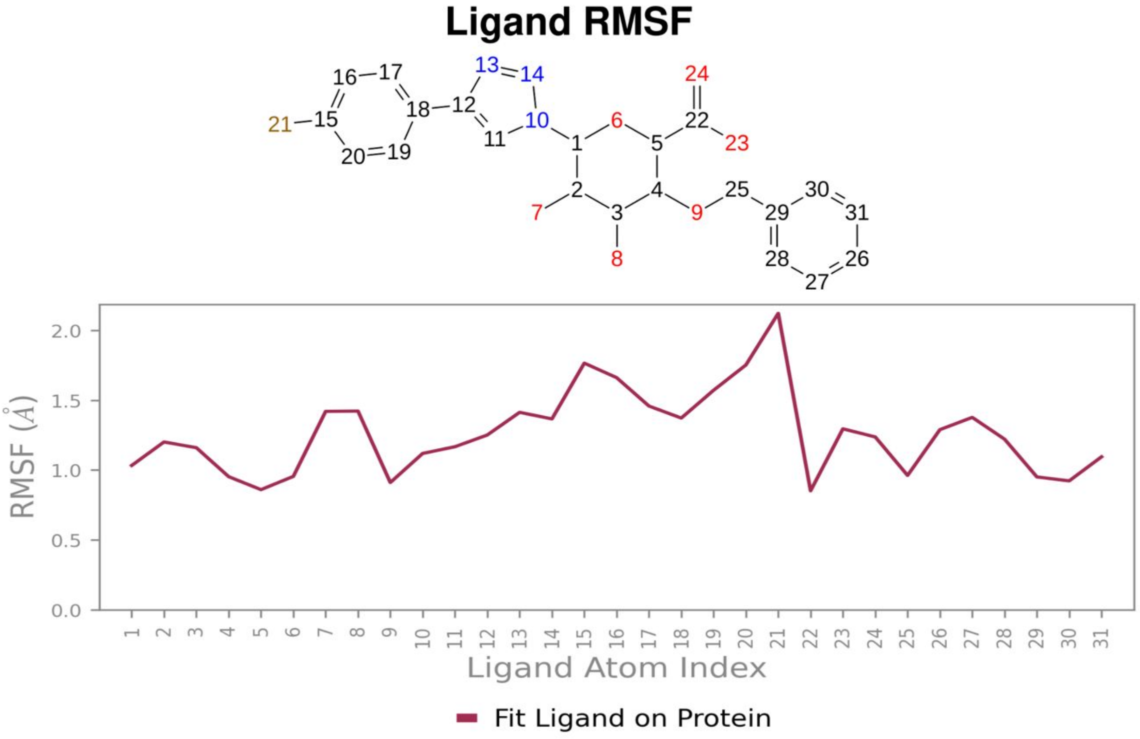

Figure S55: Docking studies of compound **1i**. RMSF of binding to protein.

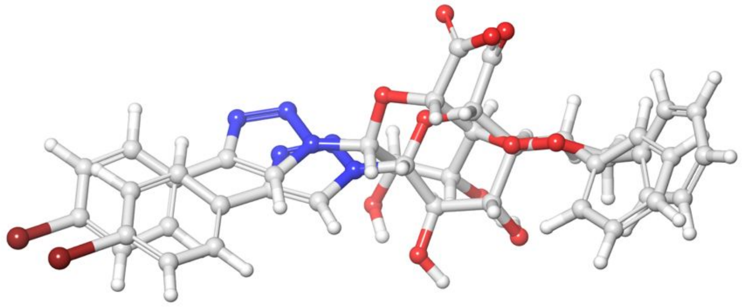

Figure S56: Docking studies of compound **1i**. Typical binding poses.
